# Network representations of angular regions for electromagnetic scattering

**DOI:** 10.1371/journal.pone.0182763

**Published:** 2017-08-17

**Authors:** Vito G. Daniele, Guido Lombardi, Rodolfo S. Zich

**Affiliations:** 1 Politecnico di Torino, Corso Duca degli Abruzzi 24, I-10129 Torino, Italy; 2 Istituto Superiore Mario Boella, via P.C. Boggio 61, I-10138 Torino, Italy; Lanzhou University of Technology, CHINA

## Abstract

Network modeling in electromagnetics is an effective technique in treating scattering problems by canonical and complex structures. Geometries constituted of angular regions (wedges) together with planar layers can now be approached with the Generalized Wiener-Hopf Technique supported by network representation in spectral domain. Even if the network representations in spectral planes are of great importance by themselves, the aim of this paper is to present a theoretical base and a general procedure for the formulation of complex scattering problems using network representation for the Generalized Wiener Hopf Technique starting basically from the wave equation. In particular while the spectral network representations are relatively well known for planar layers, the network modelling for an angular region requires a new theory that will be developed in this paper. With this theory we complete the formulation of a network methodology whose effectiveness is demonstrated by the application to a complex scattering problem with practical solutions given in terms of GTD/UTD diffraction coefficients and total far fields for engineering applications. The methodology can be applied to other physics fields.

## 1 Introduction

Network modeling has been widely used in electromagnetics since early developments of microwaves [1] and it is constitutive of the general theory of radiation and scattering as proposed by Marcuvitz and Felsen [2].

For what concerns the scattering by stratified planar media in presence of planar discontinuities a complete reference based on network formulations is [3].

Recently, geometries constituted of coupled angular and planar regions [4–10] have been studied through the Generalized Wiener Hopf technique introducing Laplace transform (LT) in radial direction, extending the class of solvable problems through the Wiener-Hopf (WH) technique, see [11] and references therein. Moreover the Wiener-Hopf technique is effectively applied to study problems with abrupt discontinuities in different physics [11] as acoustics, elasticity, fracture mechanics…

**Fig 1:**
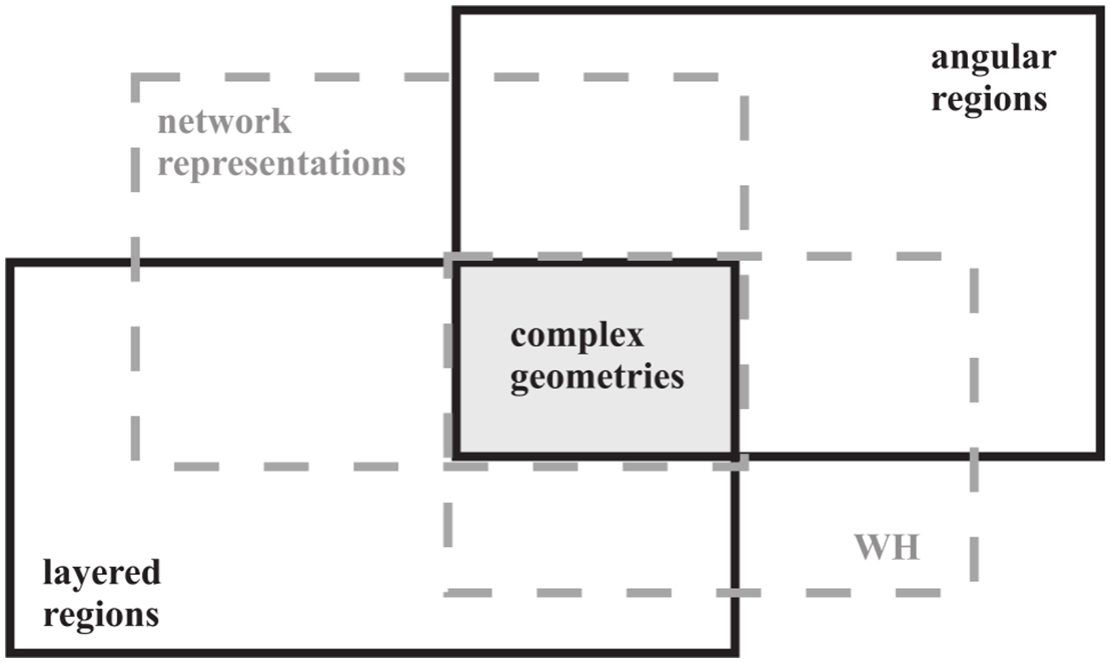
Concept of complex geometry identified by gray filled box as scattering problem constituted of coupled angular and layered regions (boxes with solid black contours); graphical interpretation of the technique proposed in the paper based on the connection among spectral representations of scattering problems through WH equations and network representations (boxes with dashed gray contours).

The use of the Wiener-Hopf technique in complex structures highlights the relevance of network representations. During these studies it became evident that more effort has to be expressed to completely master the network representation of an angular region that can be combined with the representations of planar regions to model complex problems: this is the aim of the paper. The logical framework of the paper is sketched in Fig 1 where the considered geometries are presented together with the relationship among the spectral representations of the scattering problem.

Network modelling of angular regions was firstly proposed in [2] where the representations were obtained using the classical separation variable method [12] through the definition of Green’s functions in natural domain (cylindrical coordinates *ρ*, *φ*, *z*). However, this method is effective only for particular boundary conditions as reported in Sec. 6.6 of [2], where the conclusion is that the general angular region problem *“requires more sophisticated mathematical techniques”*.

Pure isolated angular geometries (wedges) are effectively approached in literature by spectral representations. An exhaustive analysis of literature shows that the most important representations are the one formulated in terms of Sommerfeld Malyuzhinets (SM) functions and the one based on the Kontorovich-Lebedev (KL) transform. The introduction of the Generalized Wiener Hopf (GWH) technique for angular region [13] completes the spectral methods.

The three mentioned methods are defined in specific spectral (transformation) domains, see Section 3, which are in mutual relations:

- the Sommerfeld-Malyuzhinets (SM) function method with spectral complex plane *w* [14–21]
- the Kontorovich-Lebedev (KL) transform method with spectral complex plane *t* [22–25]
- methods based on the Laplace transform (LT) with spectral complex plane *η* [11, 13, 26–31], that include the GWH technique.

In the above itemization we have reported a sample of literature related to the three methods: the original works together with review works.

**Fig 2:**
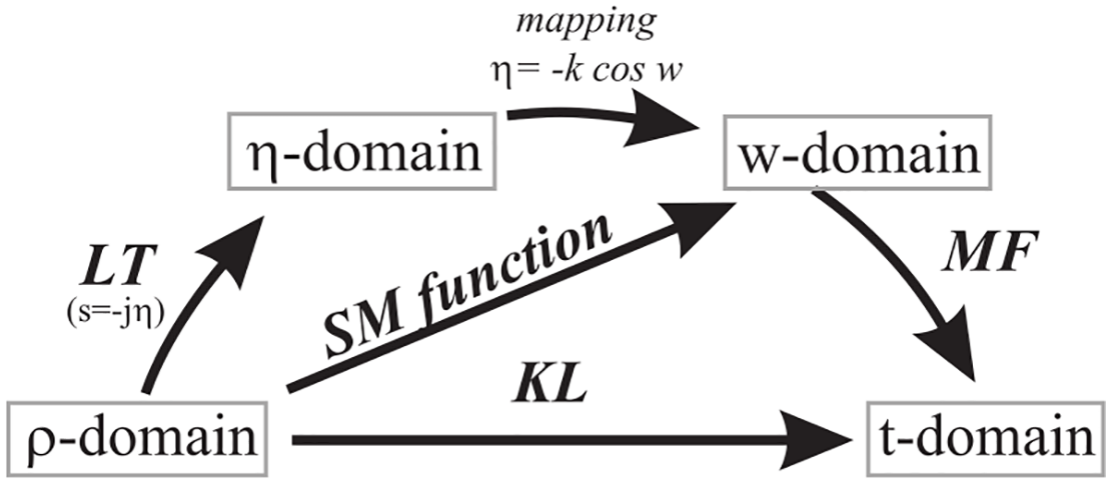
Graphical resume of natural plane (*ρ*) and complex spectral planes (*η*, *w*, *t*) considered in the paper.

These representations are interrelated see Fig 2. In particular the relationship between *η* and *w* plane is through the mapping *η* = −*k* cos *w* with propagation constant *k* and the relationship between *w* and *t* planes is through the Malyuzhinets-Fourier (MF), see Section 3 for a detailed definition and discussion.

We state that each representation presents advantages and disadvantages which are illustrated in this paper.

In fact while the network representations in the planar layers are obtainable working only in the framework of the Laplace transforms (see [3–5] and deduction of network representation of planar layer region 2 in Section 7), the general problem of angular region can not be easily studied by using the properties of a single spectral plane. We present in this paper a procedure that takes advantage of the formulations given in each one of the defined spectral planes, although our aim is to present modeling and solutions that privileges the use of Laplace Transform. In the following the complex plane of Laplace Transform is labeled with *η*. In the paper we make also reference to SM functions and to the KL transform where we label the respective spectral planes with *w* and *t*. The adopted notations are strongly influenced from the fundamental book on radiation and scattering [2] whose notations are different from those used by authors specialized in the SM method.

The use of *η* plane is of great importance: 1) it allows to define the entire problem in a unique spectral plane when different materials coexist and 2) it allows to handle angular regions with any aperture angles directly in terms of integral equations. In particular property 1) yields that the unknowns are defined in the sample complex plane *η* since *η* plane does not depend on the material and thus there is no need to define cumbersome rule to couple the unknowns defined in different complex planes at the material interfaces. On the contrary *w* plane depends on the material through the local propagation constant (see next Section 3 for definition).

In spectral domain the network modelling is an effective technique to represent complex problems and in this paper we propose an example of procedure to obtain network modelling for the scattering by wedge structures also in presence of layers.

Network modeling mathematically rephrases spectral representations and it yields several advantages:

1. Equivalent network modeling of a complex problem allows a direct and quick pictorial interpretation of the interaction among structures that constitute the original problem.
2. The unknowns of the electromagnetic problem are directly related to currents and voltages of the network model.
3. Each part of a complex electromagnetic structure can be identified and modeled as a circuital component/network whose parameters are evaluated once and for all.
4. The interaction by different part of the problems is enforced by current and voltage continuity at the ports of the equivalent networks.
5. Circuit analysis helps to order and to systematize the steps necessary to obtain the equations that model the complex problem avoiding redundancy.

**Fig 3:**
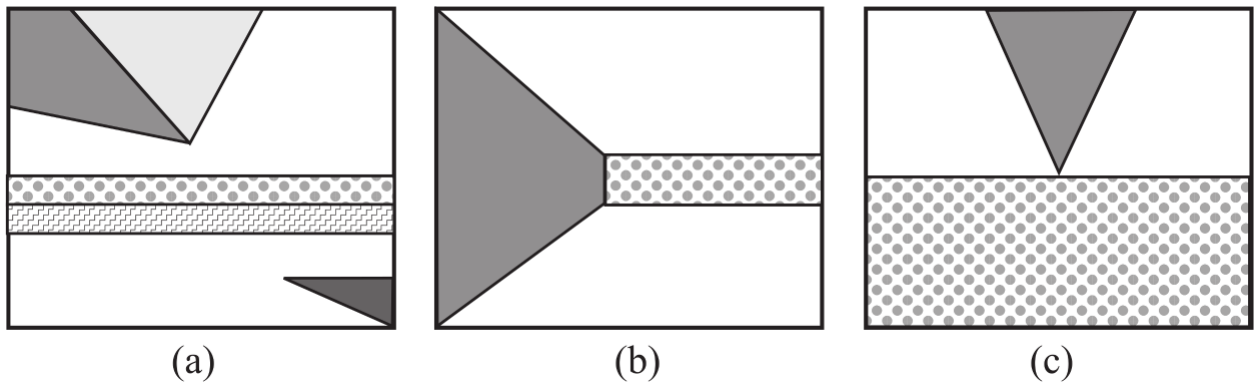
Example of WH geometries modelled by network representations: (a) angular regions in presence of a stratification, (b) truncated wedge with a semi-infinite layer, (c) wedge over a penetrable half space.

Even if the network representations in spectral planes is of great importance by itself, the aim of this paper is to present a theoretical base and a general procedure for the formulation of complex problems using network representation for the Wiener Hopf (WH) Technique. Fig 3 reports a list of examples of problems that can be formulated and studied using Generalized Wiener Hopf (GWH) technique.

The solution is usually obtained via the reduction of the GWHEs to a system of coupled Fredholm integral equations (FIEs) [32, 33] via the elimination of one of the unknowns in the network model. With this paper we state that the FIEs derived for GWHEs can be reframed using network representations and involve kernels expressed in terms of elementary functions. A novel practical example is outlined in Section 7.

The aim of this paper is to simplify and to systematically present the procedure to model electromagnetic scattering problems in angular regions by resorting to network modeling in *w* and *η* planes starting from GWH formulations in *η* plane.

However, the procedure reported in this paper starts from the wave equations in cylindrical coordinates (Section 4) and following the rotating waves method [34] yields to the network modeling of angular regions in *t*, *w* planes (Sections 4 and 5) and finally in *η* plane (Section 6).

Although the same equations are obtainable from GWH formulation [13] by using the Fredholm factorization [4, 32, 33, 35–37], here we have privileged the use of rotating waves method because the transmission line model and thus the network model is straightforward in this paradigm. Moreover rotating waves method works directly in *w* plane and as stated in [2] this complex plane does not hide any spectral properties of the problem with respect to *η* plane (proper and improper sheets defined with respect to the $\xi =\sqrt{k^{2}-\eta^{2}}$ function).

The paper is focused on the Norton two-port representation, but other two-port representations are possible (for instance the Thevenin model) following the same procedure. For space reason this item is not considered in this paper. However the Norton representations of angular regions and layered regions provide the formulation in terms of FIEs of electromagnetic problems with geometries like those illustrated in Fig 3.

We will show in Section 4.3 that the network parameters of the two port in t-plane are expressed by elementary functions. A difficult task is to get the representations in *η*-plane, starting from these simple expressions.

The paper is divided into seven Sections, including the Introduction. Section 2 presents how to model angular regions through two port Norton networks in abstract space and introduces the spectral planes and the notation used in this paper. Section 3 presents the principal spectral domains in use to deal with scattering problem in electromagnetics. Section 4 estimates the parameter of the Norton network in absence of source, in presence of concentrated sources and for an incident plane wave. Section 5 analyzes the case of an angular region delimited by an impenetrable surface and presents the one port Norton network in particular for regions terminated by a perfect electric conducting (PEC) surface. Section 6 presents the procedure to obtain FIEs for angular regions in *η* plane, in particular for the PEC wedge. The methodology is general and the equations are fully validated by numerous examples.

Throughout the paper, we focus the implementation on problems constituted of PEC wedge surrounded by different objects/materials where the Norton representation is particularly effective. Extension to surface impedance structures and penetrable structures is possible and of practical interest. Moreover other two port network modelling can be derived (for instance the Thevenin model).

Finally, we validate the proposed method in Section 7 by presenting a novel complex problem. The problem is constituted by the scattering of an *E*_*z*_ polarized plane wave by a PEC wedge above a PEC plane that recalls a horn antenna. The results show the capability of network modelling to deal with complex problems and show the convergence, the efficiency and the efficacy of the proposed method estimating the electromagnetic far field in terms of Geometrical Optics (GO) components and Uniform Theory of Diffraction (UTD) components.

The solution procedure is based on coupling network representations of angular and planar layered regions in *η* plane. The network representation of the angular region in *η* plane is obtained in this paper starting from the wave equation (Section 4) and following the procedure proposed in Sections 5 and 6. The planar layered region is described directly through an example in Section 7 since it is derived from the theory of planar stratified region described in [3].

To improve the readability of the manuscript we have added an Appendix and a list of conventions/notations/symbols at the end of the manuscript.

## 2 Norton two-port network for an angular region in abstract space

Fig 4 presents an example of space subdivided into angular regions with arbitrary aperture angles. Each cylindrical sector is filled by a linear medium. Without loss of generality, let us focus the attention on the region delimited by the half planes *φ* = *φ*_1_ and *φ* = *φ*_2_ where a distributed source S is located. Cartesian coordinates (*x*, *y*, *z*) as well as polar coordinates (*ρ*, *φ*, *z*) will be used to describe the problem.

**Fig 4:**
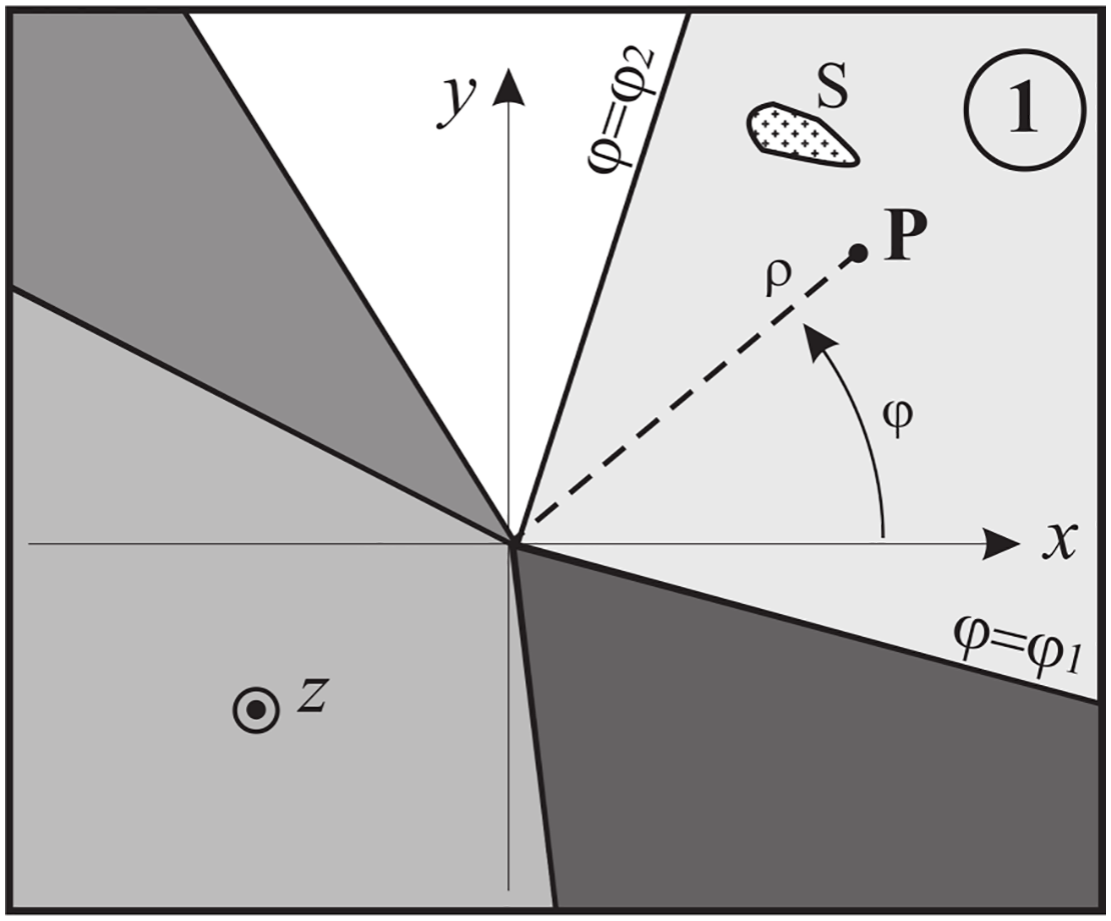
Arbitrary angular regions filled by linear media. In Section 3, 4, 5 we make reference to the angular region 1 delimited by *φ* = *φ*_1_ and *φ* = *φ*_2_ with a distributed source S.

Resorting to the uniqueness theorem, we state that two relations must connect the electromagnetic field components tangent to the interfaces. These to-be determined relations are constitutive of a two port network representing an arbitrary angular region. Fig 5a graphically represents the two-port network in an abstract space [38] where the electromagnetic field components tangent to the interfaces *φ* = *φ*_1_ and *φ* = *φ*_2_ are labeled respectively $E_{t1},H_{t1}$ and $E_{t2},H_{t2}$.

**Fig 5:**
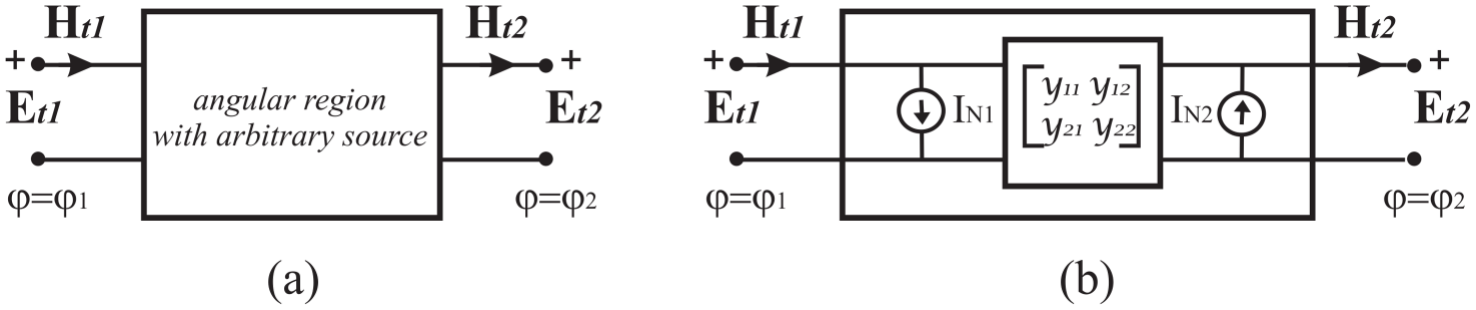
Network representation of the angular region 1 of Fig 4 in an abstract space. (a) Undefined representation, (b) Norton representation.

Since the problem is linear (linear media), two-port network representations are possible. Fig 5b shows, in particular, the Norton representation where the equivalent Norton currents $I_{N1},I_{N2}$ represent $H_{t1},H_{t2}$ when two PEC half-planes are located at *φ* = *φ*_1_ and *φ* = *φ*_2_.

In abstract operator algebra, the parameter of the Norton representation *y*_*ij*_ are abstract operators [38].

The explicit determination of parameters in a two-port network model is possible by selecting an explicit function space (see Section 3) to represent the unknowns, i.e. the physical quantities. In particular we anticipate the result that in the KL t-space the Norton representation is algebraic, although in other spaces the representation is defined through integral operators.

As stated in the introduction, the natural domain does not allow to model the general angular region problem and, in particular, even the evaluation of Norton currents constitutes a cumbersome electromagnetic problem. The problem simplifies if one uses spectral representations of the electromagnetic fields. With reference to Fig 4, for the sake of simplicity to highlight the procedure, we consider in this paper the case with cylindrical symmetry, i.e. the two dimensional case, with a *E*_*z*_-polarized electric field, for example excited by a distributed source S constituted by an electric current density *J*_*z*_(*ρ*, *φ*). Since the geometry of the problem is invariant with respect to the z direction, the three dimensional case does not introduce new conceptual difficulties but simply increases the number of the unknowns and equations.

## 3 Spectral domains for scattering problem in electromagnetics

Considering time harmonic electromagnetic fields with a time dependence specified by the factor *e*^*jωt*^ (which is omitted) and with reference to Fig 4, the formulation of the angular region problem for a *E*_*z*_-polarized electric field in spectral domain starts from the definition of the Laplace transforms of electromagnetic field components:

$$\begin{array}{c} {\dot{V}}_{+}\left(s,\varphi \right)=\int_{0}^{\infty }E_{z}\left(\rho ,\varphi \right)e^{-s\rho }d\rho ,\;{\dot{I}}_{+}\left(s,\varphi \right)=\int_{0}^{\infty }H_{\rho }\left(\rho ,\varphi \right)e^{-s\rho }d\rho \end{array}$$ (1)

In the following we will make extensive use of the Wiener-Hopf quantities defined in the *η* = *js* plane, i.e. $F\left(\eta ,\varphi \right)=\dot{F}\left(s=-j\eta ,\varphi \right)$.

To avoid the presence of singularities on the real axis of *η* = *js* plane, the propagation constants *k* are assumed with a negative (vanishing) imaginary part. Using the framework of Wiener-Hopf technique, the subscript + in Eq (1) indicates plus functions, i.e. functions having regularity half-planes that are upper half-planes in the *η*-plane. Conversely the subscript—indicates minus functions, i.e. functions having regularity half-planes that are lower half-planes in the *η*-plane. In the following we will make extensive use of the spectral quantities Eq (1) defined for *φ* = *φ*_1_ and *φ* = *φ*_2_, that we label

$$\begin{array}{c} \begin{array}{c} V_{q+}\left(\eta \right)=V_{+}\left(\eta ,\varphi_{q}\right)={\dot{V}}_{+}\left(s,\varphi_{q}\right)\\ I_{q+}\left(\eta \right)=I_{+}\left(\eta ,\varphi_{q}\right)={\dot{I}}_{+}\left(s,\varphi_{q}\right) \end{array},\;q=1,2 \end{array}$$ (2)

In this paper, we first derive a general procedure to obtain the network model by using the angular complex plane *w*

$$\begin{array}{c} \eta =-k\cos w \end{array}$$ (3)

starting from the complex plane *t* defined through the Malyuzhinets-Fourier (MF) transform and the Kontorovich-Lebedev (KL) transform, see below for a complete definition.

The benefits of the complex plane *w* are described in several works, see for example [2, 3, 13, 34]. In particular, we remark the following properties:

- a plus function in the *η*-plane is an even function of *w*
- transmission line equations hold for the unknowns in *w*-plane
- the Sommerfeld functions are defined in *w*-plane
- in *w*-plane the Norton parameters are operators that do not involve multivalued functions, on the contrary multivalued functions are to be defined in *η* plane

In the following we apply the notations where we label the quantities in the spectral domain without any sign on top of the variable when the quantity is defined in *η* plane, with ⋅ (dot), ^ (hat) or − (overline) on top of the variable when the quantity is defined respectively in *s*, *w* or *t* plane, and with ∼ (tilde) on top of the variable if we make abstractly reference to a general spectral domain (*η*, *s*, *w* or *t*).

In *w* plane we define

$$\begin{array}{c} \begin{array}{cc} {\hat{V}}_{d}\left(w,\varphi \right)=\sin w\;{\hat{V}}_{+}\left(w,\varphi \right)=\sin w\;V_{+}\left(-k\cos w,\varphi \right), & {\hat{I}}_{+}\left(w,\varphi \right)=I_{+}\left(-k\cos w,\varphi \right)\\ {\hat{V}}_{qd}\left(w\right)=\sin w\;{\hat{V}}_{q+}\left(w\right)=\sin w\;V_{q+}\left(-k\cos w\right), & {\hat{I}}_{q+}\left(w\right)=I_{q+}\left(-k\cos w\right) \end{array} \end{array}$$ (4)

with *q* = 1, 2.

In particular we define the two-port network in *w* plane in terms of the voltages ${\hat{V}}_{qd}\left(w\right)$ and the currents ${\hat{I}}_{q+}\left(w\right)$ with *q* = 1, 2.

We introduce the Malyuzhinets-Fourier (MF) transform that relates quantities defined in the complex plane *w* to quantities in the complex plane *t* [34]

$$\begin{array}{c} M\left\{\hat{F}\left(w\right)\right\}=\bar{F}\left(t\right)=-j\int_{-j\infty }^{j\infty }\hat{F}\left(w\right)e^{+jtw}dw,\;\text{Re}\left[t\right]=0 \end{array}$$ (5)

$$\begin{array}{c} \hat{F}\left(w\right)=M^{-1}\left\{\bar{F}\left(t\right)\right\}=-\frac{j}{2\pi }\int_{-j\infty }^{j\infty }\bar{F}\left(t\right)e^{-jtw}dt,\;\text{Re}\left[w\right]=0 \end{array}$$ (6)

This transform must be defined in the distribution space, thus the integrals in Eqs (5) and (6) are Cauchy principal values. The main benefit of the complex plane t is that the Norton model is constituted of algebraic parameters, as demonstrated in the Section 4.

Relations among natural domain in *ρ*, Laplace Transform (LT) in *η*, the complex plane *w* (*η* = −*k* cos *w*), the Malyuzhinets-Fourier (MF) transform in *t*, the Kontorovich-Lebedev (KL) transform and the Sommerfeld-Malyuzhinets (SM) function are deeply studied in [34] and we graphically resume these relations in Fig 2.

## 4 Evaluation of the Norton network in spectral domains

**Fig 6:**
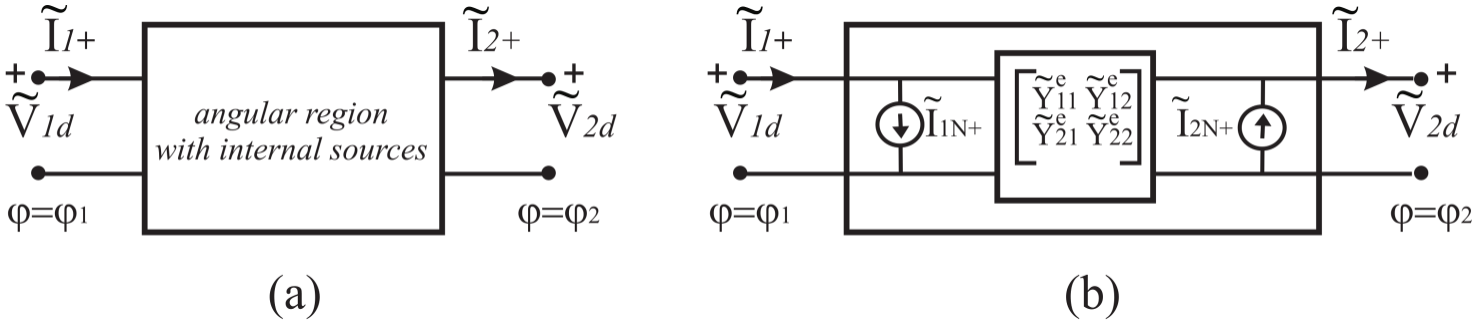
Network representation of the angular region in a spectral domain. (a) Undefined representation, (b) Norton representation.

**Fig 7:**
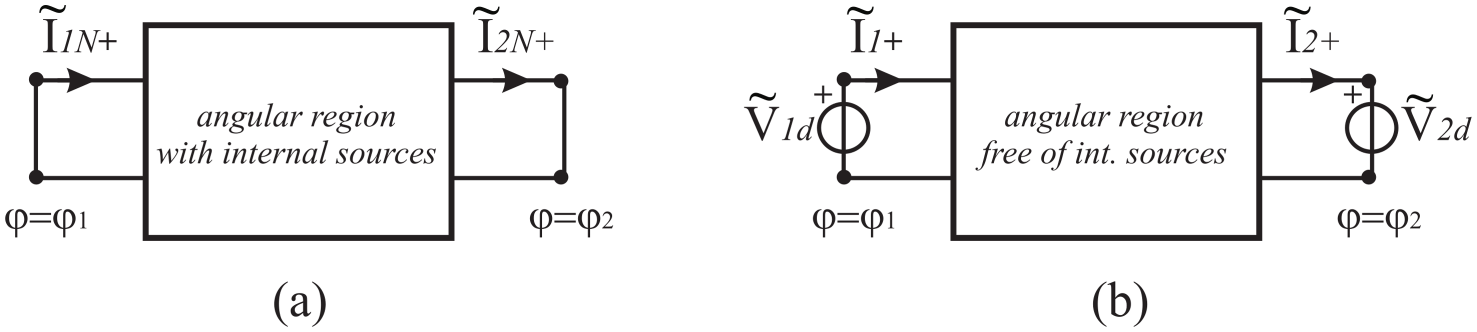
Evaluation of the Norton parameters of the two-port network representing the angular region in a spectral domain. (a) Norton currents, (b) Norton admittances.

Let us consider the homogenous angular region *φ*_1_ < *φ* < *φ*_2_ with a distributed source modelled by *J*_*z*_(*ρ*, *φ*), see Fig 4. As already stated in the natural domain, taking into account the uniqueness theorem, two equations must relate the four quantities ${\tilde{V}}_{qd},{\tilde{I}}_{q+}$ (*q* = 1, 2) also in spectral domains. Fig 6a graphically represents the two-port network in an abstract spectral domain. Fig 6b illustrates the Norton representation

$$\begin{array}{c} \left(\begin{array}{c} {\tilde{I}}_{1+}\\ -{\tilde{I}}_{2+} \end{array}\right)=\left[\begin{array}{cc} {\tilde{Y}}_{11}^{e} & {\tilde{Y}}_{12}^{e}\\ {\tilde{Y}}_{21}^{e} & {\tilde{Y}}_{22}^{e} \end{array}\right]\left(\begin{array}{c} {\tilde{V}}_{1d}\\ {\tilde{V}}_{2d} \end{array}\right)+\left(\begin{array}{c} {\tilde{I}}_{1N+}\\ -{\tilde{I}}_{2N+} \end{array}\right) \end{array}$$ (7)

where the vectors ${\tilde{I}}_{1N+},{\tilde{I}}_{2N+}$ are the short-circuit currents on the two-port network and the matrix is the admittance matrix of the two-port network that is in general constituted of operator elements. The procedure to obtain the Norton parameters is illustrated in Fig 7 and it is similar to and based on the classical approach of circuit theory.

As already discussed for the abstract space, we obtain the Norton equivalent currents by introducing two PEC half planes at *φ* = *φ*_1_ and *φ* = *φ*_2_. With reference to Fig 7a, according to Eqs (1) and (2), in general in a spectral domain, the PEC constraint for *E*_*z*_-polarization is equivalent to short-circuit the two-port of the equivalent network (i.e. ${\tilde{V}}_{1d}=0$ and ${\tilde{V}}_{2d}=0$), yielding the required equivalent Norton currents ${\tilde{I}}_{1N+}$ and ${\tilde{I}}_{2N+}$. As classically done in circuit theory, the admittance parameters are obtained by removing internal sources and by feeding the two-port by two arbitrary voltage generator ${\tilde{V}}_{1d}$ and ${\tilde{V}}_{2d}$. With reference to Fig 7b, we define the admittance parameters:

$$\begin{array}{c} {\tilde{I}}_{1+}={{\tilde{Y}}_{11}^{e}\;{\tilde{V}}_{1d}|}_{{\tilde{V}}_{2d}=0},\;{\tilde{I}}_{1+}={{\tilde{Y}}_{12}^{e}\;{\tilde{V}}_{2d}|}_{{\tilde{V}}_{1d}=0},\;{\tilde{I}}_{2+}=-{{\tilde{Y}}_{21}^{e}\;{\tilde{V}}_{1d}|}_{{\tilde{V}}_{2d}=0},\;{\tilde{I}}_{2+}=-{{\tilde{Y}}_{22}^{e}\;{\tilde{V}}_{2d}|}_{{\tilde{V}}_{1d}=0} \end{array}$$ (8)

**Fig 8:**
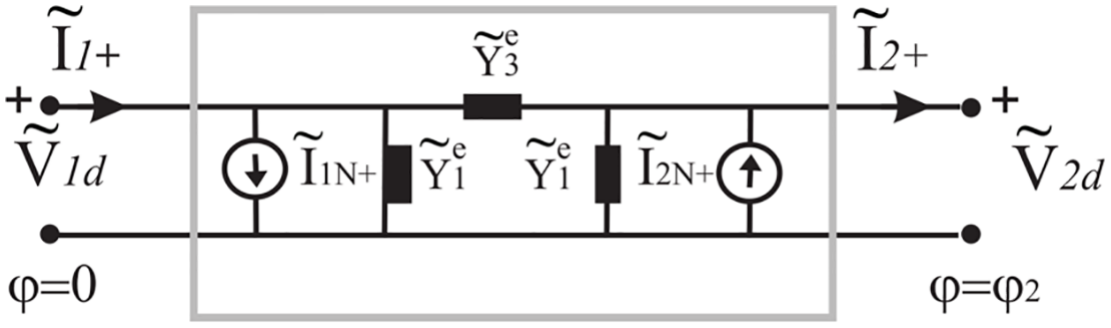
Norton *π* network in a spectral domain for the angular region *φ*_1_ < *φ* < *φ*_2_.

When the problem is reciprocal and symmetrical (${\tilde{Y}}_{12}^{e}={\tilde{Y}}_{21}^{e}$ and ${\tilde{Y}}_{22}^{e}={\tilde{Y}}_{11}^{e}$), the Norton representation can be represented through a *π* network, see Fig 8, where ${\tilde{Y}}_{1}^{e}={\tilde{Y}}_{11}^{e}+{\tilde{Y}}_{12}^{e}$ and ${\tilde{Y}}_{3}^{e}=-{\tilde{Y}}_{12}^{e}$. We recall that in general ${\tilde{Y}}_{ij}^{e}$ with *i*, *j* = 1, 2 are mathematical operator.

For the actual computation of the network, we first consider the wave equation in cylindrical coordinates with a current density source *J*_*z*_(*ρ*, *φ*) that does not depend on *z*:

$$\begin{array}{c} \frac{\partial }{\partial \rho }\left(\rho^{2}\frac{\partial^{}E_{z}}{\partial \rho^{}}\right)-\rho \frac{\partial E_{z}}{\partial \rho }+\frac{\partial^{2}E_{z}}{\partial \varphi^{2}}+k^{2}\rho^{2}E_{z}=jkZ_{o}\rho^{2}J_{z}\left(\rho ,\varphi \right) \end{array}$$ (9)

where *Z*_*o*_ and *k* are the impedance and the propagation constant of the free space.

By applying the Laplace transform Eq (1) and its properties (i.e. *L*[*f*(*ρ*)] = *F*[*s*], $L\left[\frac{\partial f(\rho )}{\partial \rho }\right]=sF\left[s\right]-f\left(0\right)$, $L\left[\rho f\left(\rho \right)\right]=-\frac{\partial F[s]}{\partial s}$) to Eq (9) and taking into account Meixner conditions [39]$\rho^{2}\frac{\partial E_{z}\left(\rho ,\varphi \right)}{\partial \rho }\to 0$ and $\frac{\partial E_{z}\left(0,\varphi \right)}{\partial s}=0$ for *ρ* → 0, we obtain

$$\begin{array}{c} s\frac{\partial^{2}}{\partial s^{2}}\left(s{\dot{V}}_{+}\left(s,\varphi \right)\right)+\frac{\partial }{\partial s}\left(s{\dot{V}}_{+}\left(s,\varphi \right)\right)+\frac{\partial^{2}{\dot{V}}_{+}\left(s,\varphi \right)}{\partial \varphi^{2}}+k^{2}\frac{\partial^{2}}{\partial s^{2}}{\dot{V}}_{+}\left(s,\varphi \right)=jkZ_{o}\frac{\partial^{2}}{\partial s^{2}}{\dot{J}}_{z}\left(s,\varphi \right) \end{array}$$ (10)

and thus:

$$\begin{array}{c} \left(k^{2}+s^{2}\right)\frac{\partial^{2}}{\partial s^{2}}{\dot{V}}_{+}\left(s,\varphi \right)+3s\frac{\partial }{\partial s}{\dot{V}}_{+}\left(s,\varphi \right)+{\dot{V}}_{+}\left(s,\varphi \right)+\frac{\partial^{2}{\dot{V}}_{+}\left(s,\varphi \right)}{\partial \varphi^{2}}=jkZ_{o}\frac{\partial^{2}}{\partial s^{2}}{\dot{J}}_{z}\left(s,\varphi \right) \end{array}$$ (11)

i.e.

$$\begin{array}{c} \frac{\partial }{\partial s}\left[\left(k^{2}+s^{2}\right)\frac{\partial }{\partial s}{\dot{V}}_{+}\left(s,\varphi \right)\right]+\frac{\partial }{\partial s}\left(s{\dot{V}}_{+}\left(s,\varphi \right)\right)+\frac{\partial^{2}{\dot{V}}_{+}\left(s,\varphi \right)}{\partial \varphi^{2}}=jkZ_{o}\frac{\partial^{2}}{\partial s^{2}}{\dot{J}}_{z}\left(s,\varphi \right) \end{array}$$ (12)

Recalling that *s* = −*jη* and *η* = −*k* cos *w* and taking into account that $\frac{\partial }{\partial s}=\frac{1}{-jk\sin w}\frac{\partial }{\partial w}$ and *k*^2^ + *s*^2^ = *k*^2^ sin^2^ *w*, Eq (12) becomes

$$\begin{array}{c} \frac{-1}{\sin w}\frac{\partial }{\partial w}\left(\sin w\frac{\partial }{\partial w}{\hat{V}}_{+}\left(w,\varphi \right)\right)+\frac{-1}{\sin w}\frac{\partial }{\partial w}\left(\cos w{\hat{V}}_{+}\left(w,\varphi \right)\right)+\frac{\partial^{2}{\hat{V}}_{+}\left(w,\varphi \right)}{\partial \varphi^{2}}=jkZ_{o}\frac{\partial^{2}}{\partial s^{2}}J_{z}\left(s,\varphi \right) \end{array}$$ (13)

Introducing ${\hat{V}}_{d}\left(w,\varphi \right)$ Eq (4) and after some mathematical manipulation Eq (13) reduces to:

$$\begin{array}{c} -\frac{\partial^{2}{\hat{V}}_{d}^{}\left(w,\varphi \right)}{\partial^{2}w}+\frac{\partial^{2}{\hat{V}}_{d}^{}\left(w,\varphi \right)}{\partial^{2}\varphi }=Z_{o}\frac{\partial }{\partial w}\left(\frac{1}{jk\sin w}\frac{\partial }{\partial w}{\hat{J}}_{z}\left(w,\varphi \right)\right)=\hat{S}\left(w,\varphi \right) \end{array}$$ (14)

The procedure to obtain Eq (14) has been inspired by [40, 41], although in [40, 41] no source is present. By applying the Laplace transform Eq (1) to the Maxwell-Faraday Eq (15)

$$\begin{array}{c} -j\rho kZ_{o}H_{\rho }=\frac{\partial E_{z}}{\partial \varphi } \end{array}$$ (15)

and introducing the w plane (*η* = −*k* cos *w*) we have

$$\begin{array}{c} -Z_{o}\frac{\partial {\hat{I}}_{+}\left(w,\varphi \right)}{\partial w}=\frac{\partial {\hat{V}}_{d}\left(w,\varphi \right)}{\partial \varphi } \end{array}$$ (16)

The application of the MF transform Eqs (5) to (16) and to Eq (14) yields:

$$\begin{array}{c} \frac{\partial {\bar{V}}_{d}\left(t,\varphi \right)}{\partial \varphi }=jtZ_{o}{\bar{I}}_{+}\left(t,\varphi \right) \end{array}$$ (17)

$$\begin{array}{c} \frac{\partial^{2}{\bar{V}}_{d}\left(t,\varphi \right)}{\partial \varphi^{2}}+t^{2}{\bar{V}}_{d}\left(t,\varphi \right)=\bar{S}\left(t,\varphi \right) \end{array}$$ (18)

Substituting Eq (17) into Eq (18) yields

$$\begin{array}{c} \frac{\partial {\bar{I}}_{+}\left(t,\varphi \right)}{\partial \varphi }=jtY_{o}{\bar{V}}_{d}\left(t,\varphi \right)+\frac{\bar{S}\left(t,\varphi \right)}{jtZ_{o}} \end{array}$$ (19)

where $\bar{S}\left(t,\varphi \right)$ is the MF transform of the right end side of Eq (14).

Eqs (17) and (19) are classical equations of transmission lines in frequency domain where the azimuthal angle *φ* and the spectral variable t take respectively the role of the direction of propagation and the propagation constant.

In absence of sources $\bar{S}\left(t,\varphi \right)=0$, by applying the inverse MF transform Eqs (6) to (17) and Eq (19) we obtain

$$\begin{array}{c} \begin{array}{c} -Z_{o}\frac{\partial {\hat{I}}_{+}\left(w,\varphi \right)}{\partial w}=\frac{\partial {\hat{V}}_{d}\left(w,\varphi \right)}{\partial \varphi }\\ -Y_{o}\frac{\partial {\hat{V}}_{d}\left(w,\varphi \right)}{\partial w}=\frac{\partial {\hat{I}}_{+}\left(w,\varphi \right)}{\partial \varphi } \end{array} \end{array}$$ (20)

The functions ${\hat{V}}_{d}\left(w,\varphi \right)$ and ${\hat{I}}_{+}\left(w,\varphi \right)$ satisfy the transmission line Eq (20) in time domain where the variable *w* takes the role of the time and the azimuthal angle *φ* takes the role of the direction of propagation [34].

### 4.1 Network model of angular regions in absence of sources

Let us consider an angular region *φ*_1_ < *φ* < *φ*_2_ without sources, the functions ${\bar{V}}_{d}\left(t,\varphi \right)$ and ${\bar{I}}_{+}\left(t,\varphi \right)$ satisfy the transmission line Eqs (17) and (19).

As already stated, taking into account the uniqueness theorem, two equations must relate the four quantities ${\bar{V}}_{qd}\left(t\right),\;{\bar{I}}_{q+}\left(t\right)$ (*q* = 1, 2) in spectral domains (see Eq (2) for definition of q). In *t*-plane the two relations are algebraic and assumes the typical properties of transmission line in frequency domain, therefore the solution of Eqs (17) and (19) in terms of ${\bar{V}}_{qd}\left(t\right),\;{\bar{I}}_{q+}\left(t\right)$ (*q* = 1, 2) in absence of internal sources yields the admittance parameters of the classical two-port network (see Fig 6b) without current generators constituted by a segment of transmission line (see also [3] p.181):

$$\begin{array}{c} \left(\begin{array}{c} {\bar{I}}_{1+}\\ -{\bar{I}}_{2+} \end{array}\right)=\left[\begin{array}{cc} {\bar{Y}}_{11}^{e} & {\bar{Y}}_{12}^{e}\\ {\bar{Y}}_{21}^{e} & {\bar{Y}}_{22}^{e} \end{array}\right]\left(\begin{array}{c} {\bar{V}}_{1d}\\ {\bar{V}}_{2d} \end{array}\right) \end{array}$$ (21)

$$\begin{array}{c} \left[\begin{array}{cc} {\bar{Y}}_{11}^{e} & {\bar{Y}}_{12}^{e}\\ {\bar{Y}}_{21}^{e} & {\bar{Y}}_{22}^{e} \end{array}\right]=\left[\begin{array}{cc} jY_{o}\cot\left(t\;l_{\varphi }\right) & -jY_{o}\frac{1}{\sin(t\;l_{\varphi })}\\ -jY_{o}\frac{1}{\sin(t\;l_{\varphi })} & jY_{o}\cot\left(t\;l_{\varphi }\right) \end{array}\right] \end{array}$$ (22)

where *l*_*φ*_ = *φ*_2_ − *φ*_1_

As in classical circuit theory for reciprocal and symmetrical circuits, in absence of sources, we can model the admittance matrix through a *π* two-port networks (see Fig 8) whose parameters are

$$\begin{array}{c} {\bar{Y}}_{1}^{e}=-jY_{o}\tan\left(t\;\frac{\;l_{\varphi }}{2}\right),\;{\bar{Y}}_{3}^{e}=jY_{o}\frac{1}{\sin(t\;\;l_{\varphi })} \end{array}$$ (23)

Note that in Eq (23) we have reported the admittances in *t*-plane which are algebraic.

The network model reported in Fig 6b without current generators can be reinterpreted in *w* plane. We note that, by applying the inverse MF transform Eq (6) to

$$\begin{array}{c} {\bar{I}}_{+}\left(t\right)={\bar{Y}}_{}\left(t\right){\bar{V}}_{d}\left(t\right) \end{array}$$ (24)

we obtain

$$\begin{array}{c} {\hat{I}}_{+}\left(w\right)=M^{-1}\left\{\bar{Y}\left(t\right){\bar{V}}_{d}\left(t\right)\right\}=\int_{-j\infty }^{+j\infty }\hat{Y}\left(w-w^{\prime }\right){\hat{V}}_{d}\left(w^{\prime }\right)dw^{\prime }=\hat{Y}\left(w\right)*{\hat{V}}_{d}\left(w\right) \end{array}$$ (25)

where the convolution theorem is applied in the framework of the MF transform.

From Eq (25), we notice that the admittances are convolutional integral kernels in the *w*-plane. The inverse MF transforms of Eq (22) yield:

$$\begin{array}{c} {\hat{Y}}_{11}^{e}\left(w\right)={\hat{Y}}_{22}^{e}\left(w\right)=-\frac{1}{2\pi }\int_{-j\infty }^{+j\infty }{\bar{Y}}_{11}\left(t\right)e^{-jtw}dt=j\frac{Y_{o}}{2l_{\varphi }}\cot\left(\frac{\pi }{2l_{\varphi }}w\right) \end{array}$$ (26)

$$\begin{array}{c} {\hat{Y}}_{12}^{e}\left(w\right)={\hat{Y}}_{21}^{e}\left(w\right)=-\frac{1}{2\pi }\int_{-j\infty }^{j\infty }{\bar{Y}}_{12}\left(t\right)e^{-j\;t\;w}dt=j\frac{Y_{o}}{2l_{\varphi }}\tan\left(\frac{\pi }{2l_{\varphi }}\left(w\right)\right) \end{array}$$ (27)

In Eqs (26) and (27) we have reported the admittances in *w*-plane. The model in *w* plane is still a two port network represented by Fig 6b without current generators, although the admittances are now convolutional operators, see Eq (25).

### 4.2 Sources concentrated along an azimuthal direction

Let us consider a homogeneous angular region with a source concentrated along the azimuthal direction *φ*_*o*_, i.e. *J*_*z*_(*ρ*, *φ*) = *J*_*z*_(*ρ*)*δ*(*φ* − *φ*_*o*_). With reference to Fig 4 the region S reduces to a line. With this source constraint Eq (19) can be rewritten as

$$\begin{array}{c} \frac{\partial {\bar{I}}_{+}\left(t,\varphi \right)}{\partial \varphi }=jtY_{o}{\bar{V}}_{d}\left(t,\varphi \right)+{\bar{I}}_{o}\left(t\right)\delta \left(\varphi -\varphi_{o}\right) \end{array}$$ (28)

since the source term $\bar{S}\left(t,\varphi \right)$ in Eq (19) is null for *φ* ≠ *φ*_*o*_.

The *E*_*z*_-polarized plane wave Eq (29) is a particular case, where a point source is located at infinity toward the azimuthal direction *φ*_*o*_.

$$\begin{array}{c} E_{z}^{i}=E_{o}e^{jk\;\rho \cos(\varphi -\varphi_{o}^{})}=E_{o}e^{jk\cos\varphi_{o}\;x+jk\sin\varphi_{o}\;y} \end{array}$$ (29)

In this case, the direct computation of ${\bar{I}}_{o}\left(t\right)$, i.e. $\bar{S}\left(t,\varphi \right)$, through spectral transformations is not possible since we cannot describe in closed form the source *J*_*z*_(*ρ*, *φ*), therefore we need to resort to a different procedure, see Eqs (17), (18) of [34]. Since ${\bar{I}}_{o}\left(t\right)$ is a quantity due to the presence of the incident wave, we compute the current ${\bar{I}}_{+}^{i}\left(t,\varphi \right)$ due to the incident wave through (17b) of [34] by using (18)s of [34] at $\varphi =\varphi_{o}^{\pm }$. The jump of ${\bar{I}}_{+}^{i}\left(t,\varphi \right)$ at *φ* = *φ*_*o*_ gives

$$\begin{array}{c} {\bar{I}}_{o}\left(t\right)={\bar{I}}_{+}^{i}\left(t,\varphi_{o+}\right)-{\bar{I}}_{+}^{i}\left(t,\varphi_{o-}\right)=-4\pi jY_{o}E_{o} \end{array}$$ (30)

The procedure used in [34] is based on the computation of rotating waves for an incident plane wave without any structure/obstacles since $\bar{S}\left(t,\varphi \right)$ is independent from the terminations (i.e the network parameters) of the angular region under consideration.

Since the representation of a plane wave with incident direction *φ*_*o*_ is modelled by a concentrated source ${\bar{I}}_{o}\left(t\right)\delta \left(\varphi -\varphi_{o}\right)$ in *t*, the network model of this kind of source is a current generator of intensity ${\bar{I}}_{o}\left(t\right)$ located at *φ* = *φ*_*o*_.

**Fig 9:**
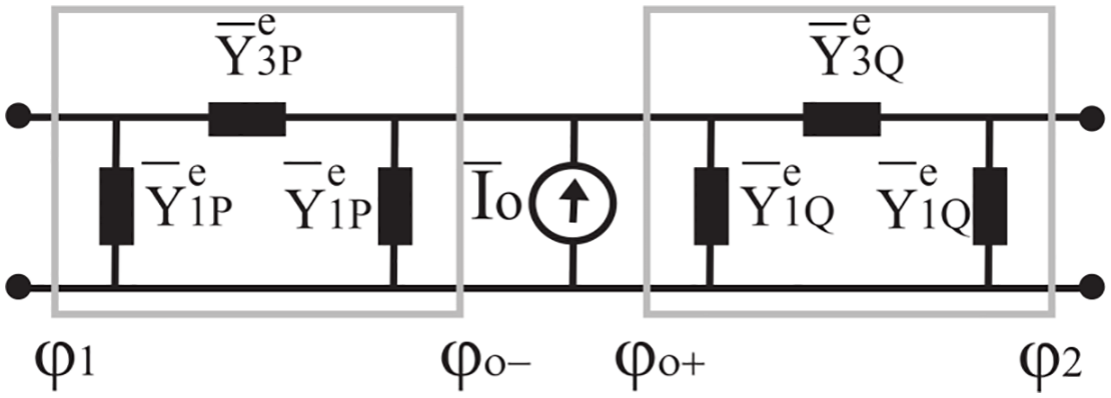
The equivalent network in t plane for an angular region excited by a plane wave.

Taking advantage of *π* network model of the previous subsection, the representation of an angular region *φ*_1_ < *φ* < *φ*_2_ illuminated by a plane wave with incident direction *φ*_1_ < *φ*_*o*_ < *φ*_2_ is constituted of two *π* networks, one for each free-of-source sub angular region P and Q (i.e *φ*_1_ < *φ* < *φ*_*o*_ and *φ*_*o*_ < *φ* < *φ*_2_), with a current generator ${\bar{I}}_{o}\left(t\right)$ located at *φ* = *φ*_*o*_. This network representation in *t* plane is reported in Fig 9.

### 4.3 Norton equivalent currents for a plane wave source

The estimation of the Norton equivalent currents for an angular region illuminated by a plane wave source is obtained through an auxiliary electromagnetic problem.

**Fig 10:**
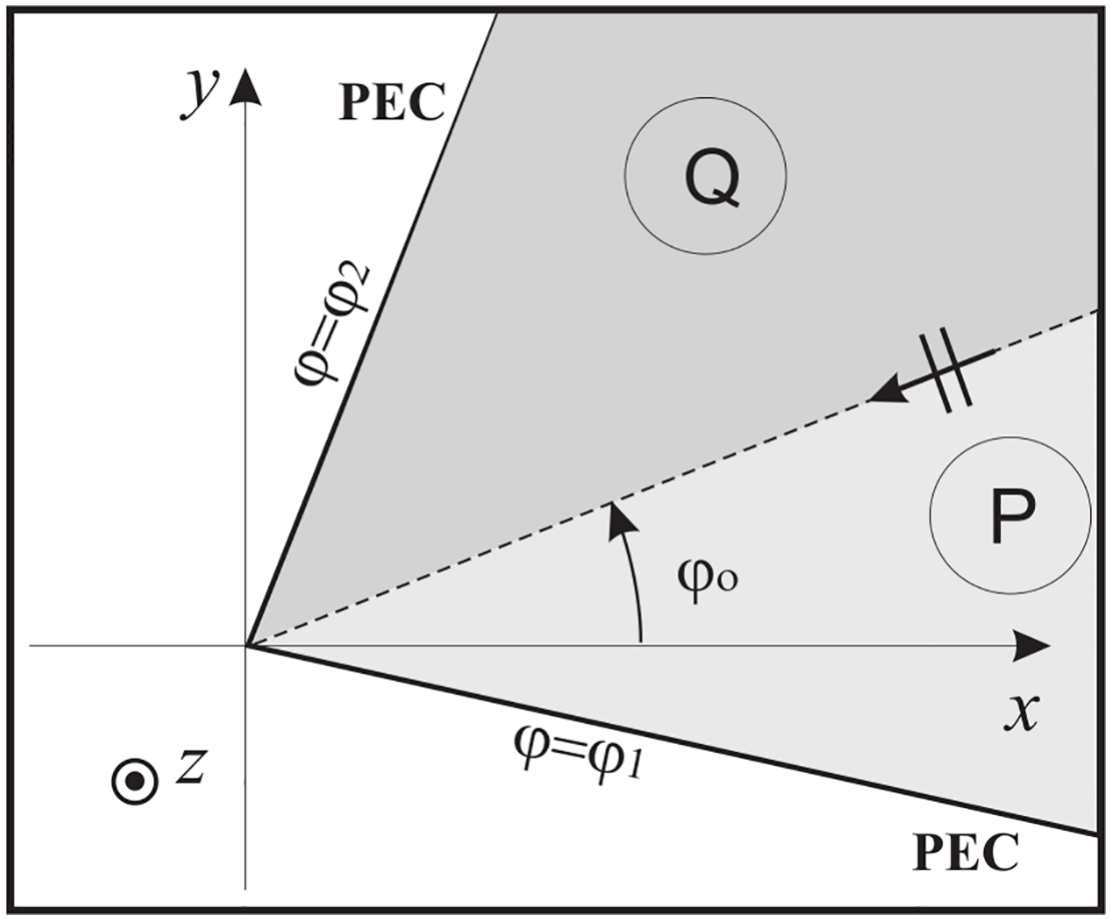
PEC wedge excited by an incident plane wave.

**Fig 11:**
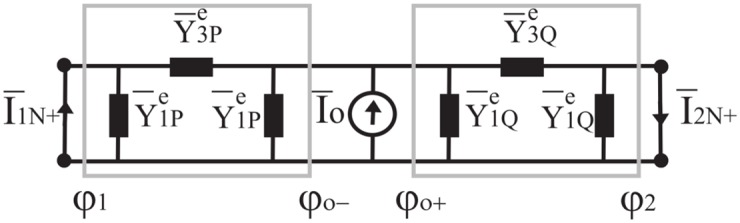
The equivalent network in t plane relevant to the problem described in Fig 10.

Fig 10 presents the problem of a PEC wedge with aperture angle 2*π* − (*φ*_1_ + *φ*_2_) in free space and illuminated by a plane wave at normal incidence with direction *φ*_1_ < *φ*_*o*_ < *φ*_2_ and electric field intensity *E*_*o*_ Eq (29). The free space angular region is subdivided into two homogenous regions P (*φ*_1_ < *φ* < *φ*_*o*_) and Q (*φ*_*o*_ < *φ* < *φ*_2_). This problem allows to compute the Norton equivalent currents. According to the theory presented in the previous Sections, the problem can be modelled using a network in *t*-plane, see Fig 11, terminated by short-circuits. The two *π* networks are constituted of the following admittances:

$$\begin{array}{c} {\bar{Y}}_{1P}^{e}=-jY_{o}\tan\left(t\;\frac{\varphi_{o}-\varphi_{1}}{2}\right),\;{\bar{Y}}_{3P}^{e}=jY_{o}\frac{1}{\sin(t\;\left(\varphi_{o}-\varphi_{1}\right))} \end{array}$$ (31)

$$\begin{array}{c} {\bar{Y}}_{1Q}^{e}=-jY_{o}\tan\left(t\;\frac{\varphi_{2}-\varphi_{o}}{2}\right),\;{\bar{Y}}_{3Q}^{e}=jY_{o}\frac{1}{\sin(t\;\left(\varphi_{2}-\varphi_{o}\right))} \end{array}$$ (32)

By analyzing the network of Fig 11, we obtain the equivalent Norton currents

$$\begin{array}{c} \begin{array}{c} {\bar{I}}_{1N+}=\frac{-{\bar{Y}}_{3P}^{e}{\bar{I}}_{o}}{{\bar{Y}}_{1P}^{e}+{\bar{Y}}_{3P}^{e}+{\bar{Y}}_{1Q}^{e}+{\bar{Y}}_{3Q}^{e}}\\ {\bar{I}}_{2N+}=\frac{{\bar{Y}}_{3Q}^{e}{\bar{I}}_{o}}{{\bar{Y}}_{1P}^{e}+{\bar{Y}}_{3P}^{e}+{\bar{Y}}_{1Q}^{e}+{\bar{Y}}_{3Q}^{e}} \end{array} \end{array}$$ (33)

Taking into account Eqs (21), (22) and (33) we obtain that the Norton model in *t* plane in presence of an incident plane source is

$$\begin{array}{c} \left(\begin{array}{c} {\bar{I}}_{1+}\\ -{\bar{I}}_{2+} \end{array}\right)=\left[\begin{array}{cc} {\bar{Y}}_{11}^{e} & {\bar{Y}}_{12}^{e}\\ {\bar{Y}}_{21}^{e} & {\bar{Y}}_{22}^{e} \end{array}\right]\left(\begin{array}{c} {\bar{V}}_{1d}\\ {\bar{V}}_{2d} \end{array}\right)+\left(\begin{array}{c} {\bar{I}}_{1N+}\\ -{\bar{I}}_{2N+} \end{array}\right) \end{array}$$ (34)

The inverse MF transforms of Eq (33) yield the equivalent Norton currents in *w*-plane Eq (35) and by using the inverse mapping of *η* = −*k* cos(*w*) (see for instance [30]) we obtain the equivalent Norton currents in the *η* plane.

$$\begin{array}{c} \begin{array}{c} {\hat{I}}_{1N+}\left(w\right)=\frac{j\pi Y_{o}}{k\left(\varphi_{2}-\varphi_{1}\right)}\frac{2\sin[\frac{\pi (\varphi_{2}-\varphi_{o})}{\left(\varphi_{2}-\varphi_{1}\right)}]}{\cos\left[\frac{\pi w}{\left(\varphi_{2}-\varphi_{1}\right)}\right]+\cos\left[\frac{\pi (\varphi_{2}-\varphi_{o})}{\left(\varphi_{2}-\varphi_{1}\right)}\right]}\\ {\hat{I}}_{2N+}\left(w\right)=-\frac{j\pi Y_{o}}{k\left(\varphi_{2}-\varphi_{1}\right)}\frac{2\sin[\frac{\pi (\varphi_{2}-\varphi_{o})}{\left(\varphi_{2}-\varphi_{1}\right)}]}{\cos\left[\frac{\pi w}{\left(\varphi_{2}-\varphi_{1}\right)}\right]-\cos\left[\frac{\pi (\varphi_{2}-\varphi_{o})}{\left(\varphi_{2}-\varphi_{1}\right)}\right]} \end{array} \end{array}$$ (35)

The expressions reported in Eq (35) of ${\hat{I}}_{1N+}\left(w\right),{\hat{I}}_{2N+}\left(w\right)$ are compared to the ones obtained in the PEC wedge problem illuminated by a plane wave by using the method of rotating waves [34]. Eqs (21)–(23) and (31)–(33) define the complete Norton representation Eq (34) in *t* plane through algebraic equations, while via the inverse MF transform Eqs (25)–(27) and (35) define the Norton model in w plane Eq (36) through integral operators.

$$\begin{array}{c} \left(\begin{array}{c} {\hat{I}}_{1+}\\ -{\hat{I}}_{2+} \end{array}\right)=\left[\begin{array}{cc} {\hat{Y}}_{11}^{e} & {\hat{Y}}_{12}^{e}\\ {\hat{Y}}_{21}^{e} & {\hat{Y}}_{22}^{e} \end{array}\right]*\left(\begin{array}{c} {\hat{V}}_{1d}\\ {\hat{V}}_{2d} \end{array}\right)+\left(\begin{array}{c} {\hat{I}}_{1N+}\\ -{\hat{I}}_{2N+} \end{array}\right) \end{array}$$ (36)

The Norton network model Eqs (34) and (36) of the angular region *φ*_1_ < *φ* < *φ*_2_ illuminated by an incident plane wave is Fig 6b.

## 5 Norton representation of a terminated angular region: The one-port representation

Complex wedge problems are constituted by penetrable and impenetrable angular regions that can be located over a layered region. Connected angular regions with different material parameters are modelled through two-port networks in cascade following the sequence of the physical problem. Although the topology of the network is simple, we need to pay attention to the spectral domain in use in each angular region since the *w* planes are defined in terms of the propagation constant *k* of each homogenous angular regions (*η* = −*k* cos *w*). A sophisticated technique to link w planes related to different media is effectively proposed in [31].

Frequently, penetrable and impenetrable angular regions can be modelled by surface impedances [16]. Let us consider again the *E*_*z*_-polarization. The surface impedance model relates the voltage and current through algebraic relations in *η* and *w* planes Eq (37) that yield a convolutional relation in *t* plane due to the MF transform, therefore in general it is less cumbersome to deal with surface impedance terminations in *η* and *w* planes.

$$\begin{array}{c} \begin{array}{c} V_{q+}\left(\eta \right)=Z_{q}^{}I_{q+}\left(\eta \right)\\ {\hat{V}}_{qd}\left(w\right)=\frac{Z_{q}^{}}{\sin w}{\hat{I}}_{q+}\left(w\right) \end{array},\;q=1,2 \end{array}$$ (37)

Perfect electric conducting (PEC) surface and perfect magnetic conducting (PMC) surface show terminations that are easy to be implemented in any spectral domain, i.e. respectively *Z*_*q*_ = 0 and *Z*_*q*_ = +∞.

When an angular region is delimited by an impenetrable surface, the circuit model is a two-port network with a termination in one port that reduces the representation to a one port network. We note that in general the network representations in different complex spectral planes (*t*, *w*, *η*) can be obtained starting from *t* plane to *η* plane or viceversa. The selection of the appropriate spectral plane depends on the problem under investigation: the physical parameters, the geometry, the terminations…

When the angular region is terminated by a PEC, one port of the two-port network is short-circuited (*Z*_*q*_ = 0) at *E*_*z*_ polarization. In this case the constitutive constraint is ${\tilde{V}}_{+}={\tilde{V}}_{d}=0$ in any spectral plane, thus the *t* plane is the most simple to enforce the condition due to the algebraic nature of the admittance. Throughout the paper we focus the implementation on problems constituted of PEC wedge over a penetrable/impenetrable layer with *E*_*z*_-polarization. The next subsection is about the PEC termination of an angular region.

### 5.1 Angular region terminated by a PEC surface and illuminated by a plane wave

**Fig 12:**
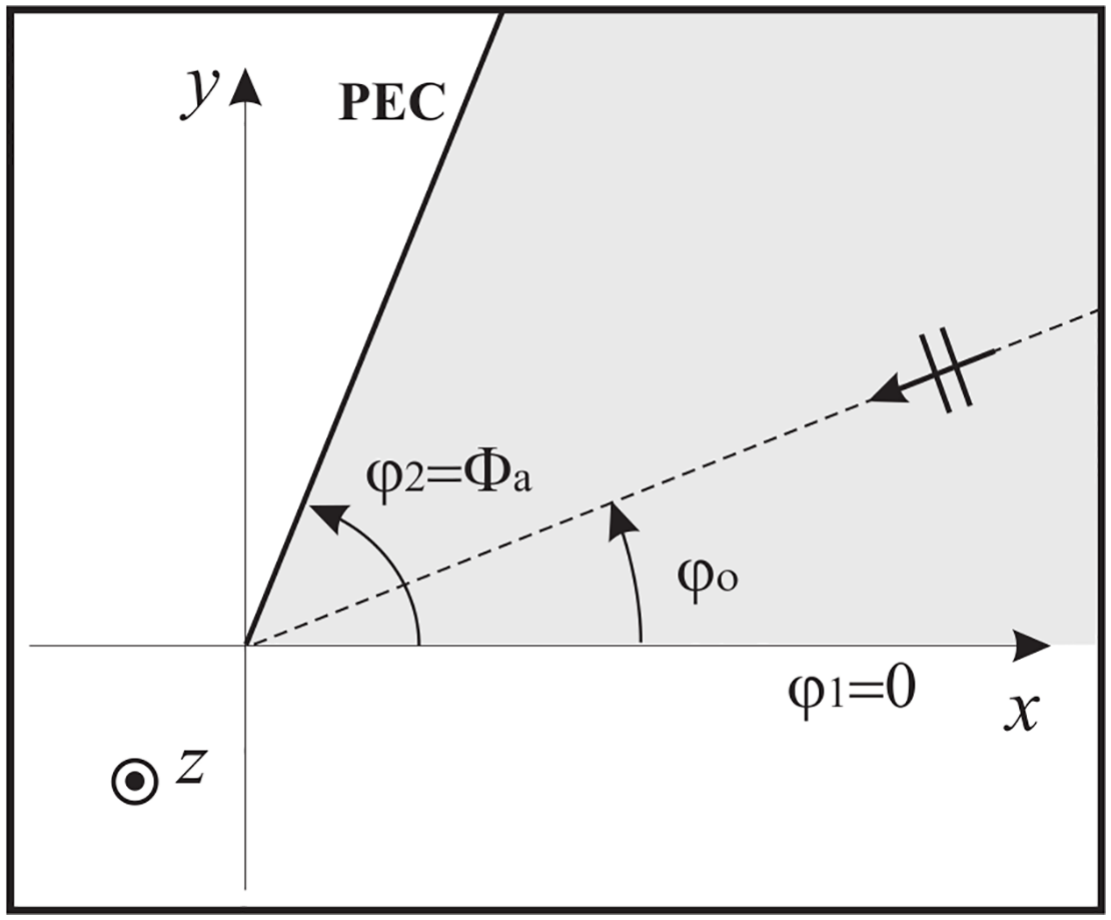
Angular region delimited by a PEC half-plane.

Without loss of generality, let us suppose to a particular problem where the angular region 0 = *φ*_1_ < *φ* < *φ*_2_ = Φ_*a*_ is terminated by a PEC half-plane at Φ_*a*_ and illuminated by an *E*_*z*_-polarized plane wave with incident angle *φ*_*o*_, see Fig 12.

With reference to Fig 6b, in *t* plane taking into account Eq (34), we get the one port Norton representation

$$\begin{array}{c} {\bar{I}}_{1+}\left(t\right)={\bar{Y}}_{11}^{e}\left(t\right){\bar{V}}_{1d}\left(t\right)+{\bar{I}}_{1N+}\left(t\right) \end{array}$$ (38)

since ${\bar{V}}_{2d}=0$.

In *w* plane Eq (38) becomes through Eqs (6), (7) and (25)

$$\begin{array}{c} {\hat{I}}_{1+}\left(w\right)={\hat{Y}}_{11}^{e}\left(w\right)*{\hat{V}}_{1d}\left(w\right)+{\hat{I}}_{1N+}\left(w\right) \end{array}$$ (39)

**Fig 13:**
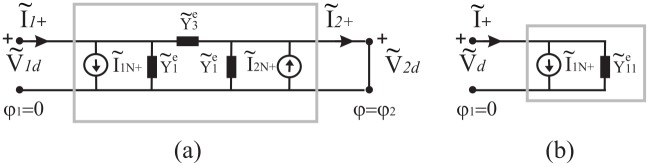
Network models of the problem describe in Fig 12.

By using Eqs (26) and (35) the explicit form of Eq (39) is

$$\begin{array}{c} {\hat{I}}_{1+}\left(w\right)=\frac{jY_{o}}{2\Phi_{a}}P.V.\int_{-j\infty }^{+j\infty }\cot\left(\frac{\pi }{2\Phi_{a}}\left(w-w^{\prime }\right)\right){\hat{V}}_{1d}\left(w^{\prime }\right)dw^{\prime }+\frac{j\pi Y_{o}}{k\Phi_{a}}\frac{2\sin(\frac{\pi }{\Phi_{a}}\varphi_{o})}{\cos\left(\frac{\pi }{\Phi_{a}}w\right)-\cos\left(\frac{\pi }{\Phi_{a}}\varphi_{o}\right)} \end{array}$$ (40)

that holds for *Re*[*w*] = 0 and PV stands for Principal Value due to the definition of MF transform. The network representation of Eqs (38) and (40) is reported in Fig 13 although the admittance are algebraic quantities in *t* plane and convolutional operators in *w* plane.

We remark that Eq (40) holds independently from the media or sources located outside of the angular region under investigation, i.e. 0 < *φ* < Φ_*a*_. Moreover, the validity of Eq (40) has been ascertained using a completely different procedure in *η* = −*k* cos *w* plane starting from the GWH formulation via Fredholm factorization [32, 35], see Section 7.1 for a short description.

**Fig 14:**
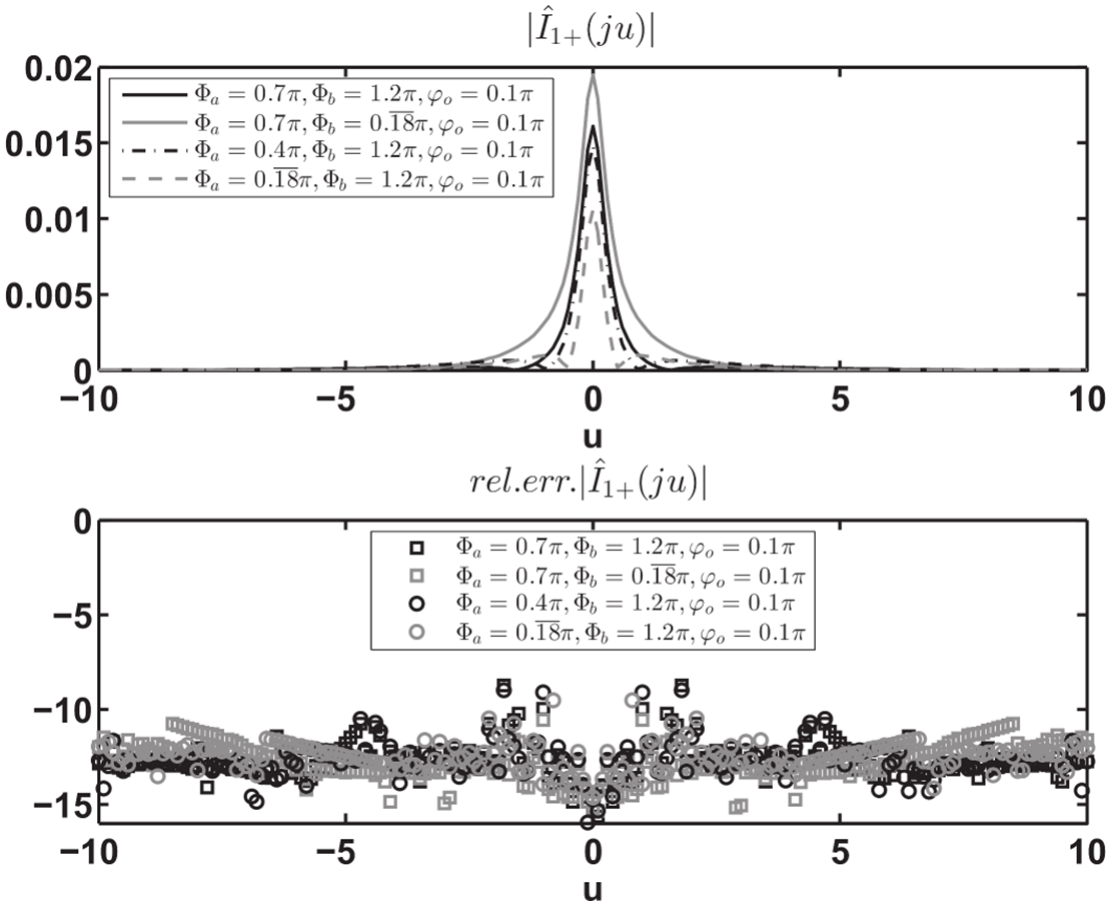
Validation of Eq (40). Absolute value of ${\hat{I}}_{1+}\left(ju\right)$ on top and relative error in log_10_ scale on bottom obtained from the approximate expression of ${\hat{I}}_{1+}\left(ju\right)$ given by Eq (40) while substituting the exact expression of ${\hat{V}}_{1d}\left(w\right)$ and performing the numerical PV integration. Test cases are relative to different sets of parameters [Φ_*a*_, Φ_*b*_, *φ*_*o*_], where *φ* = Φ_*a*_ and *φ* = −Φ_*b*_ are the azimuthal directions of the PEC faces of the wedge and *φ* = *φ*_*o*_ is the azimuthal direction of the incident *E*_*z*_-polarized plane wave.

Moreover, the numerical verification of Eq (40) has been performed for the auxiliary problem constituted by a PEC wedge with PEC faces located at *φ* = Φ_*a*_ and *φ* = −Φ_*b*_ for several values of Φ_*a*_, Φ_*b*_, *φ*_*o*_. In particular we have considered the angular region defined by 0 = *φ*_1_ < *φ* < *φ*_2_ = Φ_*a*_ to test Eq (40). The comparison has been done with respect to the exact solutions of the PEC wedge problem in terms of ${\hat{V}}_{1+}\left(w\right)={\hat{V}}_{1d}\left(w\right)/\sin w$ and ${\hat{I}}_{1+}\left(w\right)$, see (73) and (74) of [8]. We compute the relative error of the approximated value of ${\hat{I}}_{1+}\left(w\right)$ for *Re*[*w*] = 0, i.e. *w* = *ju* with u∈R, obtained via numerical integration of Eq (40) when substituting the exact expression of ${\hat{V}}_{1d}\left(w\right)$ and taking into account specialized quadrature techniques to handle the PV integration as [42]. Fig 14 reports on top the absolute value of ${\hat{I}}_{1+}\left(ju\right)$, on the bottom the relative error in log_10_ scale obtained from the approximate expression of ${\hat{I}}_{1+}\left(ju\right)$. The different curves refer to different sets of parameters Φ_*a*_, Φ_*b*_, *φ*_*o*_.

In particular Fig 14b shows that the relative error is scattered and is approximately around 10^−15^ ÷ 10^−9^. The different performances are due to the numerical implementation of the PV integration in Eq (40).

## 6 From network modelling to Fredholm integral equations of second kind

The aim of this paper is to formulate complex problems as the ones reported in Fig 3 through electrical networks whose components are multiport described by circuital representations. In particular, in the previous Sections we have obtained the Norton representation of a homogeneous angular region. Unfortunately, this representation Eq (40) contains singular kernel, thus integral equations obtained through the proposed network model are singular and specialized quadrature technique are required for their numerical evaluation. In general, it is amenable to obtain integral equations with compact non singular kernels and in particular to resort to Fredholm integral equations of second kind (FIEs). With compact kernels, Fredholm theory guarantees the convergence via numerical procedure of integral equations of second kind [43]. Other possible procedures to guarantee the convergence of FIEs under examination have been taken into account, for example through the definition suitable space of functions as done in [15], through suitable mappings to reduce the infinite integration interval to a finite one with optimal functional properties of the integrand in [47] or truncating the integration interval due to the behaviour of the spectral unknowns [43].

To get FIEs for the problems of Fig 3, we modify the above Norton representations in order to obtain compact not singular kernels.

The procedure to obtain FIEs in *η* plane with compact non-singular kernel is general and consists of the following steps:

1. Contour indentation to remove the PV integration in w plane and to extend the validity of integral equations in *w* plane
2. Modification of integral kernel by adding and subtracting terms to obtain a well-behaved kernel in *w* plane at *w*′ = +*w*
3. Contour deformation to take advantage of compact kernel and enhance convergence of integral equations with the extraction of structural and non-structural singularities in *w* plane
4. Handling the integral equations in modified *w* plane and *η* plane to get FIEs for practical problems
5. Analytic extension of the integral representation in the whole complex plane *η* including the improper sheet

Just to illustrate the methodology, we refer again to a particular problem: the angular region (0 < *φ* < Φ_*a*_) terminated by a PEC face at Φ_*a*_ with an *E*_*z*_-polarized incident plane wave, see Eq (40). For the reader’s convenience we anticipate that the final equation in *η* plane is Eq (72) provided the observation point η∈R.

The above procedure (summarized in steps) allows to substitute in Eq (40) the singular kernel with a compact one. The drawback will be that in the new representation with compact kernel the Norton current sources will depend also from the GO field that is present outside of the angular region under investigation. It means that to get the improved Norton representation, we must evaluate the GO field in the whole geometry where the angular region is one of the sub-domains. As an example, in the following, the considered entire problem is the scattering by a wedge with PEC faces at Φ_*a*_ and −Φ_*b*_, i.e. aperture angle −Φ_*b*_ < *φ* < Φ_*a*_, with an *E*_*z*_ polarized incident plane wave.

Step 4 allows to handle the angular region problem either in plane *w* and plane *η* via FIEs. The procedure is systematic for angular regions with obtuse aperture angles, while it needs particular attention to deal with acute aperture angles. In this last case we anticipate that structural singularities (better *kernel singularity lines*) due to the Kernel’s shape and the contour deformation yield new integral terms and non-integral terms in FIEs with respect to the FIEs formulated for obtuse aperture angles (see next subsections for details).

We have identified three ranges of aperture angles with different mathematical properties: obtuse *π*/2 < Φ_*a*_ < *π*, acute *π*/4 < Φ_*a*_ < *π*/2, *hyper-acute* 0 < Φ_*a*_ < *π*/4. In the following we will make references to relevant test cases which illustrate the mathematical properties of the three ranges described above. In particular we will consider the entire wedge problem with $\Phi_{a}=0.7\pi ,0.4\pi ,0.\bar{18}\pi \left(i.e.\;\pi /5.5\right)$, $\Phi_{b}=0.\bar{18}\pi ,1.2\pi$, *φ*_*o*_ = 0.1*π*. The reference solution is the exact spectra of the wedge problem reported in (73) and (74) of [8].

### 6.1 Step 1

Since Eq (40) holds for *Re*[*w*] = 0 from the definition of inverse MF transform Eq (6), first, we want to extend the formulation for *Re*[*w*] < 0. We observe that the kernel Eq (41) in Eq (40) has *kernel singularities* located at *w*′ = *w* + 2*nΦ*_*a*_ (n∈Z) that constitute *lines of singularities* (for any complex w we have the singularity located at *w*′ = *w* + 2*nΦ*_*a*_). Limiting the extension to the strip −2Φ_*a*_ < *Re*[*w*] < 0, only *w*′ = *w* gives contribution: for any *w* we have the pole *w*′.

$$\begin{array}{c} K_{o}\left(w,w^{\prime }\right)=\cot\left(\frac{\pi }{2\Phi_{a}}\left(w-w^{\prime }\right)\right)=\frac{\sin\frac{\pi }{\Phi_{a}}w^{\prime }}{\cos\frac{\pi }{\Phi_{a}}w^{\prime }-\cos\frac{\pi }{\Phi_{a}}w}+\frac{\sin\frac{\pi }{\Phi_{a}}w}{\cos\frac{\pi }{\Phi_{a}}w^{\prime }-\cos\frac{\pi }{\Phi_{a}}w} \end{array}$$ (41)

Let us consider the indented contour *γ*, which is the vertical line *Re*[*w*′] = 0 indented near *w*′ = *w* through a small semi-circumference on the right side of the imaginary axis. Taking into account the integral part of Eq (40), by deforming the contour integration line from M (*Re*[*w*′] = 0) to *γ* we obtain

$$\begin{array}{c} P.V.\int_{-j\infty }^{+j\infty }\cot\left(\frac{\pi (w-w^{\prime })}{2\Phi_{a}}\right){\hat{V}}_{1d}\left(w^{\prime }\right)dw^{\prime }=\int_{\gamma }\cot\left(\frac{\pi (w-w^{\prime })}{2\Phi_{a}}\right){\hat{V}}_{1d}\left(w^{\prime }\right)dw^{\prime }-\pi j{\text{R}[\cot\left(\frac{\pi (w-w^{\prime })}{2\Phi_{a}}\right){\hat{V}}_{1d}\left(w^{\prime }\right)]|}_{w^{\prime }=w} \end{array}$$ (42)

The term out of the integral is related through the factor 1/2 to the residue due to the pole *w*′ = *w* of $\cot(\frac{\pi (w-w^{\prime })}{2\Phi_{a}})$ and it yields

$$\begin{array}{c} {\text{R}[\cot\left(\frac{\pi }{2\Phi_{a}}\left(w-w^{\prime }\right)\right){\hat{V}}_{1d}\left(w^{\prime }\right)]|}_{w^{\prime }=w}=-\frac{2\Phi_{a}}{\pi }{\hat{V}}_{1d}\left(w\right) \end{array}$$ (43)

Substituting Eq (42) into Eq (40) with Eq (43) we obtain

$$\begin{array}{c} {\hat{I}}_{1+}\left(w\right)=-Y_{o}{\hat{V}}_{1d}\left(w\right)+\frac{jY_{o}}{2\Phi_{a}}\int_{-j\infty }^{+j\infty }\cot\left(\frac{\pi (w-w^{\prime })}{2\Phi_{a}}\right){\hat{V}}_{1d}\left(w^{\prime }\right)dw^{\prime }+\frac{j\pi Y_{o}}{k\Phi_{a}}\frac{2\sin(\frac{\pi }{\Phi_{a}}\varphi_{o})}{\cos\left(\frac{\pi w}{\Phi_{a}}\right)-\cos\left(\frac{\pi \varphi_{o}}{\Phi_{a}}\right)} \end{array}$$ (44)

that holds for −2Φ_*a*_ < *Re*[*w*] < 0. Similarly, by modifying the side of indentation of *γ*, we obtain:

$$\begin{array}{c} {\hat{I}}_{1+}\left(w\right)=Y_{o}{\hat{V}}_{1d}\left(w\right)+\frac{jY_{o}}{2\Phi_{a}}\int_{-j\infty }^{+j\infty }\cot\left(\frac{\pi (w-w^{\prime })}{2\Phi_{a}}\right){\hat{V}}_{1d}\left(w^{\prime }\right)dw^{\prime }+\frac{j\pi Y_{o}}{k\Phi_{a}}\frac{2\sin(\frac{\pi }{\Phi_{a}}\varphi_{o})}{\cos\left(\frac{\pi w}{\Phi_{a}}\right)-\cos\left(\frac{\pi \varphi_{o}}{\Phi_{a}}\right)} \end{array}$$ (45)

that holds for 0 < *Re*[*w*] < 2Φ_*a*_.

To extend Eqs (44) and (45) respectively in the half planes *Re*[*w*] < 0 and *Re*[*w*] > 0, we need to consider the entire set of *kernel singularities* *w*′ = *w* + 2*nΦ*_*a*_ ($n\in Z_{0}$). In particular Eq (44) is generalized to the half planes *Re*[*w*] < 0 by adding on the right side of the equation the following term

$$\begin{array}{c} -2Y_{o}\sum_{n\in N_{0}}{\hat{V}}_{1d}\left(w+2n\Phi_{a}\right)u\left(-Re\left[w\right]-2n\Phi_{a}\right) \end{array}$$ (46)

where *u*(⋅) is the unit step function, due to the kernel singularities that cross and are captured by the integration line M (*Re*[*w*′] = 0) while moving *w* towards negative real values (note $n\in N_{0}$ in Eq (46)).

We observe that the kernel singularities generate barriers in the analytic extension of the models in *w* plane, see Eqs (44) and (45), and we refer to this effect with the key term *kernel singularity lines* since in general *w* and *w*′ span their entire complex planes and not just real values.

To extend Eq (45) in the half planes *Re*[*w*] > 0 the additional term is

$$\begin{array}{c} +2Y_{o}\sum_{n\in N_{0}}{\hat{V}}_{1d}\left(w-2n\Phi_{a}\right)u\left(Re\left[w\right]-2n\Phi_{a}\right) \end{array}$$ (47)

As already done for Eq (40) in the previous Section, Eqs (44) and (45) have been numerically verified by considering the exact solutions of the PEC wedge problem in terms of ${\hat{V}}_{1+}\left(w\right)={\hat{V}}_{1d}\left(w\right)/\sin w$ and ${\hat{I}}_{1+}\left(w\right)$, see (73) and (74) of [8]. Both Eqs (44) and (45) are integral equations that relate ${\hat{V}}_{1d}\left(w\right)$ to ${\hat{I}}_{1+}\left(w\right)$ through the kernel Eq (41).

To test Eq (44) and to show the importance of Eq (46) we use the auxiliary problem constituted by a PEC wedge with PEC faces located at *φ* = Φ_*a*_ and *φ* = −Φ_*b*_ for several values of Φ_*a*_, Φ_*b*_, *φ*_*o*_. To implement Eq (44) we refer to the angular region 0 = *φ*_1_ < *φ* < *φ*_2_ = Φ_*a*_.

**Fig 15:**
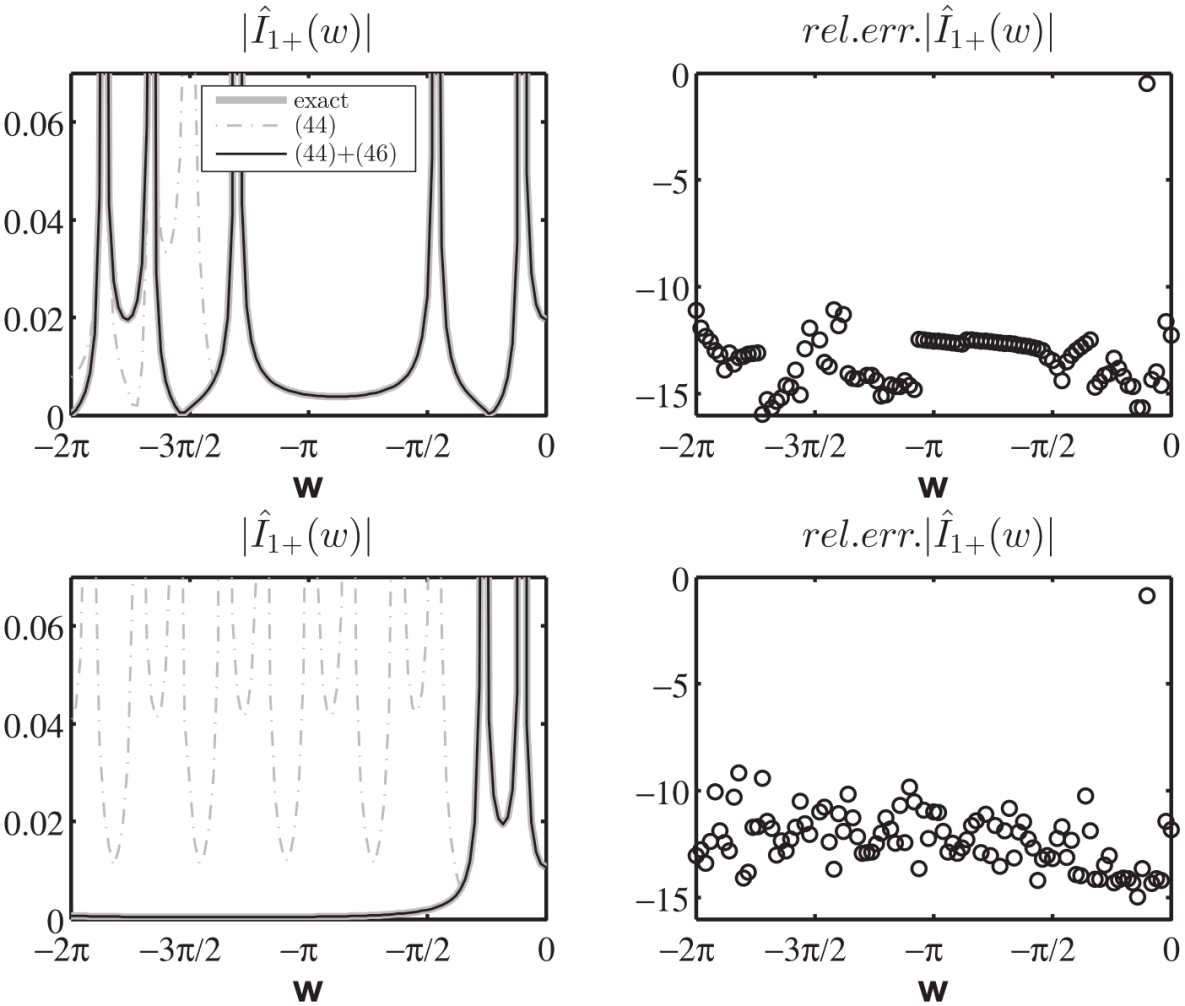
Validation of Eq (44) with the additional term Eq (46). Left: absolute value of ${\hat{I}}_{1+}\left(w\right)$ for w∈R, the exact value (solid thick gray line), the approximate value obtained through Eq (44) (gray dash-dot thin line) and the approximate value obtained by adding Eq (46) to Eq (44) (solid thin black line) while substituting the exact expression of ${\hat{V}}_{1d}\left(w\right)$ and performing the numerical integration. Right: relative error in log_10_ scale obtained from the approximate expression of ${\hat{I}}_{1+}\left(w\right)$ given by Eq (44) with Eq (46). Test cases are relative to different sets of parameters: on top $\left[\Phi_{a},\Phi_{b},\varphi_{o}\right]=\left[0.7\pi ,0.\bar{18}\pi ,0.1\pi \right]$, on bottom $\left[\Phi_{a},\Phi_{b},\varphi_{o}\right]=\left[0.\bar{18}\pi ,1.2\pi ,0.1\pi \right]$. We recall that *φ* = Φ_*a*_ and *φ* = −Φ_*b*_ are the azimuthal directions of the PEC faces of the wedge and *φ* = *φ*_*o*_ is the azimuthal direction of the incident *E*_*z*_-polarized plane wave.

Fig 15 reports on the left the absolute value of ${\hat{I}}_{1+}\left(w\right)$ for w∈R, −2*π* < *w* < 0, obtained from the exact expression (74) of [8] and the numerically estimated expression Eq (44) with-and-without Eq (46) while substituting the exact expression of ${\hat{V}}_{1d}\left(w\right)$ (73) of [8] and performing the numerical integration. The right side of the figure reports the relative error in log_10_ scale obtained from the approximate expression of ${\hat{I}}_{1+}\left(w\right)$ given by Eq (44) with Eq (46).

From Fig 15 we note that the approximate ${\hat{I}}_{1+}\left(w\right)$ is valid for any real *w* < 0 and the relative errors are scattered and are approximately around 10^−16^ ÷ 10^−11^. Eq (45) together with Eq (47) shows similar properties.

From here on, we define the line *M* as the vertical integration line *Re*[*w*] = 0, the line *M*_1_ the vertical integration line *Re*[*w*] = −*π*/2 and the line ${\bar{M}}_{1}$ the vertical integration line *Re*[*w*] = −Φ_*a*_/2. We note that the line *M*_1_ is a line that corresponds to the imaginary axis of *η* if *k* is real.

We observe that Eqs (44) and (45) are integral formulations with a singular non-compact kernels, that cannot be easily estimated using simple quadrature techniques: in particular we cannot numerically evaluate these equations while *w* belongs to *M* (due to the kernel singularity at *w*′ = *w*).

We state that Eqs (44) and (45) respectively with Eqs (46) and (47) are Norton representations Eq (39) with a convolutional operator admittance and a source generator in *w* plane that can be graphically represented as in Fig 13b.

From a mathematical point of view, Eqs (44) and (45) respectively with Eqs (46) and (47) are expressions of ${\hat{I}}_{1+}\left(w\right)$ that constitute an analytic function, while Eqs (44) and (45) by themselves constitute sections of the analytic function ${\hat{I}}_{1+}\left(w\right)$, i.e. Eqs (44) and (45) are *sectionally* analytic functions [44].

### 6.2 Step 2

The procedure to obtain a well-behaved kernel *K*_*new*_(*w*, *w*′) for Eqs (44) and (45) consists of a mathematical artifice. In the following we make reference to the implementation for Eq (44); the procedure to handle Eq (45) is straightforward.

The original kernel *K*_*o*_(*w*, *w*′) Eq (41) is modified by adding a null term *K*_1_(*w*, *w*′) Eq (48), that improves the mathematical behavior of the integral equation.

$$\begin{array}{c} K_{1}\left(w,w^{\prime }\right)=\frac{\Phi_{a}}{\pi }\frac{\sin w}{\cos w^{\prime }-\cos w}-\frac{\Phi_{a}}{\pi }\frac{\sin w}{\cos w^{\prime }-\cos w}=0 \end{array}$$ (48)

Because of the symmetry of the integrand in Eq (49), i.e. the integrand is an odd function of *w*′ (see Section 3), we obtain that

$$\begin{array}{c} \int_{-j\infty }^{j\infty }\left(\frac{\sin\frac{\pi }{\Phi_{a}}w}{\cos\frac{\pi }{\Phi_{a}}w^{\prime }-\cos\frac{\pi }{\Phi_{a}}w}+\frac{\Phi_{a}}{\pi }\frac{\sin w}{\cos w^{\prime }-\cos w}\right){\hat{V}}_{1d}\left(w^{\prime }\right)dw^{\prime }=0 \end{array}$$ (49)

for −2Φ_*a*_ < *Re*[*w*] < 0.

By considering Eqs (41) and (49) the integral term Eq (50) in Eq (44)

$$\begin{array}{c} I=\frac{jY_{o}}{2\Phi_{a}}\int_{-j\infty }^{j\infty }\cot\left(\frac{\pi }{2\Phi_{a}}\left(w-w^{\prime }\right)\right){\hat{V}}_{1d}\left(w^{\prime }\right)dw^{\prime } \end{array}$$ (50)

results to be

$$\begin{array}{c} I=-\frac{Y_{o}}{2\pi j}\int_{-j\infty }^{j\infty }\left(\frac{\frac{\pi }{\Phi_{a}}\sin\frac{\pi }{\Phi_{a}}w^{\prime }}{\cos\frac{\pi }{\Phi_{a}}w^{\prime }-\cos\frac{\pi }{\Phi_{a}}w}-\frac{\sin w}{\cos w^{\prime }-\cos w}\right){\hat{V}}_{1d}\left(w^{\prime }\right)dw^{\prime } \end{array}$$ (51)

for −2Φ_*a*_ < *Re*[*w*] < 0.

Eq (44) can now be rewritten using Eq (51) as

$$\begin{array}{c} {\hat{I}}_{1+}\left(w\right)=-Y_{o}{\hat{V}}_{1d}\left(w\right)-\frac{Y_{o}}{2\pi j}\int_{-j\infty }^{j\infty }\left(\frac{\frac{\pi }{\Phi_{a}}\sin\frac{\pi }{\Phi_{a}}w^{\prime }}{\cos\frac{\pi w^{\prime }}{\Phi_{a}}-\cos\frac{\pi w}{\Phi_{a}}}-\frac{\sin w}{\cos w^{\prime }-\cos w}\right){\hat{V}}_{1d}\left(w^{\prime }\right)dw^{\prime }+\frac{j\pi Y_{o}}{k\Phi_{a}}\frac{2\sin(\frac{\pi }{\Phi_{a}}\varphi_{o})}{\cos\left(\frac{\pi w}{\Phi_{a}}\right)-\cos\left(\frac{\pi \varphi_{o}}{\Phi_{a}}\right)} \end{array}$$ (52)

that holds for −2Φ_*a*_ < *Re*[*w*] < 0 and where the new kernel is

$$\begin{array}{c} K_{new}\left(w,w^{\prime }\right)=\frac{\sin\frac{\pi }{\Phi_{a}}w^{\prime }}{\cos\frac{\pi }{\Phi_{a}}w^{\prime }-\cos\frac{\pi }{\Phi_{a}}w}-\frac{\frac{\Phi_{a}}{\pi }\sin w}{\cos w^{\prime }-\cos w} \end{array}$$ (53)

The new kernel shows kernel singularity lines located at *w*′ = ±(*w* + 2*nΦ*_*a*_) and *w*′ = ±(*w* + 2*nπ*) (n∈Z). While extending Eq (52) for any *Re*[*w*] < 0 we need to take care of these singularity lines (except the one for *n* = 0 already taken into account in Step 1). Moreover, since we have added the null term (null in −2Φ_*a*_ < *Re*[*w*] < 0), we have also to consider its extension for any *w*, in particular such that *Re*[*w*] < 0, taking care of its kernel singularity lines that are the same of the new kernel.

We observe that: 1) the poles *w*′ = ±(*w* + 2*nπ*) yield no contributions for any *Re*[*w*] < 0 because they are canceled by the contributions that comes from the extension of Eq (49) for any *Re*[*w*] < 0; 2) the poles *w*′ = ±(*w* + 2*nΦ*_*a*_) yield contributions that, together with the ones that comes from the extension of Eq (49) for any *Re*[*w*] < 0, give Eq (46) as already studied for the extension of Eq (44). Moreover, from a different point of view, the correction terms Eq (46) is the contribution of the poles *w*′ = ±(*w* + 2*nΦ*_*a*_) reorganized using the symmetry of kernel, the symmetry of ${\hat{V}}_{1d}$ and the crossing direction of the line M performed by the kernel singularities. With the same properties we demonstrate that *w*′ = ±(*w* + 2*nπ*) do not give contributions.

The advantages of Eq (52) with respect to Eq (44) is that the new kernel Eq (53) is regular at *w*′ = *w*, although singular at *w*′ = −*w*. This singularity (*w*′ = −*w*) does not appear along *M*_1_ (i.e *Re*[*w*] = −*π*/2) and thus the new kernel is compact if the line of integration M is warped into *M*_1_. Moreover with integration contour *M*_1_ we obtain quick convergence in the numerical estimation of the integral representation (see Step 3) due to its new kernel Eq (53).

To validate Eq (52) with Eq (46) we have repeated the same tests of validation already done for Eq (44) in step 1, by obtaining similar results as the one reported in Fig 15 for Eq (44) that we omit.

Similarly we can rewrite Eq (45) as

$$\begin{array}{c} {\hat{I}}_{1+}\left(w\right)=+Y_{o}{\hat{V}}_{1d}\left(w\right)-\frac{Y_{o}}{2\pi j}\int_{-j\infty }^{j\infty }\left(\frac{\frac{\pi }{\Phi_{a}}\sin\frac{\pi }{\Phi_{a}}w^{\prime }}{\cos\frac{\pi w^{\prime }}{\Phi_{a}}-\cos\frac{\pi w}{\Phi_{a}}}-\frac{\sin w}{\cos w^{\prime }-\cos w}\right){\hat{V}}_{1d}\left(w^{\prime }\right)dw^{\prime }+\frac{j\pi Y_{o}}{k\Phi_{a}}\frac{2\sin(\frac{\pi }{\Phi_{a}}\varphi_{o})}{\cos\left(\frac{\pi w}{\Phi_{a}}\right)-\cos\left(\frac{\pi \varphi_{o}}{\Phi_{a}}\right)} \end{array}$$ (54)

that holds for *Re*[*w*] > 0 by adding Eq (47).

As already discussed in Step 1 the additional terms Eqs (46) and (47) take into account the complete set of kernel singularity lines *w*′ = ±(*w* + 2*nΦ*_*a*_) (n∈Z).

Again, from a mathematical point of view, Eqs (52) and (54) respectively with Eqs (46) and (47) are expressions of ${\hat{I}}_{1+}\left(w\right)$ that constitute an analytic function, while Eqs (52) and (54) by themselves constitute sections of the analytic function ${\hat{I}}_{1+}\left(w\right)$ [44].

### 6.3 Step 3

The convergence properties of the new kernel Eq (53) along *M*_1_ (*Re*[*w*′] = −*π*/2) suggest to warp the integration contour *M* (*Re*[*w*′] = 0) of Eq (51) into *M*_1_, since the kernel is compact on this line.

With reference to Eq (52), while warping the integration contour *M* of Eq (51), we need to avoid that the integration contour crosses the spectral observation point *w*, since Eq (52) has been derived from Eq (40) selecting the contour *γ* indented on the right to overcome the PV integration, as described in Step 1. If we warp the integration contour of Eq (52) by crossing point *w*, Eq (52) is not anymore valid. In fact, in this case, the correct formulation of the problem is derived from Eq (40) selecting the contour *γ* indented on the left side, thus yielding Eq (54). Therefore, if one warps the integration contour of Eq (52) crossing point *w*, Eq (52) becomes Eq (54) and viceversa.

Once correctly warped *M* into *M*_1_ in Eq (52) (by keeping point *w* on the left side of the contour), we make *w* tend to *w*′. We note that the kernel *K*_*new*_(*w*, *w*′) is not singular on *M*_1_, i.e. while *w*, *w*′ ∈ *M*_1_. This procedure is useful to obtain FIEs for practical problems where the unknowns are discretized inside and outside the integral sign along the same path, for example *M*_1_.

The contour deformation from *M* into *M*_1_ can capture two kinds of singularities that belong to the strip $-\frac{\pi }{2}<\text{Re}\left[w^{\prime }\right]<0$:

- the non-structural (source) singularities of ${\hat{V}}_{d}\left(w^{\prime }\right)$
- the kernel singularity lines of *K*_*new*_(*w*, *w*′)

For this reason the contour deformation yields further terms out of integration in the integral equations.

In general, while warping the contour *M* into *M*_1_ in Eq (51), we obtain:

$$\begin{array}{c} I=I_{1}+{\hat{I}}_{GO}+I_{K-} \end{array}$$ (55)

The term *I*_1_ is

$$\begin{array}{c} I_{1}=-\frac{Y_{o}}{2\pi j}\int_{M_{1}}\left(\frac{\frac{\pi }{\Phi_{a}}\sin\frac{\pi }{\Phi_{a}}w^{\prime }}{\cos\frac{\pi }{\Phi_{a}}w^{\prime }-\cos\frac{\pi }{\Phi_{a}}w}-\frac{\sin w}{\cos w^{\prime }-\cos w}\right){\hat{V}}_{1d}\left(w^{\prime }\right)dw^{\prime } \end{array}$$ (56)

with *w* on the left of *M*_1_.

${\hat{I}}_{GO}$ and *I*_*K*−_ are the terms respectively due to non-structural (source) singularities and kernel singularities.

Concerning the non-structural singularities, and with reference to an angular region problem with plane wave illumination, we note that these singularities are located in the 2nd and 4th quadrant of proper and improper sheets of plane *η* = −*k* cos *w*, since they correspond to real values of *w* (see Laplace transform Eq (1) and the mathematical definition of a plane wave).

We recall that the *w* plane presents all the Riemann sheets of *η* in a unique plane w: the proper and improper sheets are related to the definition of $\xi =\sqrt{k^{2}-\eta^{2}}$ and they are linked to w region through the mapping *η* = −*k* cos *w*, see Appendix I of [30].

In *w* plane ${\hat{V}}_{1d}\left(w\right)$ shows all the *potential* GO singularities *w*_*GO*_ even if they do not correspond to effective physical GO fields. The number and the location of these singularities depend on the geometry, on the media and on the sources of the entire problem where the angular region of aperture angle Φ_*a*_ under consideration is one of the sub-domains. The poles are related to potential incident, reflected, transmitted, multiple reflected and transmitted waves of the entire problem. The study of these poles requires the complete formulation of the problem since it depends on the whole geometry where the angular region constitutes a sub-domain.

In particular, for example, while considering an angular region terminated by PEC faces −Φ_*b*_ < *φ* < Φ_*a*_, the complete set of singularities *w*_*GO*_ of ${\hat{V}}_{1d}\left(w\right)$ (from (73) of [8]) are

$$\begin{array}{c} w_{GO}=\left\{ \begin{array}{c} \pm (\varphi_{o}+2\left(n-1\right)\Phi_{a}+2n\Phi_{b})\\ \pm (\varphi_{o}-2n\left(\Phi_{a}+\Phi_{b}\right)) \end{array}\right. \end{array}$$ (57)

for n∈Z.

In particular we note that *w*_*o*_ = −*φ*_*o*_ is the pole of the incident wave, *w*_*ra*_ = −2Φ_*a*_ + *φ*_*o*_ is the reflected wave from face A, *w*_*rb*_ = −2Φ_*b*_ − *φ*_*o*_ is the reflected wave from face B, *w*_*rbra*_ = −2Φ_*b*_ + *w*_*ra*_ is the reflected wave first reflected from A than from B, *w*_*rarb*_ = −2Φ_*a*_ + *w*_*rb*_ is the reflected wave first reflected from B than from A…

The contributions ${\hat{I}}_{GO}$ are obtained using the residue theorem

$$\begin{array}{c} {\hat{I}}_{GO}=-Y_{o}\sum_{s}\text{Re}s[\left(\frac{\frac{\pi }{\Phi_{a}}\sin\frac{\pi }{\Phi_{a}}w^{\prime }}{\cos\frac{\pi w^{\prime }}{\Phi_{a}}-\cos\frac{\pi w}{\Phi_{a}}}-\frac{\sin w}{\cos w^{\prime }-\cos w}\right){\hat{V}}_{1d}\left(w^{\prime }\right),w^{\prime }=w_{s}] \end{array}$$ (58)

where *w*_*s*_ are the *w*_*GO*_ with −*π*/2 < *Re*[*w*_*GO*_] < 0, i.e. between *M* and *M*_1_. Note that Eq (58) is exacltly known and computed by substituting ${\hat{V}}_{1d}\left(w\right)$ with ${\hat{V}}_{1d}^{GO}\left(w\right)$ (the GO component of the spectrum), since ${\hat{V}}_{1d}\left(w\right)-{\hat{V}}_{1d}^{GO}\left(w\right)$ is regular in *w* = *w*_*s*_.

Concerning the kernel singularity lines of *K*_*new*_(*w*, *w*′), they are located at *w*′ = ±(*w* + 2*nΦ*_*a*_) and *w*′ = ±(*w* + 2*nπ*) ($n\in Z_{0}$) and therefore they could cross the line *M*_1_ while moving the observation spectral point *w* (as discussed previously we note that the case *n* = 0 is already taken into account in Step 1).

The contributions of *I*_*K*−_ are derived using the residue theorem and the kernel singularities are captured if they cross *M*_1_. Considering the symmetry of the kernel and of ${\hat{V}}_{1d}\left(w\right)$ we obtain

$$\begin{array}{c} \begin{array}{c} I_{K-}=-Y_{o}\sum_{n=1}^{+\infty }\hat{V_{1d}}\left[w+2n\Phi_{a}\right]u\left(-Re\left[w\right]-2n\Phi_{a}+\frac{\pi }{2}\right)-Y_{o}\sum_{n=1}^{+\infty }\hat{V_{1d}}\left[w+2n\pi \right]u\left(-Re\left[w\right]-2n\pi +\frac{\pi }{2}\right)+\\ \;-Y_{o}\sum_{n=1}^{+\infty }\hat{V_{1d}}\left[w+2n\Phi_{a}\right]u\left(-Re\left[w\right]-2n\Phi_{a}-\frac{\pi }{2}\right)+Y_{o}\sum_{n=1}^{+\infty }\hat{V_{1d}}\left[w+2n\pi \right]u\left(-Re\left[w\right]-2n\pi -\frac{\pi }{2}\right)+\\ \;+Y_{o}\sum_{n=1}^{+\infty }\hat{V_{1d}}\left[w-2n\Phi_{a}\right]u\left(Re\left[w\right]-2n\Phi_{a}+\frac{\pi }{2}\right) \end{array} \end{array}$$ (59)

with *Re*[*w*] < 0 and where *u*(⋅) is the unit step function that limits the series to be finite sums.

Note in Eq (59) that, while integrating along *M*_1_, the kernel singularities (*w*′ = ±(*w* + 2*nΦ*_*a*_) and *w*′ = ±(*w* + 2*nπ*)) yield line of singularities that are shifted according to −*π*/2 and parallel to *M*_1_. In general we state an important property: the lines of singularities are always parallel to the integration line in *w* plane even if it is not straight as *M*_1_.

We note that these contributions are independent from the source, i.e. they are structural. Moreover, the number of captured kernel singularities increases with decreasing of the aperture angle Φ_*a*_.

Eq (52) can now be rewritten for *Re*[*w*] < 0 as

$$\begin{array}{c} {\hat{I}}_{1+}\left(w\right)=-Y_{o}{\hat{V}}_{1d}\left(w\right)+I_{1}+{\hat{I}}_{GO}+I_{K-}+{\hat{I}}_{1N+}\left(w\right) \end{array}$$ (60)

Note that each strip defined by unitstep functions of Eq (59) in Eq (60) defines a section of the analytic function ${\hat{I}}_{1+}\left(w\right)$ [44], although ${\hat{I}}_{1+}\left(w\right)$ is an analytic function in the whole *w* plane.

Eq (60) is a Norton equation in *w* plane as it can be rewritten as

$$\begin{array}{c} {\hat{I}}_{1+}\left(w\right)=\hat{Y}\left(w\right)\cdot {\hat{V}}_{1d}\left(w\right)+{\hat{I}}_{1N+}\left(w\right)+{\hat{I}}_{GO} \end{array}$$ (61)

where $\hat{Y}\left(w\right)$ is the admittance operator $\hat{Y}\left(w\right)\cdot {\hat{V}}_{1d}\left(w\right)=-Y_{o}{\hat{V}}_{1d}\left(w\right)+I_{1}+I_{K-}$, ${\hat{I}}_{GO}$ is a current controlled by the GO fields of the entire problem and ${\hat{I}}_{1N+}\left(w\right)$ is the Norton current Eq (35).

**Fig 16:**
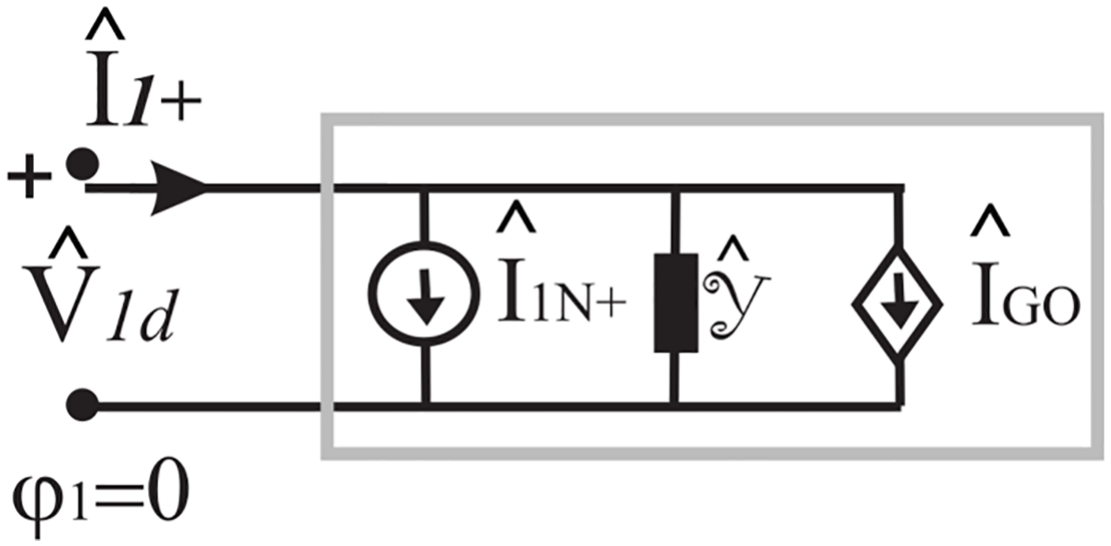
Modified Norton equivalent network corresponding to Eq (61).

The network representation of Eq (61) is reported in Fig 16 and it constitutes a modified Norton representation due to the presence of the controlled generator that depends on the GO terms of the entire problem. We note that the use of controlled generator is due to the warping of integration line from *M* to *M*_1_.

By considering Eqs (54) and (47), we can repeat the whole procedure to derive a Norton model which is valid for *Re*[*w*] > 0 with integration line *M*_1_.

The general form of the modified Norton equation for any w is:

$$\begin{array}{c} {\hat{I}}_{1+}\left(w\right)=sign\left(Re\left[w\right]-\frac{\pi }{2}\right)Y_{o}{\hat{V}}_{1d}\left(w\right)+I_{1}+{\hat{I}}_{GO}+I_{K}+{\hat{I}}_{1N+}\left(w\right) \end{array}$$ (62)

where

$$\begin{array}{c} \begin{array}{c} I_{K}=-Y_{o}\sum_{n=1}^{+\infty }\hat{V_{1d}}\left[w+2n\Phi_{a}\right]u\left(-Re\left[w\right]-2n\Phi_{a}+\frac{\pi }{2}\right)-Y_{o}\sum_{n=1}^{+\infty }\hat{V_{1d}}\left[w+2n\pi \right]u\left(-Re\left[w\right]-2n\pi +\frac{\pi }{2}\right)+\\ \;-Y_{o}\sum_{n=1}^{+\infty }\hat{V_{1d}}\left[w+2n\Phi_{a}\right]u\left(-Re\left[w\right]-2n\Phi_{a}-\frac{\pi }{2}\right)+Y_{o}\sum_{n=1}^{+\infty }\hat{V_{1d}}\left[w+2n\pi \right]u\left(-Re\left[w\right]-2n\pi -\frac{\pi }{2}\right)+\\ \;+Y_{o}\sum_{n=1}^{+\infty }\hat{V_{1d}}\left[w-2n\Phi_{a}\right]u\left(Re\left[w\right]-2n\Phi_{a}+\frac{\pi }{2}\right)-Y_{o}\sum_{n=1}^{+\infty }\hat{V_{1d}}\left[w-2n\pi \right]u\left(Re\left[w\right]-2n\pi +\frac{\pi }{2}\right)+\\ \;+Y_{o}\sum_{n=1}^{+\infty }\hat{V_{1d}}\left[w-2n\Phi_{a}\right]u\left(Re\left[w\right]-2n\Phi_{a}-\frac{\pi }{2}\right)+Y_{o}\sum_{n=1}^{+\infty }\hat{V_{1d}}\left[w-2n\pi \right]u\left(Re\left[w\right]-2n\pi -\frac{\pi }{2}\right) \end{array} \end{array}$$ (63)

We note that the *sign* symbol in Eq (62) select one of the two expressions derived from Eqs (44) and (45). The delimitation is set at *π*/2 since the regularization process has moved the discontinuity from *w* = *w*′ to *w* = −*w*′ and the integration line in Eq (62) is *M*_1_ instead of *M*. Note in Eq (63) that, while integrating along *M*_1_, the kernel singularities (*w*′ = *w* ± 2*nΦ*_*a*_ and *w*′ = *w* ± 2*nπ*) yield lines of singularities that are shifted according to −*π*/2 and parallel to *M*_1_, see the arguments of the unitstep functions.

The Eq (62) is a generalization of Eq (60) that can be interpreted as Norton model Eq (61) which is valid for any w, where $\hat{Y}\left(w\right)$ is the admittance operator

$$\begin{array}{c} \hat{Y}\left(w\right)\cdot {\hat{V}}_{1d}\left(w\right)=sign\left(w-\frac{\pi }{2}\right)Y_{o}{\hat{V}}_{1d}\left(w\right)+I_{1}+I_{K}, \end{array}$$ (64)

${\hat{I}}_{GO}$ is a current controlled by the GO fields of the entire problem and ${\hat{I}}_{1N+}\left(w\right)$ is the Norton current Eq (35), see Fig 16 for the network representation.

We recall that the aim of the proposed procedure is to obtain FIEs for practical problems where the unknowns are discretized inside and outside the integral sign along a same path and therefore we need to pay particular attention on the properties of Eq (62) along the integration path, for example *w* ∈ *M*_1_.

In general, the FIEs in ${\hat{V}}_{1d}\left(w\right)$ are obtained from coupling Norton models of contiguous geometrical subdomains and by eliminating via substitution the unknown currents, i.e ${\hat{I}}_{1+}\left(w\right)$.

For *w* ∈ *M*_1_, we observe that no kernel singularity line is captured, when Φ_*a*_ is obtuse, i.e. *I*_*K*_ = 0 for *w* ∈ *M*_1_ and Φ_*a*_ > *π*/2. In fact taking into account the negative poles *w*′ = −(*w* + 2*nΦ*_*a*_) ($n\in N_{0}$) we observe that these kernel singularities remain on the left of the integration line *M*_1_ if −(*w* + 2*nΦ*_*a*_) < −*π*/2 therefore Φ_*a*_ > *π*/(2*n*) ($n\in N_{0}$). On the contrary, with acute Φ_*a*_ we need to pay attention to the kernel singularities located in −*π*/2 < *Re*[*w*] < 0.

**Fig 17:**
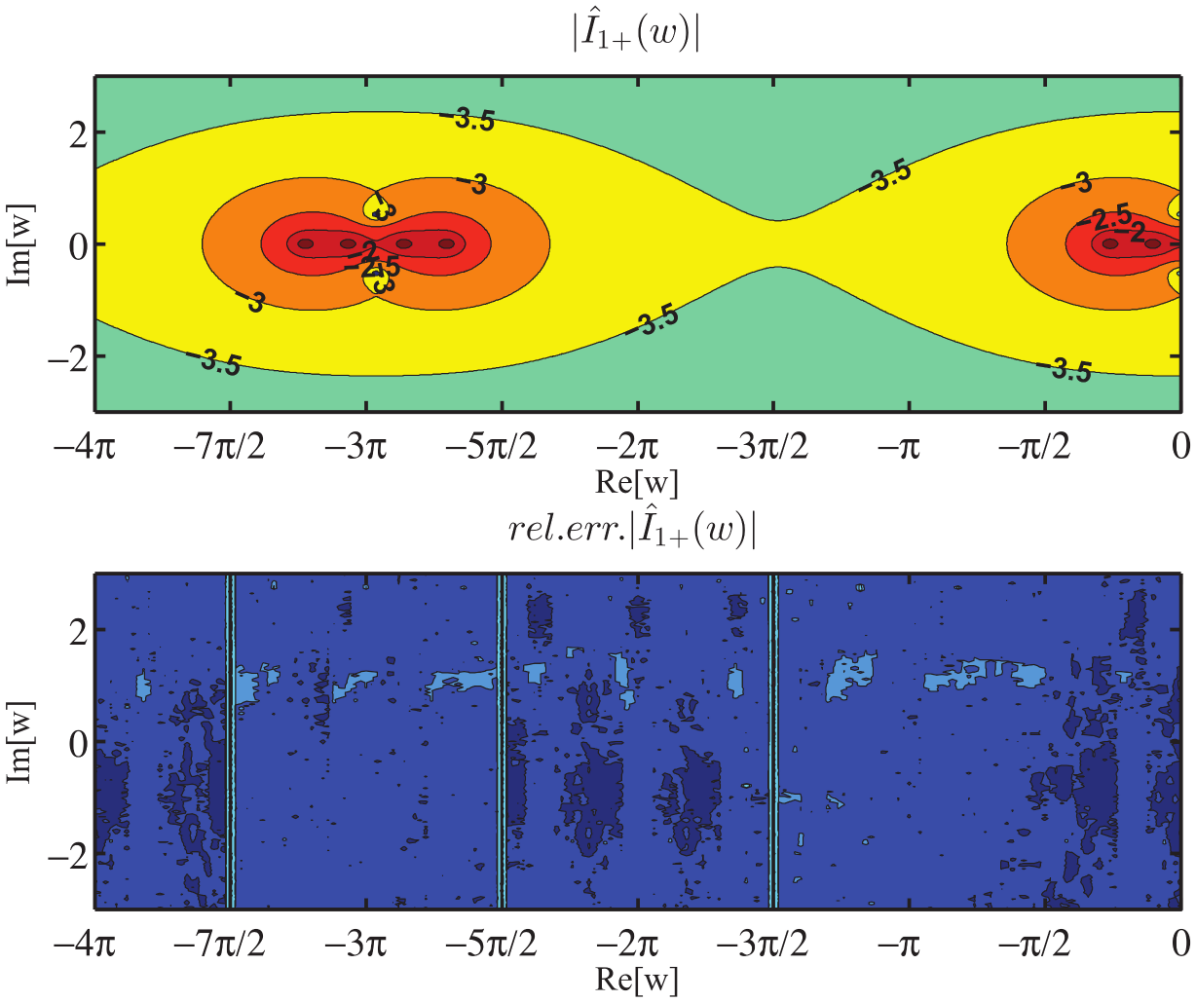
Validation of Eq (62). Absolute value ${\hat{I}}_{1+}\left(w\right)$ in log_10_ scale for −4*π* < *Re*[*w*] < 0, on the bottom the relative error in log_10_ scale obtained from the approximate expression of ${\hat{I}}_{1+}\left(w\right)$ given by Eq (62) while substituting the exact expression of ${\hat{V}}_{1d}\left(w\right)$ (73) of [8] and performing the numerical integration. $\left[\Phi_{a},\Phi_{b},\varphi_{o}\right]=\left[0.\bar{18}\pi ,1.2\pi ,0.1\pi \right]$. The colors at the bottom delimits zone with relative errors lower than 10^−14^ (dark blue), lower than 10^−12^ (blue) and lower than 10^−11^ (light blue).

As final validation of the Norton model in the w plane, we test Eq (62) in the entire complex plane w by computing in log_10_ scale the relative error of the approximated value of ${\hat{I}}_{1+}\left(w\right)$ obtained via numerical integration of Eq (62) when substituting the exact expression of ${\hat{V}}_{1d}\left(w\right)$ for the auxiliary entire problem constituted by a PEC wedge with PEC faces located at *φ* = Φ_*a*_ and *φ* = −Φ_*b*_, see (73) of [8]. In particular, we present in Fig 17 the results for $\left[\Phi_{a},\Phi_{b},\varphi_{o}\right]=\left[0.\bar{18}\pi ,1.2\pi ,0.1\pi \right]$, where the extremely acute angle Φ_*a*_ magnifies the number of kernel singularities captured while moving the spectral observation point w, see Eq (63).

Fig 17 reports on top the absolute value ${\hat{I}}_{1+}\left(w\right)$ in log_10_ scale for −4*π* < *Re*[*w*] < 0, where it is possible to notice the GO poles related to the incident wave *w*_*i*_ = −*φ*_*o*_ and the reflected wave from face A *w*_*RA*_ = −2Φ_*a*_ + *φ*_*o*_. The replica of the spectra is also shown.

The bottom of Fig 17 shows that the relative error is scattered and is approximately around 10^−16^ ÷ 10^−12^ except for the local error along the lines of the kernel singularities *w*′ = ±(*w* + 2*nπ*) ($n\in Z_{0}$) (here *w*′ = −*π*/2 due to the selection of the integration line *M*_1_).

Similar results and errors are obtained considering any aperture angle Φ_*a*_: we observe that the number of kernel singularities and non-structural singularities concentrate toward small value of w for small aperture angles.

We assert that Eq (62) is generally valid with the following limitations: 1) kernel singularities do not belong to *M*_1_, i.e. kernel singular lines do not overlap *M*_1_, 2) singularities are with order 1. Generalization is straightforward.

The complete Norton model in w plane represented by Eq (62) can be re-phrased in *η* plane by carefully applying the inverse mapping *η* = −*k* cos *w* (see [30] for example). We recall that the *w* plane presents all the Riemann sheets of the *η* plane in a unique plane. The pictorial network representation of Fig 16 is also valid in *η* plane.

### 6.4 Step 4

As already stated the solution of practical problems is obtained via the numerical evaluation of FIEs that are sampled and discretized along the same (integration) line. The FIEs are obtained from coupling Norton models of contiguous subdomains and by eliminating via substitution the unknown currents, i.e starting from Eq (62) we eliminate ${\hat{I}}_{1+}\left(w\right)$ in *w* plane.

The presence of *I*_*K*_ Eq (63) in Eq (62) makes Eq (62) an integral-difference equation that is cumbersome to treat, since we need to consider samples of the unknowns along different lines parallel to *M*_1_.

We propose two methods to remove the presence of the difference terms.

The first method is to use a modified *w* plane, i.e. $\bar{w}=w\frac{\pi }{\Phi_{a}}$, and to integrate along the line ${\bar{M}}_{1}$ ($Re\left[\bar{w}\right]=-\frac{\pi }{2}$). Starting from Eq (40) and following Step 1, 2, 3 in $\bar{w}$ with integration line ${\bar{M}}_{1}$, we obtain a new Norton model without the presence of kernel singularity lines. In fact, in this case, the kernel singularities are located at ${\bar{w}}^{\prime }=\pm \left(\bar{w}+2n\pi \right)$ and ${\bar{w}}^{\prime }=\pm \left(\bar{w}+2n\pi^{2}/\Phi_{a}\right)$ with $n\in Z_{0}$. The resulting FIEs do not contain any difference term in $\bar{w}$ plane also in the case of small aperture angles. This method has been extensively used in recent papers for angular regions problems without network representations, see [8, 30, 31, 45].

However, for spectral observation points/samples *w* ∈ *M*_1_ only the first sum in *I*_*K*_ Eq (63) can be different from zero and in particular is non zero if Φ_*a*_ is acute (less than *π*/2). We define

$$\begin{array}{c} I_{K1}=-Y_{o}\sum_{n=1}^{+\infty }\hat{V_{1d}}\left[w+2n\Phi_{a}\right]u\left(-Re\left[w\right]-2n\Phi_{a}+\frac{\pi }{2}\right) \end{array}$$ (65)

Starting from Eq (62) with Eq (65) while sampling and integrating on *M*_1_, the second method rewrites the equation in *η* plane warping the contour from *M*_1_ to the real axis of the plane *η*. It resorts to the inverse mapping of *η* = −*k* cos *w* (see for instance [30]), to the regularity properties of the unknowns and to the application of the Cauchy Theorem in *η* plane. In particular we anticipate that the difference terms in *w* are transformed into integrals in *η*.

In this paper we focus the attention on the second method for two reasons. The first is that we want to illustrate a general procedure to study complex problems constituted by different geometries and media by obtaining simple network representations and the presence of different media does not allow to define an unique *w* plane because of its dependence, by definition, on the propagation constant *k* (see [31] to deal with multiple w planes). The second is that the possible presence of finite layers introduce the need to handle modal representation of the field that in *w* plane exhibits infinite replica of the relevant structural poles. On the contrary, in the *η* plane, the handling of the structural poles is straightforward because of the one-to-one relationship between the structural poles and the modal expansion of the field, see Section 7 for a practical example.

Taking into account Eq (62) with Eq (63), we recall that for vanishing imaginary part of *k*, *M*_1_ is the imaginary axis of *η* plane (*Re*[*η*] = 0) with opposite versus. We can warp *M*_1_ of Eq (62) in *η* plane into the real axis *Im*[*η*] = 0, because no poles are in the 1st and 3rd quadrant of *η* plane, see Figs 13 and 14 of [30]. In fact, concerning the non-structural singularities, and with reference to an angular region problem with plane wave illumination, we note that these singularities are located in the 2nd and 4th quadrant of proper and improper sheets of *η* plane, since they correspond to real values of *w*. The number and the location of these singularities depend on the geometry/media of the problem inside and also outside the angular region under consideration. The study of the singularities requires the complete formulation of the problem where the angular region under consideration is one of the sub-domains.

Moreover the warping in *η* plane does not yield any error in the contribution of the kernel singularities in *I*_*K*_ Eq (63) since, by considering the w plane, also the lines of the kernel singularities are shifted via the deformation (we recall that in *w* plane the kernel singularity lines are parallel to the line of integration, see Step 3). We note that the number of kernel singularities to be considered depends on the spectral observation points.

If the observation points lyes on the integration line (for example the real axis of the *η* plane), the equation preserves the same contributions of captured kernel singularities for integration line *M*_1_ Eq (65).

Conversely, when only the spectral observation point is moved from the integration line to other position in complex plane *η* or *w*, the kernel singularities’ contribution to be considered is dictated by the general term Eq (63).

Therefore, by using the inverse mapping of *η* = −*k* cos *w*, considering the opposite versus of *M*_1_ and for real values of *η*, in the proper *η* plane Eq (62) becomes:

$$\begin{array}{c} \begin{array}{c} Y_{c}\left(\eta \right)\;V_{1+}\left(\eta \right)-I_{1+}\left(\eta \right)+\frac{1}{2\pi j}\int_{-\infty }^{\infty }(\frac{Y_{c}\left(\eta^{\prime }\right)\;}{\alpha \left(\eta^{\prime }\right)-\alpha \left(\eta \right)}\frac{d\alpha }{d\eta^{\prime }}-\frac{Y_{c}\left(\eta \right)\;}{\eta^{\prime }-\eta })V_{1+}\left(\eta^{\prime }\right)d\eta^{\prime }+\\ \;\;\;+\sum_{n=1}^{+\infty }q_{n}\left(\eta \right)V_{1+}\left({\text{p}}_{n}\left(\eta \right)\right)u\left(\pi -2n\Phi_{a}\right)+I_{GO}+I_{1N+}=0 \end{array} \end{array}$$ (66)

for *Im*[*η*] = 0 and where

$$\begin{array}{c} \alpha \left(\eta \right)=-k\cos\left[\frac{\pi }{\Phi_{a}}\arccos\left(-\frac{\eta }{k}\right)\right], \end{array}$$ (67)

$Y_{c}\left(\eta \right)=\frac{1}{Z_{c}\left(\eta \right)}=\frac{\xi (\eta )}{kZ_{o}}$ is the free-space spectral admittance, $\xi \left(\eta \right)=\sqrt{k^{2}-\eta^{2}}$ is the free-space spectral propagation constant (with *ξ*(0) = *k*), *Z*_*o*_ = 1/*Y*_*o*_ is the free space impedance and where

$$\begin{array}{c} p_{n}\left(\eta \right)=-k\cos\left(w+2n\;\Phi_{a}\right)=\eta \cos2n\Phi_{a}-\sqrt{k^{2}-\eta^{2}}\sin2n\Phi_{a} \end{array}$$ (68)

$$\begin{array}{c} q_{n}\left(\eta \right)=\frac{Y_{o}}{k}\left(\eta \sin2n\Phi_{a}+\sqrt{k^{2}-\eta^{2}}\cos2n\Phi_{a}\right) \end{array}$$ (69)

In Eq (66) $I_{1N+}\left(\eta \right)={\hat{I}}_{1N+}\left(w\right)$ is defined in Eqs (39) and (40), and $I_{GO}\left(\eta \right)={\hat{I}}_{GO}\left(w\right)$ in Eq (58).

The terms in the summation reported in Eq (66) comes from the mapping of the difference terms Eq (65) in the *η* plane, i.e.

$$\begin{array}{c} -Y_{o}{\hat{V}}_{1d}\left(w+2n\Phi_{a}\right)=+q_{n}\left(\eta \right)V_{1+}\left({\text{p}}_{n}\left(\eta \right)\right) \end{array}$$ (70)

We recall that, in the derivation of Eq (66), the kernel singularities (that give the difference terms) are captured only for Φ_*a*_ ≤ *π*/(2*n*) with $n\in N_{0}$, see Eq (65). For such values, taking into account that sin(2*nΦ*_*a*_) > 0 and that $Im[\sqrt{k^{2}-\eta^{2}}]<0$ for real *η*, it yields that *p*_*n*_(*η*) for real *η* are located in the upper-half proper sheet of *η* plane, i.e. *Im*[*p*_*n*_(*η*)] > 0, see Eq (68).

From the Cauchy’s integral formula [26] we obtain an exact expression of *V*_1+_(*p*_*n*_(*η*)) while *p*_*n*_(*η*) is located in the upper-half proper *η* plane

$$\begin{array}{c} V_{1+}\left(p_{n}\left(\eta \right)\right)=\frac{1}{2\pi j}\int_{-\infty }^{\infty }\frac{V_{1+}\left(\eta^{\prime }\right)\;}{\eta^{\prime }-p_{n}\left(\eta \right)}d\eta^{\prime }+V_{1+}^{n.s.}\left(p_{n}\left(\eta \right)\right) \end{array}$$ (71)

where the term $V_{1+}^{n.s.}\left(\eta \right)$ is constituted of the non-standard plus singularities due to effective GO terms of the entire problem under investigation (non-standard non-structural poles).

Note that Eq (71) must be interpreted in the proper sheet of *η* plane and it is obtained by closing the contour in the upper-half plane.

By selecting the real axis of *η* plane as integration line and for observation points out of the real axis *η*, *p*_*n*_(*η*) can be located in the lower-half proper *η* plane. Due to Cauchy Theorem, when *p*_*n*_(*η*) is located in the lower-half proper *η* plane, the rhs of Eq (71) is zero.

This behavior also is in accordance with step functions defined in w plane Eq (65), in particular the step functions are successfully implemented in Eq (71) through contour integration in *η* plane.

Substituting Eq (71) into Eq (66) we obtain the Norton equation in *η* plane for real *η* value

$$\begin{array}{c} I_{1+}\left(\eta \right)=Y\left(\eta \right)\cdot V_{1+}\left(\eta \right)+I_{GO}+I_{1N+} \end{array}$$ (72)

where Y(η) is the admittance operator

$$\begin{array}{c} \begin{array}{c} Y\left(\eta \right)\cdot V_{1+}\left(\eta \right)=Y_{c}\left(\eta \right)V_{1+}\left(\eta \right)+\\ \frac{1}{2\pi j}\int_{-\infty }^{\infty }\left(\frac{Y_{c}\left(\eta^{\prime }\right)\;}{\alpha \left(\eta^{\prime }\right)-\alpha \left(\eta \right)}\frac{d\alpha }{d\eta^{\prime }}-\frac{Y_{c}\left(\eta \right)\;}{\eta^{\prime }-\eta }+\sum_{n=1}^{+\infty }\frac{q_{n}\left(\eta \right)}{\eta^{\prime }-{\text{p}}_{n}\left(\eta \right)}u\left(\frac{\pi }{2}-n\Phi_{a}\right)\right)V_{1+}\left(\eta^{\prime }\right)d\eta^{\prime }+\\ -\sum_{n=1}^{+\infty }q_{n}\left(\eta \right)V_{1+}^{n.s.}\left(p_{n}\left(\eta \right)\right)u\left(\pi -2n\Phi_{a}\right) \end{array} \end{array}$$ (73)

We recall that $I_{GO}\left(\eta \right)={\hat{I}}_{GO}\left(w\right)$ is a current controlled by the GO fields of the entire problem Eq (58) and *I*_1*N*+_(*η*) is the Norton current

$$\begin{array}{c} I_{1N+}\left(\eta \right)=-\frac{R_{o}}{\alpha \left(\eta \right)-\alpha \left(\eta_{o}\right)} \end{array}$$ (74)

with *η*_*o*_ = −*k* cos(*φ*_*o*_) and $R_{o}=\frac{j\pi Y_{o}}{\Phi_{a}}2\sin\left(\frac{\pi }{\Phi_{a}}\varphi_{o}\right)$.

The Norton model Eq (72) is obtained for observation points that lye on the integration line (here the real axis of *η* plane), for extension see next Step 5. Also in *η* plane, the FIEs are obtained from coupling Norton models Eq (72) of contiguous subdomains and by eliminating via substitution the unknown currents, i.e *I*_1+_(*η*).

Let us consider three characteristic problems to show the properties of Eq (72): PEC wedges with $\Phi_{a},=0.7\pi ,0.4\pi ,\bar{18}\pi$, Φ_*b*_ = 1.2*π*, *φ*_*o*_ = 0.1*π*. The three problems shows that the number of *I*_*K*1_ terms Eq (65) to be considered in Eq (73) of Eq (72) is respectively 0, 1 and 2.

**Fig 18:**
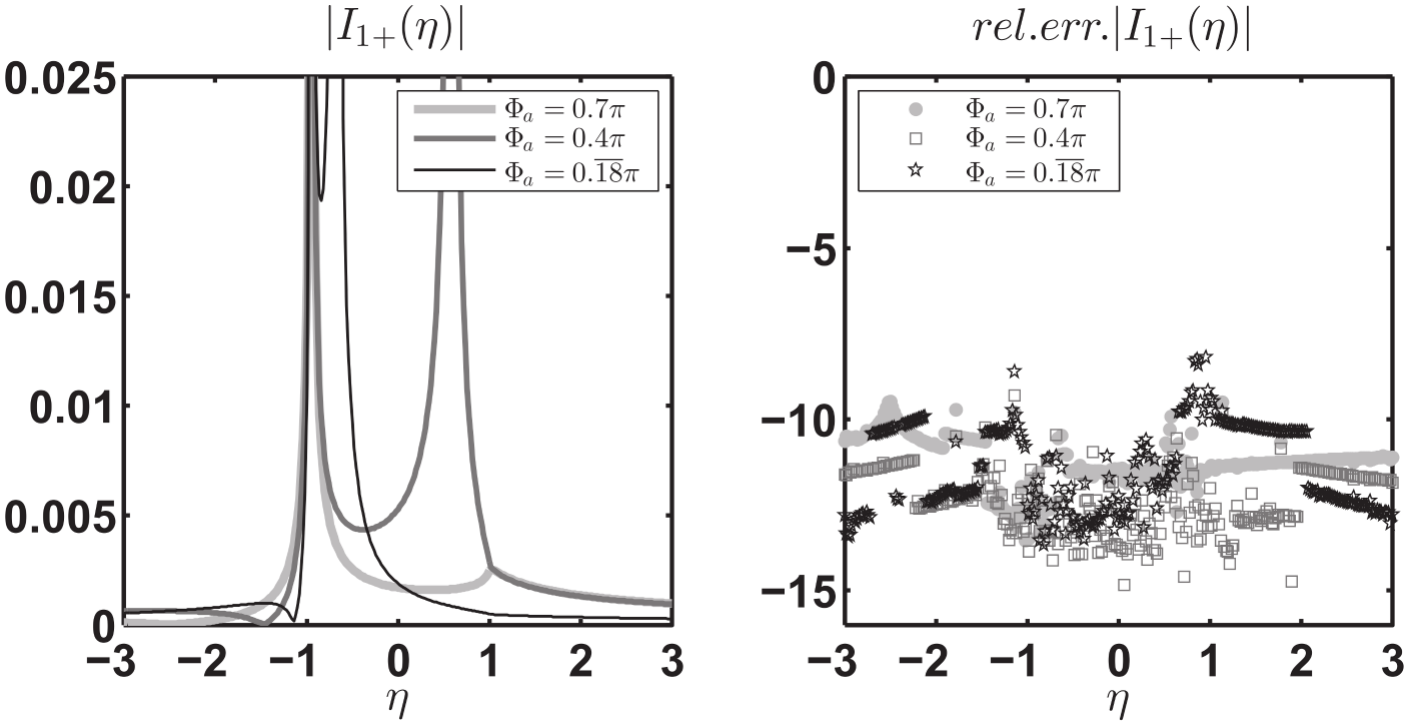
Validation of Eq (72). Left: absolute value of *I*_1+_(*η*). Right: relative error in log_10_ scale obtained from the approximate expression of *I*_1+_(*η*) given by Eq (72) while substituting the exact expression of *V*_1+_(*η*) and performing the numerical integration. Test cases are relative to different sets of parameters: $\Phi_{a},=0.7\pi ,0.4\pi ,0.\bar{18}\pi$, Φ_*b*_ = 1.2*π*, *φ*_*o*_ = 0.1*π*. We recall that *φ* = Φ_*a*_ and *φ* = −Φ_*b*_ are the azimuthal directions of the PEC faces of the wedge and *φ* = *φ*_*o*_ is the azimuthal direction of the incident *E*_*z*_-polarized plane wave.

Fig 18 shows on the left side the approximate spectrum of *I*_1+_(*η*) for real *η* obtained through Eq (72) while substituting the exact spectrum of *V*_1+_(*η*) (taken from (73) of [8] with *η* = −*k* cos *w*) and performing the numerical integration. On the right the relative errors are computed with respect to the exact spectrum of ${\hat{I}}_{1d}\left(w\right)$ (see (74) of [8]).

As expected since we have performed the integration and the testing along the same line, we obtain full convergence of Eq (72) along the real axis *η*.

The selection of the same line for sampling and integrating yields numerical estimation of the unknowns that are valid in selected strips of complex spectral planes: the approximate Norton representations and the approximate solutions of the corresponding FIEs are only analytic elements of analytic functions, i.e the representation are valid only in a section *C*_*o*_ of the complex plane *η* [44]. For example, Eq (72) is valid in a strip containing the real axis *η*. The validity strips are related to the selection of *I*_*K*_ terms Eq (63) according to Φ_*a*_ while sampling and integrating along a specified integration line. For example, while sampling and integrating on *M*_1_ in w plane or on the real axis of *η* plane, we consider just *I*_*K*1_ Eq (65), however, while reconstructing the representation of the solution in the entire complex plane (either w or *η*) we need to consider the entire expression *I*_*K*_ Eq (63) as reported in Eq (62) in w plane and in the related expression for *η* plane. We recall the kernel singularity lines due to *I*_*K*_ Eq (63) are lines parallel to the selected integration lines.

### 6.5 Step 5

**Fig 19:**
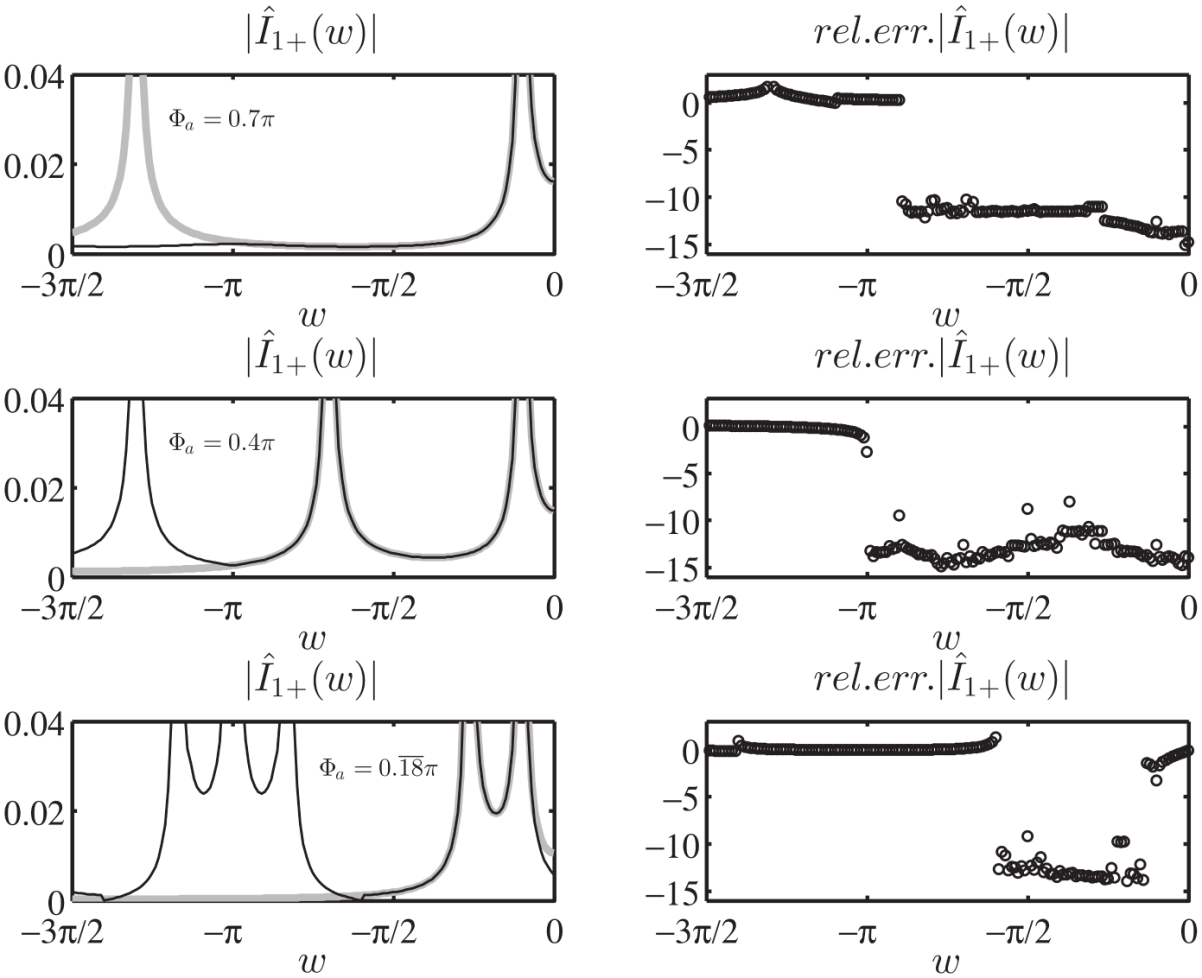
Validation of Eq (72) along the real axis w, by substituting *η* = −*k* cos *w*. Left: absolute value of $|{\hat{I}}_{1+}\left(w\right)|$ (exact = thick solid gray line, approximate = solid black line). Right: relative error in log_10_ scale obtained from the approximate expression of *I*_1+_(*η*) for *η* = −*k* cos *w* given by Eq (72) while substituting the exact expression of *V*_1+_(*η*) and performing the numerical integration. Test cases are relative to different sets of parameters: $\Phi_{a},=0.7\pi ,0.4\pi ,0.\bar{18}\pi$, Φ_*b*_ = 1.2*π*, *φ*_*o*_ = 0.1*π*. We recall that −*π* < *w* < 0 is the portion of real axis of w plane in the proper sheet of *η* plane.

The Norton representation obtained for real *η* Eq (72), see Fig 18, provides accurate spectra only in a region *C*_*o*_ near the real axis of *η* plane. In particular, Fig 19 shows that the validity of Eq (72) is limited for real values of *w* and in general for complex values of *w* such that *η* ∈ *C*_*o*_ with *η* = −*k* cos *w*. The size and the shape of *C*_*o*_ is defined by the kernel singularity lines *I*_*K*_ Eq (63) originated from the singularity of the kernel Eq (53) in Eq (62) in *w* plane and its transposition in *η* plane (see step 4).

However a complete and rigorous theory for the class of problems reported in Fig 3 requires the knowledge of the spectra in a region of complex plane *η* that is wider than *C*_*o*_ and that includes also the improper sheet of *η* plane.

In this framework, we state that both complex planes, *w* and *η*, are important to derive: 1) the formulations of the problem, 2) the subdomain (section) of complex planes where the numerical representations/solutions holds, 3) the physical properties of the solutions, 4) the field components.

For a general problem constituted by angular regions coupled with layered regions, we obtain the complete spectra of the problem through the knowledge of at least a starting approximated spectra in the fundamental strip *S*_*o*_ (−*π* < *Re*[*w*] < 0) through suitable recursive equations obtained by the GWHEs of the entire problem (see Section 7 for a practical case). We note that, in order to effectively apply recursive equations in problems restricted to angular regions the initial approximate spectra can be limited to −Φ_*a*_ < *Re*[*w*] < 0 (see applications in [30] and [31]), on the contrary when angular regions are coupled to layered regions we need −*π* < *Re*[*w*] < 0 due to the presence of layers.

Since initial approximate representations in the entire complex plane *w* and *η* are difficult to be obtained, we resort to extended spectra that are necessary to the computation of several field components: reflected and refracted plane waves, diffracted waves, surface waves, lateral waves, leaky waves, mode excitations, near field and so on…

Fortunately we experienced that, to complete the analysis of field in several practical cases, it is sufficient to know the spectra in limited regions even smaller than *S*_*o*_.

Since the image of *C*_*o*_ in the *w* plane is only an initial strip *S*_*s*_ ⊂ *S*_*o*_ (see for practical examples Fig 19), the aim of step 5 is to resort to Eq (62) with Eq (63) that is valid in the entire complex plane *w*, to enlarge the image of *C*_*o*_ to a strip useful to estimate the fields components and possibly to the fundamental strip *S*_*o*_. We recall the kernel singularity lines due to *I*_*K*_ Eq (63) are lines parallel to the selected integration lines.

Although the validity regions are clear in *w* plane see Eq (62) with Eq (63), the possibility to extend the approximate expression obtained in *η* plane in wider strip can be obtained first by dealing with kernel singularities in *η* plane, second by resorting to difference equations in *w* plane (obtained from the WH formulation) once the initial spectra is sufficient, i.e. in general problems −*π* < *w* < 0.

We note that limiting the required strip to *S*_*o*_ simplifies the general form of Norton representation Eq (62), in particular in Eq (63) for in −*π* < *Re*[*w*] < 0 the 2nd, the 4th, the 6th, the 7th and the 8th sums are zero due the unit step functions. We focus the attention of the singularities lines given by the 1st, the 3rd and the 5th sums in Eq (63) which are respectively $w=-2n\Phi_{a}+\frac{\pi }{2}$, $w=-2n\Phi_{a}-\frac{\pi }{2}$, $w=2n\Phi_{a}-\frac{\pi }{2}$ and respectively related to the kernel singularities *p*_*n*_(*η*) = −*k* cos(*w* + 2*nΦ*_*a*_) Eq (68), ${\tilde{p}}_{n}\left(\eta \right)=-k\cos\left(w+2n\Phi_{a}\right)$ and *p*_−*n*_(*η*) = −*k* cos(*w* − 2*nΦ*_*a*_) with $n\in N_{0}$. Notice that *p*_*n*_(*η*) and ${\tilde{p}}_{n}\left(\eta \right)$ are the same kernel singularity but their effect is shifted in *w* plane due to the step functions (see Eq (63)).

The initial strip *S*_*s*_ and the extended strips (while considering additional kernel singularities) strongly depend on Φ_*a*_. These strips in *η* plane are cumbersome regions that usually span the proper and improper sheets of *η* plane (the sheets are related to the definition of $\xi =\sqrt{k^{2}-\eta^{2}}$ and they are linked to *w* region through the mapping *η* = −*k* cos *w*, see Appendix I of [30]).

Moreover the location of kernel singularities *p*_*n*_(*η*) can span from the upper half plane to the lower half plane or the improper sheet of the *η* plane.

According to the Cauchy’s integral theorem, when *p*_*n*_(*η*) goes into the lower half plane, Eq (71) is not a valid representation of *V*_1+_(*p*_*n*_(*η*)) and it must be replaced by

$$\begin{array}{c} V_{1+}\left(p_{n}\left(\eta \right)\right)=\frac{1}{2\pi j}\int_{-\infty }^{\infty }\frac{V_{1+}\left(\eta^{\prime }\right)\;}{\eta^{\prime }-p_{n}\left(\eta \right)}d\eta^{\prime }+\frac{1}{2\pi j}\int_{b_{r}}\frac{V_{1+}\left(\eta^{\prime }\right)\;}{\eta^{\prime }-p_{n}\left(\eta \right)}d\eta^{\prime }+V_{1+}^{s.}\left(p_{n}\left(\eta \right)\right) \end{array}$$ (75)

where the term $V_{1+}^{s.}\left(\eta \right)$ is constituted of the standard plus singularities due to effective GO terms of the entire problem under investigation (standard non-structural poles) and *b*_*r*_ is the contour around the structural branch lines of the branch points *k*. We notice that in presence of dishomogeneous media we have multiple *k* to be handled, one for each material. Note that Eq (75) must be interpreted in the proper sheet of *η* plane and it is obtained by closing the contour in the lower-half plane.

The number and the location of kernel singularities to be considered are strongly dependent on the spectral observation points and on the aperture angle Φ_*a*_. We have recognized three characteristic ranges of aperture angle Φ_*a*_ by the properties of the spectral unknowns for real negative w: obtuse *π*/2 < Φ_*a*_ < *π*, acute *π*/4 < Φ_*a*_ < *π*/2, *hyper-acute* 0 < Φ_*a*_ < *π*/4.

For obtuse angle no *I*_*K*1_ term Eq (65) needs to be considered in numerical estimation of the Norton model Eq (72) for real *η*, instead, while numerically reconstructing the current in −*π* < *w* < 0 we need to consider *I*_*K*_ Eq (63). For acute and hyper-acute angles we need to consider *I*_*K*1_ terms in the Norton model and *I*_*K*_ in reconstructing the current in −*π* < *w* < 0. For hyper-acute angles some of the kernel singularities can move from the upper half *η* plane to the lower half for −*π* < *w* < 0 (we recall that for real *η* the captured kernel singularities *p*_*n*_(*η*) lies on the upper half *η* plane).

The same properties holds for the numerical solution of the to be-defined FIEs in terms of voltage unknowns since they are obtained by coupling Norton models and eliminating the current unknowns.

As shown in Fig 19 for the three test cases, we have that the region −*π* < *Re*[*w*] < 0 is limited for test case 1 (Φ_*a*_, = 0.7*π*) on the left by $-2\Phi_{a}+\frac{\pi }{2}$ (i.e. $-2.827=-2\Phi_{a}+\frac{\pi }{2}<Re\left[w\right]<0$), for test case 2 (Φ_*a*_, = 0.4*π*) it is not limited (i.e. −*π* < *Re*[*w*] < 0), for test case 3 ($\Phi_{a},=0.\bar{18}\pi$) it is limited on the left by $-2\times 3\Phi_{a}+\frac{\pi }{2}$ and on the right by $+2\Phi_{a}-\frac{\pi }{2}$ (i.e. $-1.856=-2\times 3\Phi_{a}+\frac{\pi }{2}<Re\left[w\right]<+2\Phi_{a}-\frac{\pi }{2}=-0.428$)

By considering all the above properties, starting form Eq (62) with Eq (63) defined in w plane, we extend the validity of Eq (72) in *η* plane to model the approximation in −*π* < *w* < 0 considering the kernel singularities *p*_*n*_ and ${\tilde{p}}_{n}$ via Eq (71) and (75) and applying unite step functions in w plane as reported in Eq (63).

Therefore an extended form of Eq (66) while *η* = −*k* cos *w* with −*π* < *w* < 0 is:

$$\begin{array}{c} \begin{array}{c} Y_{c}\left(\eta \right)\;V_{1+}\left(\eta \right)-I_{1+}\left(\eta \right)+\frac{1}{2\pi j}\int_{-\infty }^{\infty }(\frac{Y_{c}\left(\eta^{\prime }\right)\;}{\alpha \left(\eta^{\prime }\right)-\alpha \left(\eta \right)}\frac{d\alpha }{d\eta^{\prime }}-\frac{Y_{c}\left(\eta \right)\;}{\eta^{\prime }-\eta })V_{1+}\left(\eta^{\prime }\right)d\eta^{\prime }+\\ \;\;\;+\sum_{n=1}^{+\infty }q_{n}\left(\eta \right)V_{1+}\left({\text{p}}_{n}\left(\eta \right)\right)u\left(-Re\left[w\right]-2n\Phi_{a}+\frac{\pi }{2}\right)+\\ \;\;\;+\sum_{n=1}^{+\infty }q_{n}\left(\eta \right)V_{1+}\left({\tilde{\text{p}}}_{n}\left(\eta \right)\right)u\left(-Re\left[w\right]-2n\Phi_{a}-\frac{\pi }{2}\right)+\\ \;\;\;+\sum_{n=1}^{+\infty }q_{-n}\left(\eta \right)V_{1+}\left({\text{p}}_{-n}\left(\eta \right)\right)u\left(Re\left[w\right]-2n\Phi_{a}+\frac{\pi }{2}\right)+I_{GO}+I_{1N+}=0 \end{array} \end{array}$$ (76)

Note that Eq (76) is written in *η* although it contains unit step functions in *w* plane that are cumbersome to be mapped in *η* plane. The presence of unitstep functions limits the series to be finite sums and the numbers of terms strongly depends on Φ_*a*_.

We recall that for kernel singularities *p*_*n*_ located in the upper half *η* plane we need to use Eq (71) in Eq (76), on the contrary for kernel singularities in the lower half *η* plane we need to use Eq (75).

In the last case the rigorous numerical representation of *I*_1+_(*η*) via Eq (76) requires the integration of *V*_1+_(*η*) along the real axis of *η* plane together with the branch lines *b*_*r*_ that requires some effort to be numerically implemented.

To obtain an integral representation that do not sample branch lines, we can approximate *V*_1+_(*p*_*n*_(*η*)) with *p*_*n*_(*η*) in the lower-half *η* plane with different strategies: 1) shifting the contour of Eq (71) below *p*_*n*_(*η*) and neglecting the horizontal integration line, i.e. we approximate *V*_1+_(*p*_*n*_(*η*)) with captured GO poles; 2) closing the contour in the lower-half plane and neglecting the integration along *b*_*r*_ lines, see Eq (75); 3) using approximation based on continuing *V*_1+_(*p*_*n*_(*η*)) expression Eq (71) by fixing the pole *p*_*n*_(*η*) that goes in the lower half *η* plane just above the real axis *η* plane, i.e. for example at −*k*/100. In particular in the following numerical example we will demonstrate the performance of the 3rd method that ensures continuity in the transition region and keeps errors limited.

#### 6.5.1 Numerical tests

In Table 1 we have reported all the properties of kernel singularities of the Norton model Eq (72) and their influence in its analytical extension Eq (76) for real value of *w* in −*π* < *w* < 0 for the three characteristic test cases reported in the previous Sections, i.e. PEC wedges with $\Phi_{a},=0.7\pi ,0.4\pi ,\bar{18}\pi$, Φ_*b*_ = 1.2*π*, *φ*_*o*_ = 0.1*π*. Moreover Table 1 shows the same properties for a fourth test case in a more general form when Φ_*a*_ is reduced to *π*/9. As expected the decreasing of Φ_*a*_ increases the number of captured kernel singularities, i.e. reduces the dimension of the section where Eq (72) is analytic by itself.

**Table 1:** Properties of kernel singularities and kernel singularity lines (we omit to report the dependence in *η* or *w* of kernel singularities).

test case n.	Φ_*a*_	captured *p*_*n*_ while sampling and integrating along the same line (e.g. *w* ∈ *M*_1_, real axis *η*)	captured *p*_*n*_ and ${\tilde{p}}_{n}$ while moving obs. point w in −*π* < *w* < 0	properties of captured *p*_*n*_ and ${\tilde{p}}_{n}$ while moving obs. point w in −*π* < *w* < 0	location in upper/lower *η* plane of captured *p*_*n*_ and ${\tilde{p}}_{n}$ while moving obs. point w in −*π* < *w* < 0
1	0.7*π*	none	*p*_1_	*p*_1_ starts at $w=-2\Phi_{a}+\frac{\pi }{2}=-2.827$ toward negative values	*p*_1_ in upper half plane
2	0.4*π*	*p*_1_	*p*_1_	*p*_1_ starts at $w=-2\Phi_{a}+\frac{\pi }{2}=-0.942$ toward negative values	*p*_1_ in upper half plane
3	$0.\bar{18}\pi$	*p*_1_, *p*_2_	$p_{1},\;p_{2},\;p_{3},\;p_{4},\;p_{-1},\;{\tilde{p}}_{1}$	*p*_1_ starts at $w=-2\Phi_{a}+\frac{\pi }{2}=0.428$; *p*_2_ starts at $w=-4\Phi_{a}+\frac{\pi }{2}=-0.714$; *p*_3_ starts at $w=-6\Phi_{a}+\frac{\pi }{2}=-1.856$; *p*_4_ starts at $w=-8\Phi_{a}+\frac{\pi }{2}=-2.999$; *p*_−1_ starts at $w=2\Phi_{a}-\frac{\pi }{2}=-0.428$; ${\tilde{p}}_{1}$ starts at $w=-2\Phi_{a}-\frac{\pi }{2}=-2.713$; $p_{1},p_{2},p_{3},p_{4},{\tilde{p}}_{1}$ have support toward negative real values of w, while *p*_−1_ has support toward positive real value	*p*_2_, *p*_3_, *p*_4_, *p*_−1_ in upper half plane; *p*_1_ in upper half plane and goes into the lower half plane at $w=-2\Phi_{a}-\frac{\pi }{2}=-2.713$; ${\tilde{p}}_{1}$ in lower half plane
4	$0.\bar{1}\pi$	*p*_1_, *p*_2_, *p*_3_, *p*_4_	*p*_1_, *p*_2_, *p*_3_, *p*_4_, *p*_5_, $p_{6},\;p_{-1},\;p_{-2},\;{\tilde{p}}_{1},\;{\tilde{p}}_{2}$	*p*_*n*_ with *n* = 1, 2, 3, 4, 5, 6, start at $w=-2n\Phi_{a}+\frac{\pi }{2}$; ${\tilde{p}}_{n}$ with *n* = 1, 2, start at $w=-2n\Phi_{a}-\frac{\pi }{2}$; *p*_*n*_ with *n* = −1, −2, start at $w=-2n\Phi_{a}+\frac{\pi }{2}$; *p*_*n*_ with *n* = 1, 2, 3, 4, 5, 6 and ${\tilde{p}}_{n}$ with *n* = 1, 2 have support toward negative real values of w while *p*_*n*_ with *n* = −1, −2 have support toward positive real value	*p*_*n*_ with *n* = 3, 4, 5, 6, −1, −2 in upper half plane; *p*_*n*_ with *n* = 1, 2 in upper half plane and goes into the lower half plane at $w=-2n\Phi_{a}-\frac{\pi }{2}$; ${\tilde{p}}_{n}$ with *n* = 1, 2 in lower half plane

Note that the general properties of captured structural singularities are derived from Eq (63). For example the number of captured kernel singularities *p*_*n*_ while the observation points and the integration points lies on the same line (e.g. *w* ∈ *M*_1_, real axis *η*) are 0 if *π*/2 < Φ_*a*_ < *π* (obtuse angular region) and *n* if *π*/(2(*n* + 1)) < Φ_*a*_ < *π*/(2*n*) for $n\in N_{0}$ (acute angular region). A more complex rule holds for structural singularities *p*_*n*_, ${\tilde{p}}_{n}$ and *p*_−*n*_ when the observation point is moved in −*π* < *w* < 0 as shown in the test cases reported in Table 1.

To avoid more cumbersome properties the line of integration must be regular, monotonic and cross the real *w* axis in one point. This requirement allows to deduce the property of kernel singularities for −*π* < *w* < 0.

Let us examine in detail test case 3 of Figs 18 and 19, i.e. $\left[\Phi_{a},\Phi_{b},\varphi_{o}\right]=\left[0.\bar{18}\pi ,1.2\pi ,0.1\pi \right]$, that is able to show all the properties of the equations. From Table 1 we notice that Eq (72) contains *p*_1_(*η*) and *p*_2_(*η*) Eq (68) (third column).

From Eq (72), the validity segment (real *w*) for test case 3 is $-1.856=-2\times 3\Phi_{a}+\frac{\pi }{2}<w<+2\Phi_{a}-\frac{\pi }{2}=-0.428$ since Eq (72) does not contain any further structural poles *p*_*n*_ for integer *n* > 2 and *n* < 0 or ${\tilde{p}}_{n}$, that become necessary for analytical extension in w plane as clear in Eq (62) with Eq (63) and clearly reported in Eq (76) (see also fourth and fifth column of Table 1).

The two kernel singularities *p*_1_ and *p*_2_, while moving the observation point along the real axis of *η* plane, stay in the upper-half *η* plane. While moving the observation point along the real axis of *w* plane for −*π* < *w* < 0 the pole *p*_2_ starts in the lower *η* plane at *w* = 0 and then it goes to the upper *η* plane at $w=-2\times 2\Phi_{a}+\frac{\pi }{2}=-0.714$, on the contrary the pole *p*_1_ starts in the upper *η* plane at *w* = 0 and then it goes to the lower *η* plane at $w=-2\Phi_{a}-\frac{\pi }{2}=-2.713$. This property is expected in test case 3 since $\Phi_{a}<\frac{\pi }{4}$, see Eq (63) (see sixth column in Table 1).

At the same time ${\tilde{p}}_{1}\left(\eta \right)$ is activated by the unit step functions Eq (76) when *p*_1_ goes into the lower half plane, doubling its effect (see sixth column in Table 1). This properties is general and the singularity line related to each ${\tilde{p}}_{n}$, i.e. $Re\left[w\right]=-2n\Phi_{a}-\frac{\pi }{2}$, corresponds to the real value for which *p*_*n*_ goes into the lower half plane together with ${\tilde{p}}_{n}$.

In test case 3, we note that for observation point −*π* < *w* < 0 we need to consider the contributions of *p*_*n*_ with *n* = 3, 4 that are activated respectively for $w<-2\times n\Phi_{a}+\frac{\pi }{2}=-1.856,-2.999$ and the contribution of *p*_*n*_ with *n* = −1 that is activated respectively for $w>2\Phi_{a}-\frac{\pi }{2}=-0.428$ as reported in Eq (76) (see also Table 1 for a summary).

**Fig 20:**
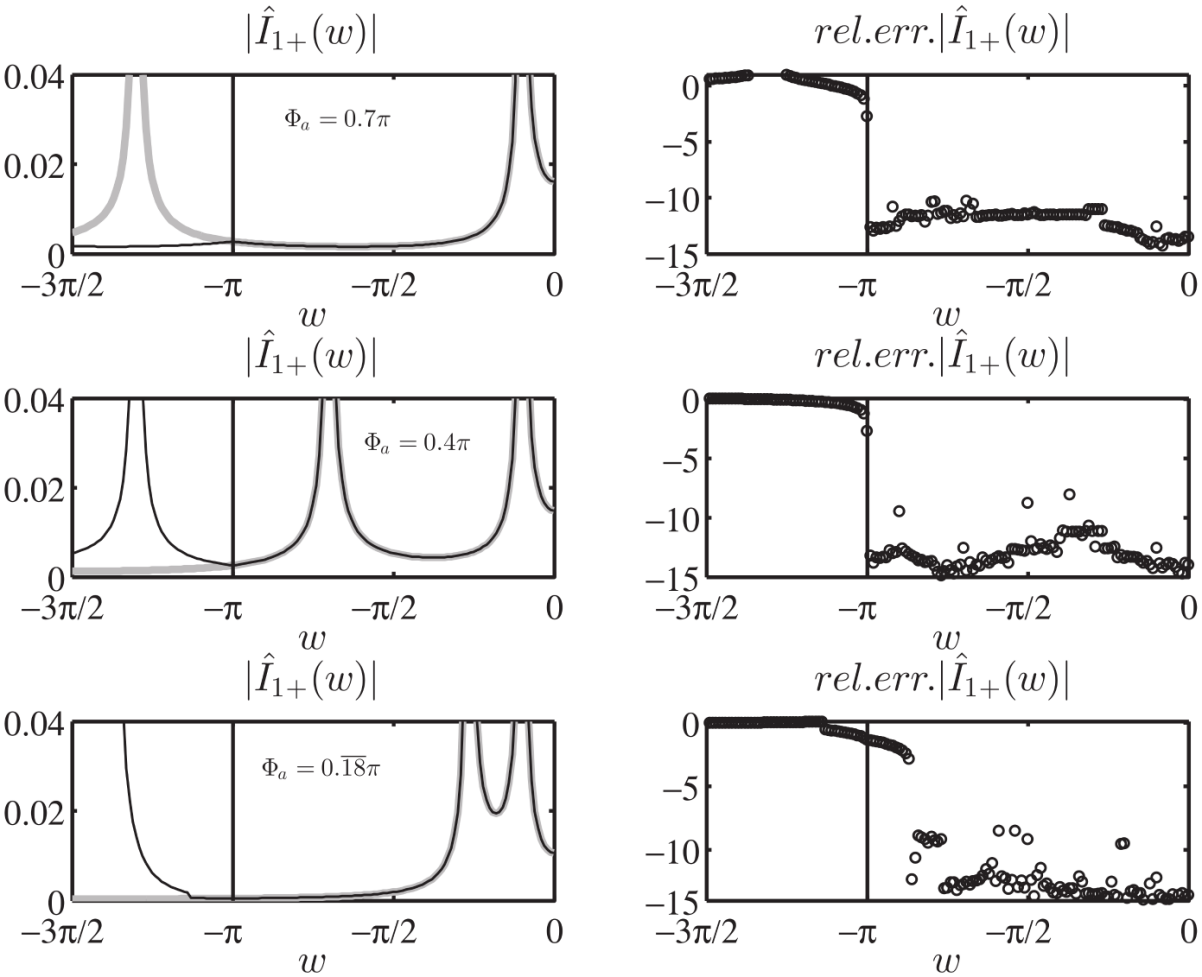
Validation of Eq (66) along the real axis w, by substituting *η* = −*k* cos *w*. Left: absolute value of $|{\hat{I}}_{1+}\left(w\right)|$ (exact = thick solid gray line, approximate = solid black line). Right: relative error in log_10_ scale obtained from the approximate expression of *I*_1+_(*η*) for *η* = −*k* cos *w* given by Eq (66) while substituting the exact expression of *V*_1+_(*η*), performing the numerical integration and considering the full properties of *V*_1+_(*p*_*n*_(*η*)) in w plane. Test cases are relative to different sets of parameters: $\Phi_{a},=0.7\pi ,0.4\pi ,0.\bar{18}\pi$, Φ_*b*_ = 1.2*π*, *φ*_*o*_ = 0.1*π*. We recall that −*π* < *w* < 0 is the portion of real axis of w plane in the proper sheet of *η* plane.

Fig 20 shows the validation of the general expression Eq (76) for −*π* < *w* < 0 for the three considered characteristic test cases. The results can be compared with the ones reported in Fig 19 obtained through Eq (72).

In particular we recall that for Φ_*a*_ < *π*/4 at least one *p*_*n*_ changes location in the half *η* planes. As expected we have full convergence when the kernel singularities lie on upper half *η* plane (see test case 1 and 2).

Since we have implemented an approximated technique (see previous subsection) to handle kernel singularities that lie in the lower half *η* plane for spectral observation points *w* that go toward *w* = −*π* (see test case 3) we experience a loss of precision moving away from the region where all the kernel singularities are in the upper half *η* plane.

However the loss of precision in test case 3 is localized for a restricted portion of spectra and the relative error is of the order of 10^−2^ ÷ 10^−3^ that is acceptable in asymptotic estimations as demostrated in Section 6.6.

### 6.6 An example of implementation of FIE: The PEC wedge

**Fig 21:**
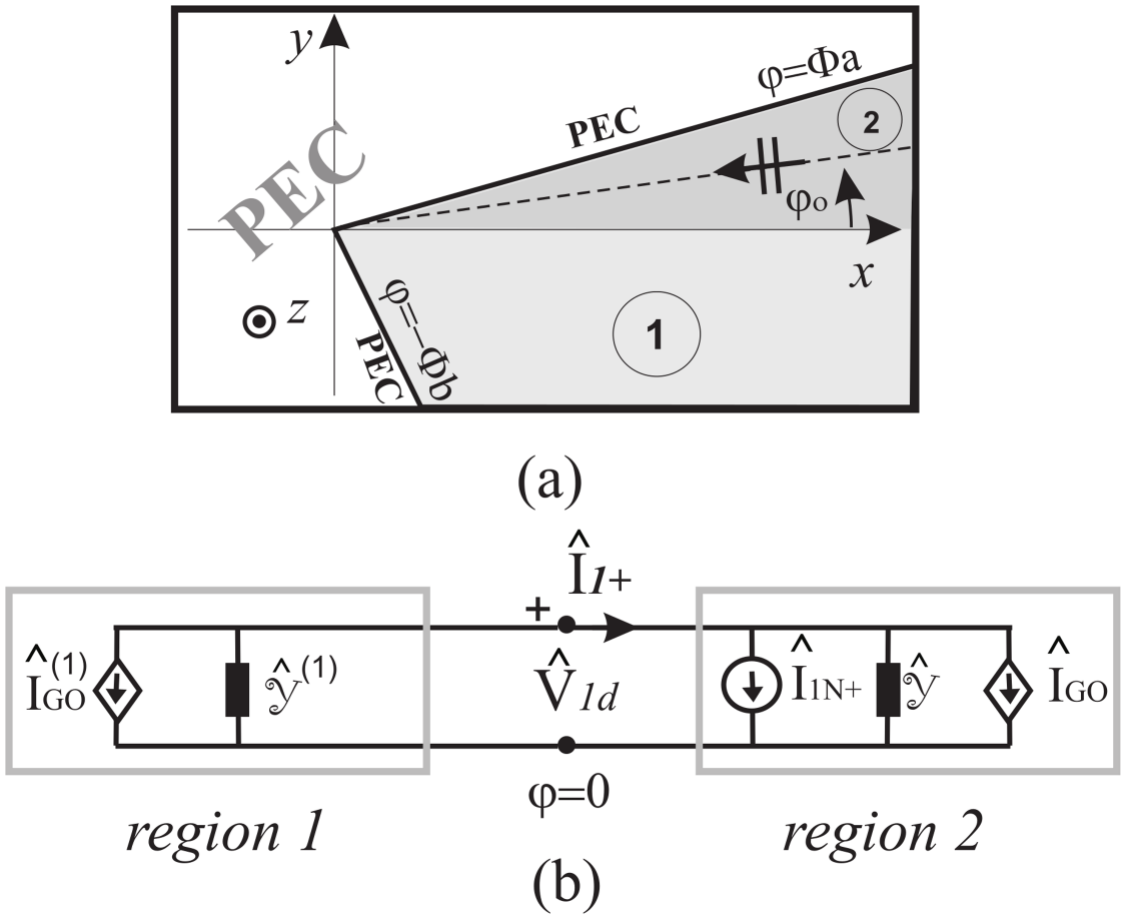
The Concave PEC wedge. (a) Geometry of the problem subdivided into two angular regions: 1) −Φ_*b*_ < *φ* < 0 and 2) 0 < *φ* < Φ_*a*_; (b) Network model using coupling of modified Norton equivalent circuits.

As an example of validation, let us consider a PEC wedge (−Φ_*b*_ < *φ* < Φ_*a*_) in a homogenous medium and illuminated by an *E*_*z*_ polarized plane wave with direction 0 < *φ*_*o*_ < Φ_*a*_, see Fig 21a. We can subdivide the homogeneous angular region into two angular regions: 1) −Φ_*b*_ < *φ* < 0 and 2) 0 < *φ* < Φ_*a*_.

For region 2, the previous Sections illustrate the procedure to obtains a Norton model in w and *η* planes, starting form Eq (40) with Fig 13b and obtaining Eq (62) with Fig 16 and Eq (66).

Following the same procedure for region 1, by noticing that no concentrated source is present for −Φ_*b*_ < *φ* < 0 and that the current ${\tilde{I}}_{1+}$ has opposite versus in the one port Norton model, we obtain the same Norton equations with the following differences: 1) the sign of the current ${\tilde{I}}_{1+}$, 2) absence of ${\tilde{I}}_{1N+}$, 3) Φ_*a*_ must be replaced by Φ_*b*_, 4) the structural pole contributions depend on the amplitude of Φ_*b*_ instead of Φ_*a*_.

The complete network model is presented in Fig 21b where a concave wedge is reported. We have selected a concave wedge with small aperture angle Φ_*a*_ + Φ_*b*_ to highlight all the properties of the Norton model for angular regions, in particular for small angular regions.

By summing the equations obtained for region 1 and 2 we eliminate ${\tilde{I}}_{1+}$ obtaining an equation in ${\tilde{V}}_{1+}$, in particular in ${\hat{V}}_{1d}\left(w\right)$ (integral -difference equation) and *V*_1+_(*η*) (Fredholm integral equation).

To validate the FIE for real w values, we have compared the exact spectrum ${\hat{V}}_{1d}\left(w\right)$, with one obtained numerically through the FIE by substituting inside the integral the exact expression of *V*_1+_(*η*) (for the exact spectrum see (73) of [8]).

Fredholm theory guarantees the convergence via numerical procedure of integral equations of second kind [43]. Since the kernel of the FIE presents well suited behavior, we use a simple sample and hold quadrature scheme to obtain accurate and stable numerical solutions: we apply uniform sampling *f*(*hi*) with $i=-\frac{A}{h}..\frac{A}{h}$ and modified left-rectangle numerical integration formula $\int_{-\infty }^{\infty }f\left(u\right)du\approx h\sum_{i=-A/h}^{A/h}f(h\;i)$ where *A* and *h* are respectively the truncation parameter and the step parameter for the integrals in *u*. This rule has been successfully applied in wedge problems [30, 31, 45]. The total number of samples is *N* = 2*A*/*h* + 1. We observe that as *A* → +∞ and *h* → 0, the numerical solution of the FIE converges to the exact solution [43]; consequently *h* has to be chosen as small as possible and *A* has to be chosen as large as possible. In practical numerical implementations *A* and *h* are finite and they are selected according to the kernel behavior (spectral bandwidth and shape) as demonstrated in the numerical validations and examples: for instance see next Section 7 which reports effective values of *A* and *h*. To improve the accuracy of the numerical procedure, in general, we use the expedient described in [3, 4, 32] based on contour warping.

Once obtained the numerical solution in terms of approximated spectrum of *V*_1+_(*η*), we can obtain the approximate spectrum of *I*_1+_(*η*) through the direct application of the Norton equation in *η* plane either of the angular region 1 or of the angular region 2. The validity of the approximated expressions are ensured for −*π* < *w* < 0 and in sections of the proper sheet of *η* plane according to the singularity lines related to the selected integration line.

**Fig 22:**
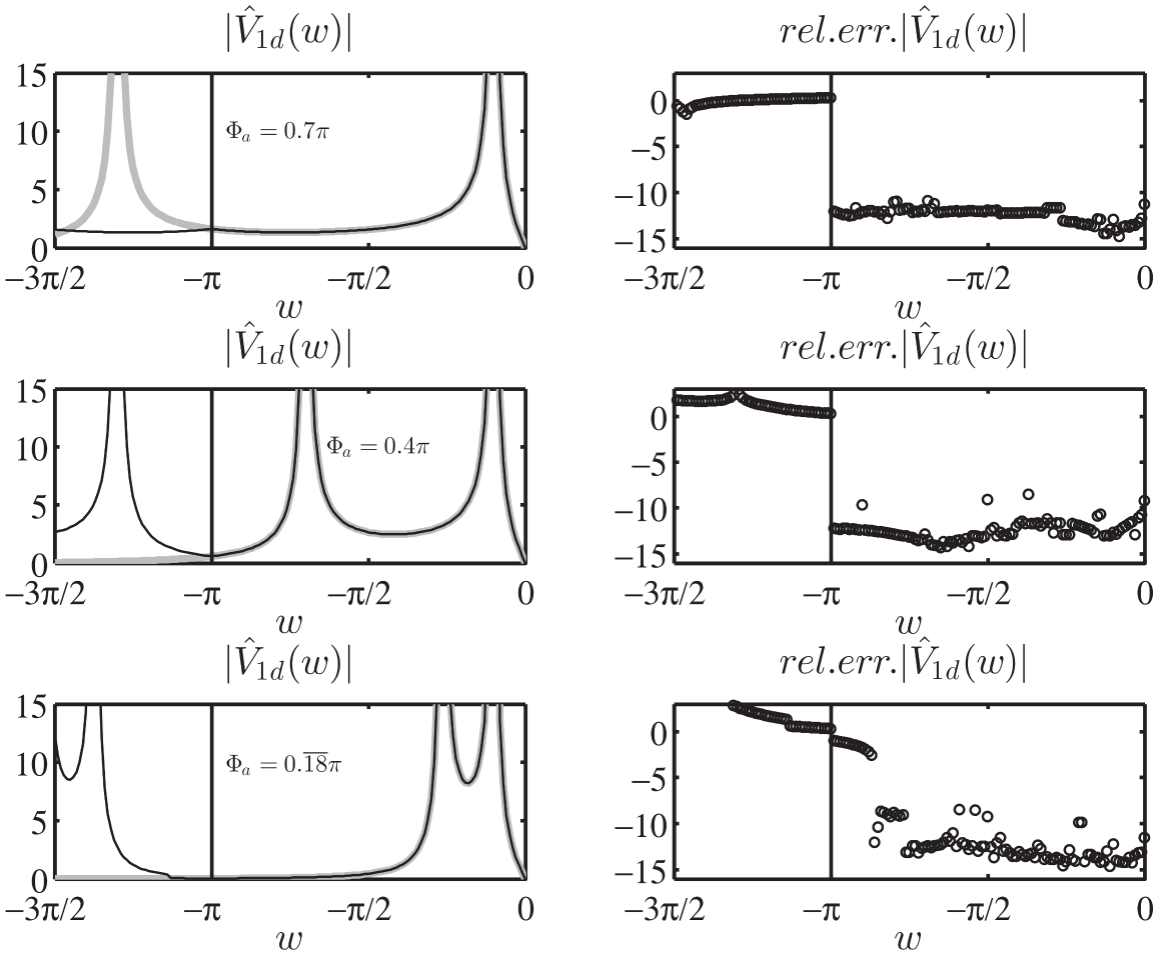
Validation of FIE along the real axis w, by substituting *η* = −*k* cos *w*. Left: absolute value of $|{\hat{V}}_{1d}\left(w\right)|$ (exact = thick solid gray line, approximate = solid black line). Right: relative error in log_10_ scale obtained from the approximate expression of *V*_1*d*_(*η*) for *η* = −*k* cos *w* given by while substituting the exact expression of *V*_1+_(*η*) and performing the numerical integration. Test cases are relative to different sets of parameters: $\Phi_{a},=0.7\pi ,0.4\pi ,0.\bar{18}\pi$, Φ_*b*_ = 1.2*π*, *φ*_*o*_ = 0.1*π*. We recall that −*π* < *w* < 0 is the portion of real axis of w plane in the proper sheet of *η* plane.

Fig 22 shows on the left the plots of the exact spectrum ${\hat{V}}_{1d}\left(w\right)$ and of the approximate spectrum ${\hat{V}}_{1d}\left(w\right)$ obtained through the FIE for the three characteristic test cases already used int the previous Sections: PEC wedges with $\Phi_{a}=\left[0.7\pi ,0.4\pi ,0.\bar{18}\pi \right]$, Φ_*b*_ = 1.2*π*, *φ*_*o*_ = 0.1*π*. On the right the same figure the relative error is plotted in log_10_ scale. We note that the spectrum ${\hat{V}}_{1d}\left(w\right)$ is valid for at least any real −*π* < *w* < 0 and the relative errors are scattered and are approximately around 10^−16^ ÷ 10^−11^ except for test 3 near −*π* where we have applied the approximation discussed in the previous Section for the kernel singularities that go into the lower half *η* plane.

To analytically extend the solution in the entire complex plane w we need to resort to difference equations obtained from the Wiener-Hopf formulation of the problem by eliminating the minus unknowns, as reported in [30].

Once the spectra are known, the procedure to obtain the total field from the spectra is reported in several papers, see for example in the context of impenetrable wedge structure [30]: first we apply the SDP (Steepest Descent Path) method to get GO contributions and GTD diffraction coefficients, then the UTD theory is applied to obtain the total field around the edge of the wedge.

To test the full procedure in particular for critical small aperture angles, we have numerically solved the FIE and computed the total field after the estimation of the GO and UTD component.

**Fig 23:**
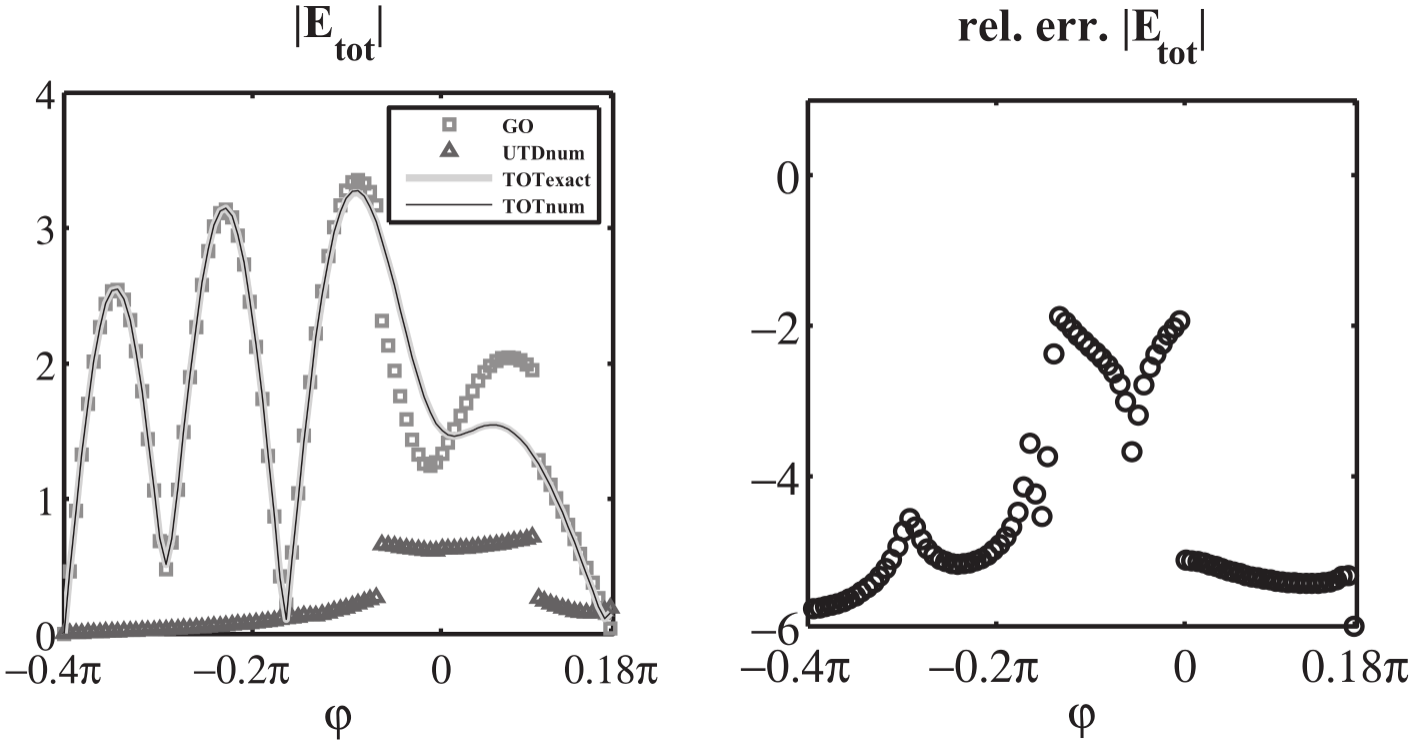
Validation through the total field for the wedge problem. Left: Total field obtained from the spectra in terms of GO and UTD components for the test case $\left[\Phi_{a},\Phi_{b},\varphi_{o}\right]=\left[0.\bar{18}\pi ,0.4\pi ,0.1\pi \right]$. Right: Relative error on the computation of the total field in log_10_ scale of the numerical solution obtained with coarse numerical parameters *A* = 20, *h* = 0.025.

Fig 23 reports on the left the total field obtained from the spectra in terms of GO and UTD components for a significant test case with parameters $\left[\Phi_{a},\Phi_{b},\varphi_{o}\right]=\left[0.\bar{18}\pi ,0.4\pi ,0.1\pi \right]$. Note that region 2 is hyper-acute and region 1 is acute to highlight all the properties in terms of kernel singularities. On the right of Fig 23 the relative error with respect to the exact solution is reported in log_10_ scale. For the numerical solution we have selected a simple sample and hold quadrature with coarse parameters *A* = 20, *h* = 0.025. The loss of precision is due to the approximate handling of kernel singularities that go into the lower half *η* plane for hyper-acute aperture angles. For non-hyper acute aperture angles the accuracy is perfect and the relative errors approximately goes to machine precision.

The validation is asserted by the complete overlap of the total field obtained with exact spectra and the approximate spectra.

Below, we list the mathematical and physical properties of the significant test case.

From a mathematical point of view, region 2 has an aperture angle $\Phi_{a}=0.\bar{18}\pi$, thus it is hyper-acute. According to Table 1 we have to consider *p*_1_ and *p*_2_ in Φ_*a*_ as structural singularities while integrating along the real axis *η* and we have to consider $p_{1},\;p_{2},\;p_{3},\;p_{4},\;p_{-1},\;{\tilde{p}}_{1}$ for the analytical extension in −*π* < *w* < 0. Moreover, we need to pay attention to the properties of $p_{1},\;{\tilde{p}}_{1}$. On the contrary, according to Table 1, region 1 is acute since Φ_*b*_ = 0.4*π* and therefore we have to consider *p*_1_ in Φ_*b*_ as structural singularity while integrating along the real axis *η* and for the analytical extension in −*π* < *w* < 0.

From a physical point of view, we note that the GO contributions are [in the following we report the outgoing angle from the edge of the wedge for each wave]: the incident wave *ϕ*_*I*_ = −*π* + *ϕ*_*o*_ = −0.9*π*, the wave due to the first reflection from face A $\phi_{RA}=-\pi +2\Phi_{a}-\phi_{o}=-0.7\bar{63}\pi$, the wave due to the first reflection from face B *ϕ*_*RB*_ = *π* − 2Φ_*b*_ − *ϕ*_*o*_ = 0.1*π* with azimuthal support −Φ_*b*_ < *ϕ* < *ϕ*_*RB*_ and the wave due to the first reflection from face A and the second from face B $\phi_{RBRA}=\pi -2\Phi_{b}-2\Phi_{a}+\phi_{o}=-0.0\bar{63}\pi$ with azimuthal support −Φ_*b*_ < *ϕ* < *ϕ*_*RBRA*_. UTD estimation compensate the two shadow regions.

From a mathematical-physical point of view the GO contribution are related to the spectral poles Eq (57): [*w*_*I*_, *w*_*RA*_, *w*_*RB*_, *w*_*RBRA*_] = [−*ϕ*_*o*_, −2Φ_*a*_ + *ϕ*_*o*_, −2Φ_*b*_ − *ϕ*_*o*_, −2Φ_*b*_ − 2Φ_*a*_ + *ϕ*_*o*_]. Note that only the poles *w*_*I*_, *w*_*RA*_ are captured in −*π*/2 < *w*, 0 and gives contributions in the FIE.

## 7 A novel practical problem: a PEC wedge above a PEC plane

In order to demonstrate the efficacy and the novelty of the proposed methodology we present an original test case. The procedure starts from the GWHE formulation of the problem whose solution is obtained via FIEs that are derived through the help of network modelling. The results show the capability of network modelling to deals with complex problems and show the convergence, the efficiency and the efficacy of the proposed method estimating the electromagnetic far field in terms of Geometrical Optics (GO) components and uniform diffracted components (UTD).

**Fig 24:**
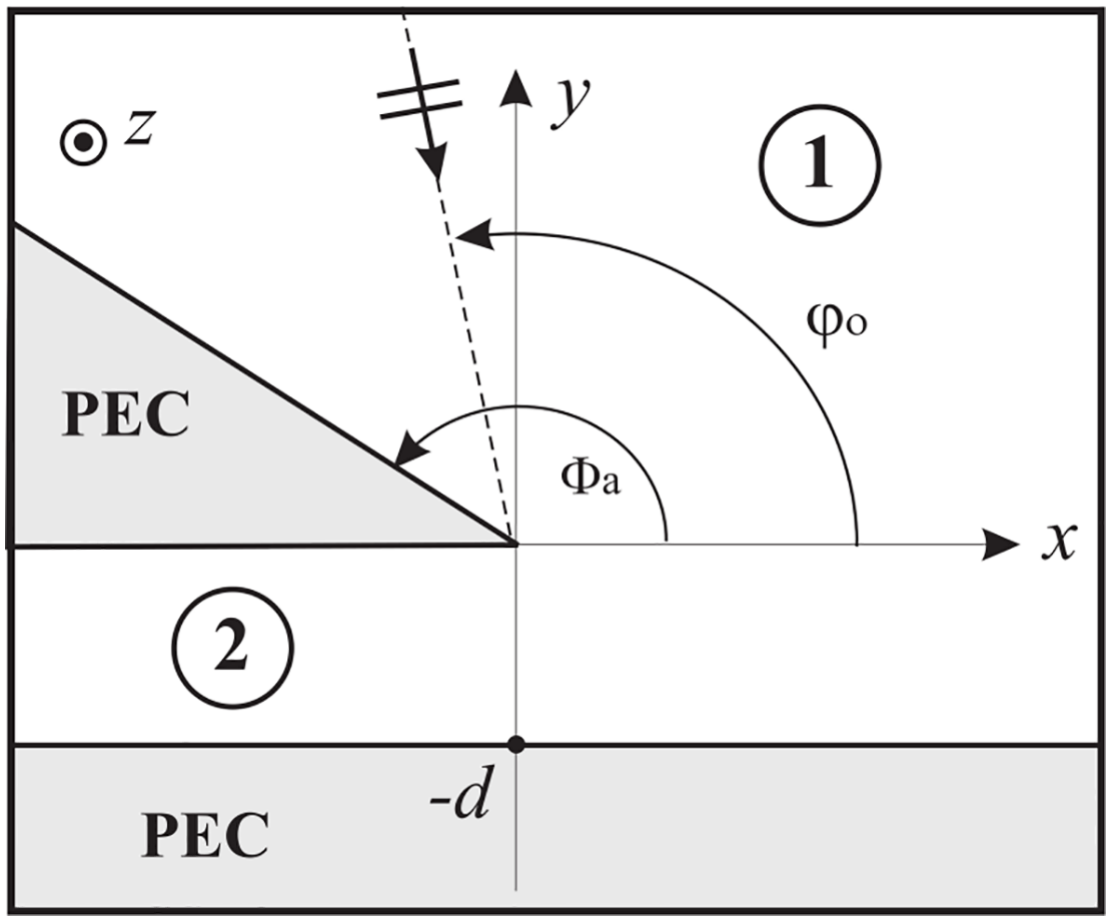
A PEC wedge above a PEC plane.

The problem is constituted by the scattering of an *E*_*z*_ polarized plane wave by a PEC wedge above a PEC plane in a homogenous medium, see Fig 24. In Fig 24 the wedge structure is delimited by the PEC faces *a* at *φ* = Φ_*a*_ and *b* at *φ* = −Φ_*b*_ = −*π* and is located above a PEC plane at a distance *d*. The structure is illuminated by an *E*_*z*_-polarized plane wave with direction *φ* = *φ*_*o*_ (0 ≤ *φ*_*o*_ < Φ_*a*_). The domain of the problem is subdivided into two regions: the angular region 1 (0 ≤ *φ* < Φ_*a*_), the layer region 2 (−*d* ≤ *y* ≤ 0). The two regions are filled with a low-dense lossless isotropic homogeneous dielectric medium, which, without loss of completeness, can be considered free space with permittivity *ε*_*o*_, permeability *μ*_*o*_ and propagation constant $k=\omega \sqrt{\mu_{o}\;\varepsilon_{o}}$.

Let us consider the angular region 1. From [13] we have the GWHE

$$\begin{array}{c} Y_{c}\left(\eta \right)\;V_{1+}\left(\eta \right)-I_{1+}\left(\eta \right)=-I_{a+}\left(-m\right) \end{array}$$ (77)

where $Y_{c}\left(\eta \right)=1/Z_{c}\left(\eta \right)=\frac{\xi (\eta )}{k\;Z_{o}}$ is the free space spectral admittance and $\xi \left(\eta \right)=\sqrt{k^{2}-\eta^{2}}$ is the free space spectral propagation constant and

$$\begin{array}{c} I_{a+}\left(-m\right)=\int_{0}^{\infty }H_{\rho }\left(\rho ,\Phi_{a}\right)e^{-jm\;\rho }d\rho \end{array}$$ (78)

with *m* = −*η* cos Φ_*a*_ + *ξ*(*η*)sinΦ_*a*_.

Region 2 is a finite layer and, according to the theory of layered regions (see for example [3] and [2]), a transmission line model holds for the quantities

$$\begin{array}{c} \left\{ \begin{array}{c} v\left(\eta ,y\right)=\int_{-\infty }^{\infty }E_{z}\left(x,y\right)e^{j\eta \;x}dx\\ i\left(\eta ,y\right)=\int_{-\infty }^{\infty }H_{x}\left(x,y\right)e^{j\eta \;x}dx \end{array},\;\left(y\leq 0\right)\right. \end{array}$$ (79)

From transmission line theory we have the following spectral impedance relation:

$$\begin{array}{c} Y(\eta )v(\eta ,0)=-i(\eta ,0) \end{array}$$ (80)

The admittance *Y*(*η*) = 1/*Z*(*η*) is the one seen at *y* = 0

$$\begin{array}{c} Y\left(\eta \right)=-jY_{c}\left(\eta \right)\cot\left(\xi \left(\eta \right)d\right) \end{array}$$ (81)

Taking into account that

$$\begin{array}{c} \begin{array}{c} v\left(\eta ,0\right)=V_{1+}\left(\eta \right),\;\\ i\left(\eta ,0\right)=I_{1-}\left(\eta \right)+I_{1+}\left(\eta \right), \end{array} \end{array}$$ (82)

it yields the following Wiener-Hopf equation

$$\begin{array}{c} Y\left(\eta \right)V_{1+}\left(\eta \right)+I_{1-}\left(\eta \right)+I_{1+}\left(\eta \right)=0 \end{array}$$ (83)

Moreover, according to the transmission line theory we have

$$\begin{array}{c} v\left(\eta ,y\right)=\frac{\sin(\xi \;(d+y))}{\sin(\xi \;d)}v_{\eta }\left(0\right),\;-d\leq y\leq 0 \end{array}$$ (84)

with *Z*_*c*_ and *ξ* functions of *η*.

The reflection coefficients for the incident wave from the PEC plane respectively at *y* = −*d* and *y* = 0 are

$$\begin{array}{c} \Gamma_{RD}=-1,\;\Gamma_{o}=-e^{-2j\xi (\eta_{o})d} \end{array}$$ (85)

### 7.1 Network model

Region 1 is an angular region that can be modelled via Norton Eq (72) in *η* plane or Eq (62) in *w* plane by considering that the GO poles are derived by a new configuration where the face *b* of the angular region is substituted by a PEC plane.

With an alternative procedure we note that, Eq (72) and all the other equations reported in the previous Sections for the angular region can be obtained directly from the GWHE Eq (77) according to the following steps:

1. transformation of Eq (77) into a classical WH equation in *α* plane via the mapping Eq (67)
2. elimination of minus unknown *I*_2+_(−*m*) in *α* plane via Cauchy integration that yields the FIE
3. application of mapping Eq (67) and *η* = −*k* cos *w*
4. contour warping from the real axis of *η* plane to imaginary axis of *η* plane and then to *M*_1_ and *M*

This procedure has been effectively applied without reference to network modelling to several wedge problems in $\bar{w}$ plane, see [30, 31].

The main drawback to derive the FIE in *η* plane from the GWHE Eq (77) is that the above procedure do not highlights the validity regions of the numerically approximate formulations in particular for small aperture angles that yield to sectionally analytic representations [44]. In particular the kernel singularities and their relevant regions are cumbersome to treat directly in *η* plane.

Let us consider region 2.

We apply Cauchy integration to Eq (83) along the contour *γ*_1*η*_ which is the *smile* integration line defined in [32] and [3]. We obtain for η∈R

$$\begin{array}{c} \frac{1}{2\pi j}\int_{\gamma_{1\eta }}\frac{Y\left(\eta^{\prime }\right)V_{1+}\left(\eta^{\prime }\right)+I_{1+}\left(\eta^{\prime }\right)}{\eta^{\prime }-\eta }d\eta^{\prime }=0 \end{array}$$ (86)

since *I*_1−_(*η*) is regular in $\text{Im}[\eta^{\prime }]\leq 0$ and thus $\frac{1}{2\pi j}\int_{\gamma_{1\eta }}\frac{I_{1-}\left(\eta^{\prime }\right)}{\eta^{\prime }-\eta }d\eta^{\prime }=0$.

By applying Cauchy integration to *V*_1+_(*η*) and *I*_1+_(*η*) along the contour *γ*_2*η*_ which is the *frown* integration line defined in [32] and [3] we obtain

$$\begin{array}{c} \frac{1}{2\pi j}\int_{\gamma_{1\eta }}\frac{V_{1+}\left(\eta^{\prime }\right)}{\eta^{\prime }-\eta }d\eta^{\prime }=-S_{v+}^{\left(\nu \right)}\left(\eta \right)\;\frac{1}{2\pi j}\int_{\gamma_{1\eta }}\frac{I_{1+}\left(\eta^{\prime }\right)}{\eta^{\prime }-\eta }d\eta^{\prime }=-S_{i+}^{\left(\nu \right)}\left(\eta \right) \end{array}$$ (87)

where $S_{v+}^{\left(\nu \right)}\left(\eta \right)$ and $S_{i+}^{\left(\nu \right)}\left(\eta \right)$ are known functions that depends on the Geometrical Optics poles related to the entire problem and located in the upper-half *η* plane.

Combining Eqs (86) and (87) and taking into account that

$$\begin{array}{c} \begin{array}{c} \frac{1}{2\pi j}\int_{\gamma_{1}}^{}\frac{Y_{}\left(\eta^{\prime }\right)V_{1+}\left(\eta^{\prime }\right)}{\eta^{\prime }-\eta }d\eta^{\prime }-\frac{1}{2\pi j}\int_{\gamma_{2}}^{}\frac{Y_{}\left(\eta \right)V_{1+}\left(\eta^{\prime }\right)}{\eta^{\prime }-\eta }d\eta^{\prime }=\frac{1}{2\pi j}\int_{-\infty }^{\infty }\frac{\left[Y_{}\left(\eta^{\prime }\right)-Y_{}\left(\eta \right)\right]V_{1+}\left(\eta^{\prime }\right)}{\eta^{\prime }-\eta }d\eta^{\prime }+Y\left(\eta \right)V_{1+}\left(\eta \right) \end{array} \end{array}$$ (88)

we obtain the integral equation:

$$\begin{array}{c} +I_{1+}\left(\eta \right)=-Y_{D}\left(\eta \right)\cdot V_{1+}\left(\eta \right)+I_{D}\left(\eta \right) \end{array}$$ (89)

where $Y_{D}\left(\eta \right)$ is the operator

$$\begin{array}{c} Y_{D}\left(\eta \right)=Y\left(\eta \right)+\frac{1}{2\pi j}\int_{-\infty }^{\infty }\frac{Y\left(\eta^{\prime }\right)\;-Y\left(\eta \right)\;}{\eta^{\prime }-\eta }\left[...\right]d\eta^{\prime } \end{array}$$ (90)

In particular for the problem under examination *I*_*D*_(*η*) is

$$\begin{array}{c} I_{D}\left(\eta \right)=\sum_{\nu }\left[Y\left(\eta \right)S_{v_{+}}^{\left(\nu \right)}\left(\eta \right)+S_{i_{+}}^{\left(\nu \right)}\left(\eta \right)\right] \end{array}$$ (91)

where (*ν*) is related to the GO components with incident angles 0 < *φ*_*ν*_ < min[Φ_*a*_, *π*/2] (incident wave, reflected wave from face a and multiple reflected waves from face a when Φ_*a*_ is acute) and

$$\begin{array}{c} S_{v_{+}}^{\left(\nu \right)}\left(\eta \right)=\frac{R_{v}^{\left(\nu \right)}}{\eta -\eta_{\nu }}u\left(\frac{\pi }{2}-\varphi_{\nu }\right),\;S_{i_{+}}^{\left(\nu \right)}\left(\eta \right)=\frac{R_{i}^{\left(\nu \right)}}{\eta -\eta_{\nu }}u\left(\frac{\pi }{2}-\varphi_{\nu }\right) \end{array}$$ (92)

with *η*_*ν*_ = −*k* cos(*φ*_*ν*_) that corresponds to the pole generated by the GO waves *ν*, with $\Gamma_{\nu }=\frac{Y_{c}\left(\eta_{\nu }\right)-Y\left(\eta_{\nu }\right)}{Y_{c}\left(\eta_{\nu }\right)+Y\left(\eta_{\nu }\right)}$ that is the reflection coefficient from the PEC plane and $R_{v}^{\left(\nu \right)}=j\left(1+\Gamma_{\nu }\right)E_{z},\;R_{i}^{\left(\nu \right)}=-j\left(1-\Gamma_{\nu }\right)\frac{E_{z}}{Z_{o}}\sin\left(\varphi_{\nu }\right)$.

Eq (89) is the Norton representation of region 2 with generalized admittance $Y_{D}\left(\eta \right)$ and (short-circuit) Norton current *I*_*D*_(*η*). The Norton generator is controlled by the GO terms of the entire problem.

**Fig 25:**
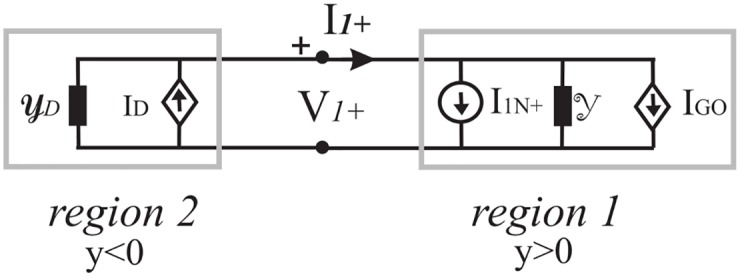
Network model of the test problem reported in Fig 24.

Fig 25 reports the network model of the problem represented through the Norton Eqs (72) and (89) in *η* plane.

### 7.2 From network model to the solution

From circuital analysis of the network represented in Fig 25 we obtain

$$\begin{array}{c} \left(Y\left(\eta \right)+Y_{D}\left(\eta \right)\right)\cdot V_{1+}\left(\eta \right)=I_{tot}\left(\eta \right) \end{array}$$ (93)

where *I*_*tot*_(*η*) is the contribution of the currents due to the generators *I*_1*N*+_ Eq (74), *I*_*GO*_ Eq (58) and *I*_*D*_ Eq (91), $Y_{D}\left(\eta \right)$ and Y(η) are the operator admittances respectively for the layer region 2 (see Eq (90)) and for the angular region 1 (see Eq (73)).

Eq (93) is a Fredholm integral equation with unknown *V*_1+_(*η*), see Appendix. In normal form the explicit expression of Eq (93) is

$$\begin{array}{c} \begin{array}{c} V_{1+}\left(\eta \right)+\frac{Z_{e}\left(\eta \right)}{2\pi j}\int_{-\infty }^{\infty }\left(\frac{Y_{c}\left(\eta^{\prime }\right)\;}{\alpha \left(\eta^{\prime }\right)-\alpha \left(\eta \right)}\frac{d\alpha }{d\eta^{\prime }}-\frac{Y_{c}\left(\eta \right)\;}{\eta^{\prime }-\eta }+\sum_{n}\frac{q_{n}\left(\eta \right)}{\eta^{\prime }-{\text{p}}_{n}\left(\eta \right)}u\left(\frac{\pi }{2}-n\Phi_{a}\right)\right)V_{1+}\left(\eta^{\prime }\right)d\eta^{\prime }+\\ \;\;\;+\frac{Z_{e}\left(\eta \right)}{2\pi j}\int_{-\infty }^{\infty }\frac{Y\left(\eta^{\prime }\right)\;-Y\left(\eta \right)\;}{\eta^{\prime }-\eta }V_{1+}\left(\eta^{\prime }\right)d\eta^{\prime }=V_{tot}\left(\eta \right) \end{array} \end{array}$$ (94)

where

$$\begin{array}{c} Z_{e}\left(\eta \right)={\left(Y_{c}\;+Y_{}\left(\eta \right)\right)}^{-1}=j\frac{kZ_{o}}{\xi (\eta )}\sin\left(\xi \left(\eta \right)d\right)e^{-j\xi (\eta )d} \end{array}$$ (95)

and *V*_*tot*_(*η*) = *Z*_*e*_(*η*)*I*_*tot*_(*η*).

Since Eq (94) is a FIE (see Appendix), it has good convergence properties. The presence of infinite integration interval can be overcome through suitable mappings to reduce the infinite integration interval to a finite one with optimal functional properties of the integrand [47] or truncating the integration interval due to the convergence behaviour of the spectral unknowns [43].

Since the kernels of Eq (94) present well suited behavior, we use a simple sample and hold quadrature scheme with truncation parameter *A* and step parameter *h* to obtain accurate and stable numerical solutions as already discussed and done for the wedge problem, see previous Section 6.6 and reference therein.

The coefficient of the excited *TE*_*x*_ modes *c*[−*η*_*i*_] with $i\in N_{0}$ inside the planar guide −*d* < *y* < 0, *x* < 0 are computed through the residue of *I*_1−_(*η*) in −*η*_*i*_ which are the propagation constants of the excited regressive *TE*_*x*_ modes where

$$\begin{array}{c} \eta_{i}=\sqrt{k^{2}-{\left(\frac{i\;\pi }{d}\right)}^{2}} \end{array}$$ (96)

We note from Eq (83) that

$$\begin{array}{c} i\left(\eta ,0\right)=I_{1+}\left(\eta \right)+I_{1-}\left(\eta \right)=-Y\left(\eta \right)V_{1+}\left(\eta \right) \end{array}$$ (97)

with *Y*(*η*) defined in Eq (81).

Since we can estimate a priori that the spectrum of *I*_1+_(*η*) does not contain −*η*_*i*_ poles, we observe that residues of *I*_1−_(*η*) can be computed from approximated spectra of *V*_1+_(*η*).

In particular, from Eq (97) we compute

$$\begin{array}{c} I_{1-}\left(\eta \right)=\sum_{i=1}^{\infty }\frac{c[-\eta_{i}]}{\eta +\eta_{i}} \end{array}$$ (98)

with

$$\begin{array}{c} c\left[\eta \right]=-j\frac{k^{2}-\eta^{2}}{dkZ_{o}\eta }V_{1+}\left(\eta \right) \end{array}$$ (99)

The numerical solution of the integral equations provide an analytical element of the spectra *V*_1+_(*η*), *I*_1+_(*η*), *I*_1−_(*η*) that is in the proper sheet of the *η*-plane or in a strip of *w* plane. To obtain the diffraction coefficients, in general we need to know the spectra also in the improper sheet of the *η* plane or in a region of *w* plane that corresponds to multiple sheets of *η* plane, in particular the real axis of *w* plane. Analytical continuation of the approximate spectra is possible by resorting to the original GWHEs of the Problems (77) and (83) reformulated in the *w*-plane that yields recurrence relations/difference equations in *w* plane capable of extend the known approximate spectra to the whole complex plane *w* [4]. The recurrence equations for plus unknowns are obtained via the elimination of minus unknowns exploiting the fact that plus unknowns are even functions of *w*.

Analytical extensions of ${\hat{V}}_{1+}\left(w\right)$ and ${\hat{I}}_{1+}\left(w\right)$ are obtained via:

$$\begin{array}{c} {\hat{V}}_{1+}\left(w\right)=\left\{ \begin{array}{cc} {\hat{V}}_{1+}^{\left(num\right)}\left(w\right) & -\pi \leq \text{Re}[w]\leq 0\\ -{\hat{V}}_{1+}\left(-w\right) & \text{Re}[w]>0\\ Eq(102) & \text{Re}[w]<-\pi \end{array}\right. \end{array}$$ (100)

$$\begin{array}{c} {\hat{I}}_{1+}\left(w\right)=\left\{ \begin{array}{cc} {\hat{I}}_{1+}^{\left(num\right)}\left(w\right) & -\pi \leq \text{Re}[w]\leq 0\\ {\hat{I}}_{1+}\left(-w\right) & \text{Re}[w]>0\\ Eq(103) & \text{Re}[w]<-\pi \end{array}\right. \end{array}$$ (101)

with

$$\begin{array}{c} {\hat{V}}_{1+}\left(w\right)=\frac{{\hat{V}}_{1d}\left(w+2\Phi_{a}\right)+Z_{o}\left[{\hat{I}}_{1+}\left(w+2\pi \right)-{\hat{I}}_{1+}\left(w+2\Phi_{a}\right)+\hat{Y}\left(-w-2\pi \right){\hat{V}}_{1+}\left(w+2\pi \right)\right]}{-\sin\left(w\right)+Z_{o}\hat{Y}\left(w\right)} \end{array}$$ (102)

$$\begin{array}{c} {\hat{I}}_{1+}\left(w\right)=\frac{\sin\left(w\right)\left[{\hat{I}}_{1+}\left(w+2\pi \right)+\hat{Y}\left(-w-2\pi \right){\hat{V}}_{1+}\left(w+2\pi \right)\right]+\hat{Y}\left(w\right)\left[-Z_{o}{\hat{I}}_{1+}\left(w+2\Phi_{a}\right)+{\hat{V}}_{1d}\left(w+2\Phi_{a}\right)\right]}{\sin\left(w\right)-Z_{o}\hat{Y}\left(w\right)} \end{array}$$ (103)

where $\hat{Y}\left(w\right)=Y\left(-k\cos\left(w\right)\right)$ and where ${\hat{V}}_{1d}\left(w\right)$ has been used to get more compact expressions.

As stated in the previous Sections for complex problem we need a starting spectra in −*π* < *w* < 0 to compute the far field components.

Once the complete spectrum is obtained, diffraction coefficients are then calculated through SDP method using the extended spectra in *w*, see for example [8, 30, 31].

**Fig 26:**
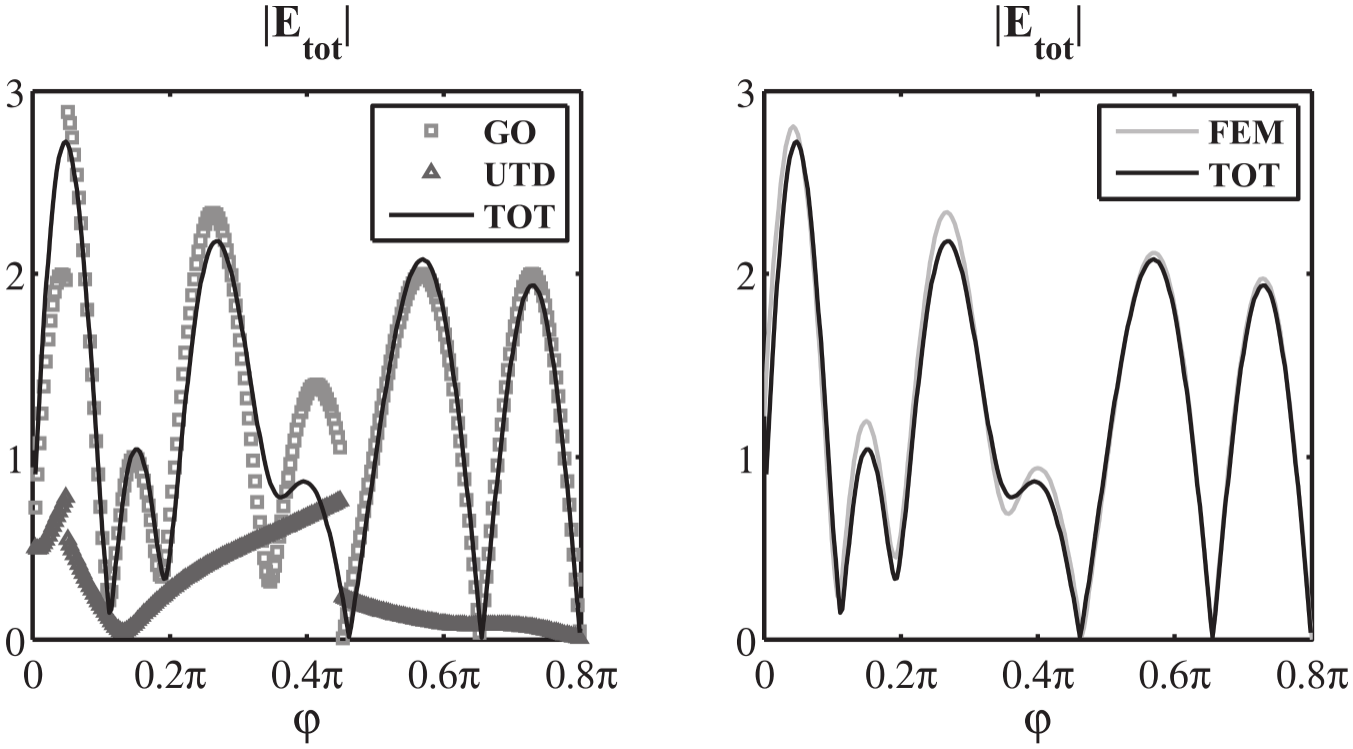
Total field. Left: Total field obtained from the approximate spectra in terms of GO and UTD components for the test case of Fig 24 with parameters [Φ_*a*_, *φ*_*o*_, *d*] = [0.8*π*, 0.55*π*, 0.55*λ*]. The numerical solution is obtained with integration parameters *A* = 40, *h* = 0.2 according to the kernel behavior. Right: Comparison of Total Field with FEM solution as described in the main text.

Fig 26 reports on the left the total field obtained from the approximate spectra in terms of GO and UTD components for the test case of Fig 24 with [Φ_*a*_, *φ*_*o*_, *d*] = [0.8*π*, 0.55*π*, 0.55*λ*] where *λ* is the wavelength. The numerical solution is obtained with integration parameters *A* = 40, *h* = 0.2 according to the kernel behavior. For the explicit expression of the total field we make reference to Section IV of [31] where the GO and UTD components are estimated from the spectra ${\hat{V}}_{1+}\left(w\right)$ and ${\hat{I}}_{1+}\left(w\right)$.

In this problem we obtain a GO contributions of incident wave, reflected wave from face A with outgoing direction Φ_*RA*_ = −*π* + 2Φ_*a*_ − *ϕ*_*o*_ = 0.05*π* with support *ϕ*_*RA*_ < *ϕ* < Φ_*a*_ and reflected wave from the PEC plate with outgoing direction Φ_*RD*_ = *π* − *ϕ*_*o*_ = 0.45*π* with support 0 < *φ* < *ϕ*_*RD*_.

On the right side of Fig 26 we compare our solution with the one obtained by in-house FEM [48] with the following setup: region truncated at 9*λ* with perfectly matched layer and discretization via triangles with max side length of *λ*/8. We note that the FEM solution is less accurate in particular for forward far field directions (small *φ*) due to the uncorrect modelling of the termination of the waveguide. In fact this uncorrect modelling generates small spurious reflected waves that radiate again. We also note that perfectly matched layer should entirely surround an object in classical scheme.

For the same problem we have estimated the field inside the waveguide by computing *c*[−*η*_*i*_] Eq (99). Table 2 reports some of the values of *η*_*i*_ and the corresponding *c*[−*η*_*i*_] normalized to *Z*_*o*_. Note that with the problem’s parameters we have just one propagating mode.

**Table 2:** Properties of *TE*_*x*_ modes.

i	*η*_*i*_	*Z*_*o*_*c*[−*η*_*i*_]
1	0.416598 − *j*0.00024004	0.259246 + *j*0.40961
2	0.0000658553 − *j*1.51848	−0.0531907 − *j*0.179129
3	0.0000394116 − *j*2.53732	−0.00938898 − *j*0.125368
10	0.0000110672 − *j*9.03574	0.0167714 − *j*0.0592233
20	5.50834 × 10^−6^ − *j*18.1543	0.0178825 − *j*0.0412463
100	1.10007 × 10^−6^ − *j*90.9036	0.0121274 − *j*0.0210972

Through modal expansion, we can build the *H*_*x*_(*x*, *y*) field:

$$\begin{array}{c} H_{x}\left(x,y\right)=j\sum_{i=1}^{N_{i}}c\left[-\eta_{i}\right]\cos\left(\frac{i\pi }{d}y\right)e^{j\eta_{i}x} \end{array}$$ (104)

**Fig 27:**
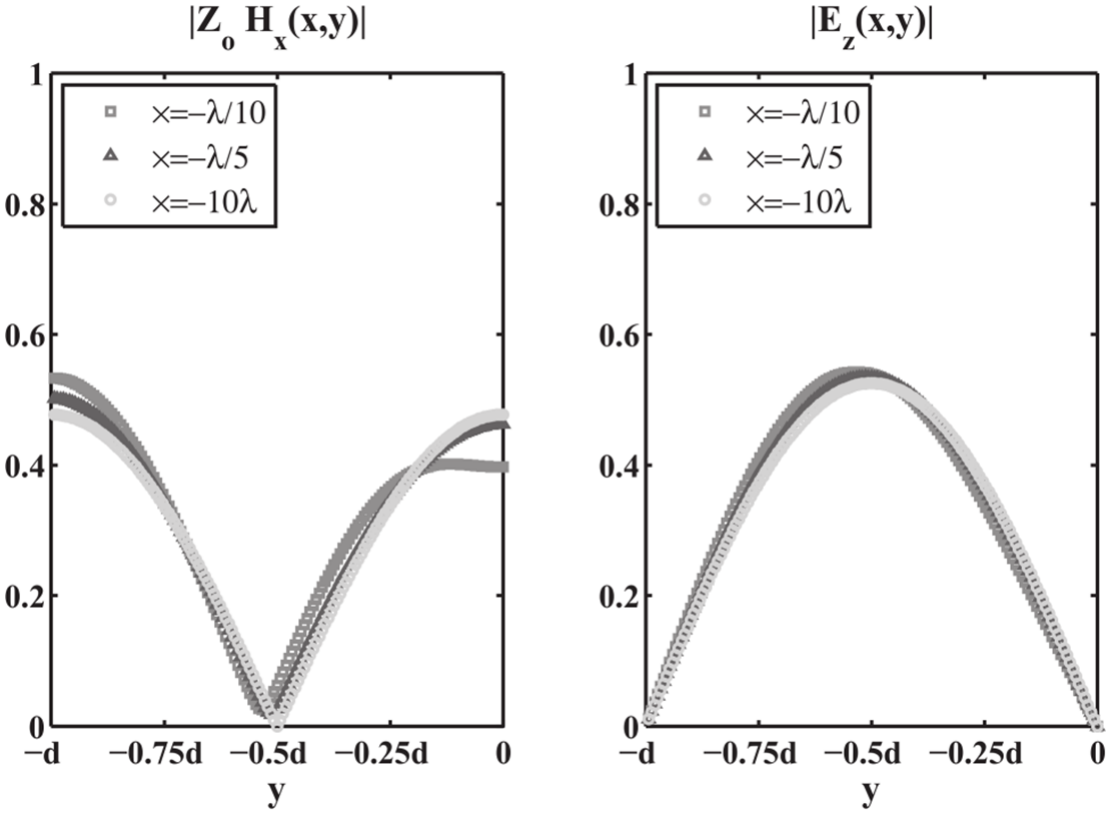
Modal field. Modal field *Z*_*o*_*H*_*x*_(*x*, *y*) and *E*_*z*_(*x*, *y*) at *x* = [−*λ*/10, −*λ*/5, −10*λ*] with *N*_*i*_ = 100 for the test case of Fig 24 with parameters [Φ_*a*_, *φ*_*o*_, *d*] = [0.8*π*, 0.55*π*, 0.55*λ*].

Fig 27 shows the plot of *H*_*x*_(*x*, *y*) at *x* = [−*λ*/10, −*λ*/5, −10*λ*] with *N*_*i*_ = 100, although *N*_*i*_ = 20 is sufficient for convergence especially leaving the edge profile (the origin of the cartesian coordinates in Fig 24). Details on the implementation of this practical problem with arbitrary aperture angle Φ_*a*_ and multiple media are going to be reported in [46].

## 8 Conclusions

In this paper we have described in detail the new developments of the Generalised Wiener-Hopf technique, by augmenting the method with the aid of network models. In this way, it is possible to tackle a new class of canonical problems in the theory of diffraction, namely wedges together with planar layers. This technique extends the applications of asymptotic methods. In particular we have presented how network representation and approach help to order and to systematize the steps necessary to obtain the Fredholm integral equations that model complex diffraction problems.

The paper reports in detail the network representations for an angular region (see Fig 12) with *E*_*z*_ polarization, i.e. *H*_*z*_ = 0 and ∂/∂*z* = 0. The full extension to more general network representations (Norton and Thevenin) for arbitrary homogeneous angular regions will be considered in future papers. The methodology can be immediately extended in presence of acoustic waves in fluids taking into account in Eq (1) that the voltage ${\dot{V}}_{+}$ is related to the Laplace transform of pressure *p*(*ρ*, *φ*) and the current ${\dot{I}}_{+}$ to the Laplace transform of ς∂p(ρ,φ)/∂φ with ς being the density of the fluid, see for instance [47]. In particular Section 7 solves also the acoustic problem where the region 1 and 2 are bounded by acoustically soft walls. Conversely in presence of elastic waves in isotropic solids, even though it is possible to reduce the field problem to two independent equations Eq (14), the boundary conditions are generally very intricate and difficult (see for example [49]) and thus the derivation of network representations is not straightforward for this physics.

## Appendix: Fredholm properties of the integral Eq (93)

In this Appendix we briefly justify the properties of Eq (93). If we approximate Eq (94) with an equation where the integration interval is limited (for instance −*A* ≤ *η* ≤ *A* with real *η*), provided *A* sufficiently large, the solution of this approximate problem is very closed to the exact solution. In fact taking into account the Meixner condition near the edge of the wedge, we have that $V_{1+}\left(\eta \right)=O\left(\frac{1}{\eta^{1+c}}\right),\;c>0$ as *η* → ∞. It allows us to claim that we are dealing with an unknown that in practice is negligible out the interval −*A* ≤ *η* ≤ *A*. Due to its smoothness, the kernel integrated in the finite range −*A* ≤ *η* ≤ *A* is compact and it guarantees the convergence of the numerical solution via quadrature [43], whence we can correctly label Fredholm the integral Eq (93) when it is defined in the limited interval −*A* ≤ *η* ≤ *A* for every finite value of *A*.

Conversely to rigorously label as Fredholm the integral Eq (93), we must prove that the norm of the integral operator defined on the infinite integration contour is bounded. To ascertain this property, we need to introduce a suitable space of functions where we define Eq (93). We note that the Hilbert space is inadequate because in this space we have numerically tested that the kernel of Eq (94) is not quadratically integrable. Conversely a more suitable functional space for Eq (94) is suggested in [15] that deals with similar operators defined in the *w*-plane. This space is the generalized Hilbert space *M*_*μ*_ = *L*_2_(, *μ*(*η*)) where the weight *μ*(*η*) introduces a different definition of the scalar product with respect the space $L_{2}\left(R\right)$. However is remarkable that all the properties of the space $L_{2}\left(R\right)$ also hold in the space *M*_*μ*_. The kernel in the integral Eq (94) is constituted of the sum of the three smooth kernels which corresponds to three operators defined in the space *M*_*μ*_. Assuming the weight $\mu \left(\eta \right)=1/{\left(1+\frac{\eta^{2}}{k^{2}}\right)}^{1/4}$ numerical experiments have shown that the norms of these three operators are bounded.

## Basic conventions, notations and symbols

MKS units and *e*^*jωt*^ time variations are used in the paper. In Table 3 we report the Basic Conventions, Notations and Symbols.

**Table 3:** Basic conventions, notations and symbols used in the paper.

WH	Wiener-Hopf
GWH	Generalized Wiener-Hopf
FIE	Fredholm Integral Equation
GTD	Geometrical Theory of Diffraction
UTD	Uniform geometrical Theory of Diffraction
PEC	Perfect Electric Conductor
PMC	Perfect Magnetic Conductor
(*x*, *y*, *z*)	cartesian coordinates
(*ρ*, *φ*, *z*)	cylindrical coordinates
*u*(*x*)	unitstep function (Heaviside)
*sign*(*x*)	sign function
Φ_*a*_	aperture angle of the angular region
*φ*_*o*_	incident angle of the plane wave
*d*	layer depth in Section 7
*k*	propagation constant of free space
*Z*_*o*_ = 1/*Y*_*o*_	free space impedance and admittance
*s* = −*jη*	spectral planes of Laplace transform
*w*	spectral plane in Sommerfeld-Malyuzhinets(SM) method(*η* = −*k* cos *w*)
*t*	spectral plane for Kontorovich-Lebedev(KL) transform
${\dot{F}}_{+}\left(s,\varphi \right)$	Laplace transform(LT) in *s* with respect to *ρ* for a direction *φ*
*F*_+_(*η*, *φ*)	$={\dot{F}}_{+}\left(s=-j\eta ,\varphi \right)$
${\hat{F}}_{+}\left(w,\varphi \right)$	= *F*_+_(*η* = −*k* cos *w*, *φ*)
${\hat{F}}_{d}\left(w,\varphi \right)$	$=\sin w\;{\hat{F}}_{+}\left(w,\varphi \right)$
$\bar{F}\left(t,\varphi \right)$	$=M\{\hat{F}\left(w,\varphi \right)\}$ Malyuzhinets-Fourier(MF) transform
$\tilde{F}\left(\cdot ,\cdot \right)$	(tilde) abstractly refers to a general spectral domain (*η*, *s*, *wort*)
*M*	is the integration line *Re*[*w*] = 0
*M*_1_	is the integration line *Re*[*w*] = −*π*/2
*γ*	imaginary axis of *w* plane indented at *w*′ = *w* on the right side
*γ*_1*η*_	is the “smile” integration line in *η* plane, see [32] and [3]
*γ*_2*η*_	is the “frown” integration line in *η* plane, see [32] and [3]
*ξ*(*η*)	$=\sqrt{k^{2}-\eta^{2}}$ with *ξ*(0) = *k*
*α*(*η*)	$=-k\cos[\frac{\pi }{\Phi_{a}}\arccos\left(-\frac{\eta }{k}\right)]$
$p_{n}\left(\eta \right),{\tilde{p}}_{n}\left(\eta \right)$	$=\eta \cos2n\Phi_{a}-\sqrt{k^{2}-\eta^{2}}\sin2n\Phi_{a}$ kernel singularity lines
*q*_*n*_(*η*)	$=\frac{Y_{o}}{k}\left(\eta \sin2n\Phi_{a}+\sqrt{k^{2}-\eta^{2}}\cos2n\Phi_{a}\right)$
*A*, *h*	truncation and step parameters of sample and hold quadrature
*Y*_*c*_(*η*)	= *ξ*(*η*)/(*kZ*_*o*_) free space spectral admittance
*Y*(*η*)	= −*jY*_*c*_(*η*)cot(*ξ*(*η*)*d*)
Y(η)	admittance operator of angular region
$Y_{D}\left(\eta \right)$	admittance operator of layer region in Section 7
*I*_*tot*_(*η*)	equivalent current due to sources in Section 7
*η*_*i*_	progressive *TE*_*x*_ mode propagation constants in Section 7
*c*[−*η*_*i*_]	coefficient of the excited *TE*_*x*_ modes in Section 7
